# Supplementary material for: Intensified screening for SARS-CoV-2 in 18 emergency departments in the Paris metropolitan area, France (DEPIST-COVID): A cluster-randomized, two-period, crossover trial
Source: PLoS Med. 2023 Dec 7;20(12):e1004317. doi: 10.1371/journal.pmed.1004317 (PMC10735176; doi:10.1371/journal.pmed.1004317)
Supplement: S1 File — Appendix A. Study protocol including the initial statistical analysis plan (SAP) as submitted to French data authorities, the final SAP, a summary of the changes in the SAP, and the DEPIST-COVID questionnaire. Appendix B. Figure Geographical location of the 18 emergency departments involved in the DEPIST-COVID trial (Paris metropolitan area). Fig B1. Geographical location of the 18 emergency departments involved in the DEPIST-COVID trial (Paris metropolitan area). References: R Core Team (2022). R: A language and environment for statistical computing. R Foundation for Statistical Computing, Vienna, Austria. URL https://www.R-project.org/. Hijmans, R.J. (2022). raster: Geographic Data Analysis and Modeling. R package version 4.1.3. https://CRAN.R-project.org/package=raster. Appendix C. Table Emergency department characteristics and study period duration. Table C1. Emergency department characteristics and study period duration. Data are presented as numbers or No 0/Yes 1. ED: Emergency department. aAnnual patient admissions included pediatric admissions. bDuration of the intervention, control, and wash-out periods (days), median [first quartile, third quartile]: 34.5 [30.0, 36.0]; 34.5 [30.0, 36.0]; 1.5 [1.0, 2.0]. Appendix D. Table Characteristics of patients who underwent and declined SARS-CoV-2 screening for asymptomatic/paucisymptomatic patients during the intervention period. Table D1. Characteristics of patients who underwent and declined SARS-CoV-2 screening for asymptomatic/paucisymptomatic patients during the intervention period. * Patients who accepted the rapid test but did not have a test performed were not included in this table. In univariate analysis, factors associated with performing screening for asymptomatic/paucisymptomatic patients were the following: older age, male sex, having mild symptoms, not being from France, being unemployed, being followed up for a chronic disease, not being screened previously, and self-assessment of a high risk of infec [file pmed.1004317.s001.pdf]

INTENSIFIED SCREENING FOR SARS-CoV-2 IN 18 EMERGENCY DEPARTMENTS IN THE PARIS METROPOLITAN AREA, FRANCE (DEPIST-COVID): A CLUSTER-RANDOMISED, TWO-PERIOD, CROSSOVER TRIAL

S1 File.

Appendix A. Study protocol including the initial statistical analysis plan (SAP) as submitted to French data authorities, the final SAP, a summary of the changes in the SAP, and the DEPIST-COVID questionnaire

Appendix B. Figure Geographical location of the 18 emergency departments involved in the DEPIST-COVID trial (Paris metropolitan area)

Appendix C. Table Emergency department characteristics and study period duration

Appendix D. Table Characteristics of patients who underwent and declined SARS-CoV-2 screening for asymptomatic/paucisymptomatic patients during the intervention period

Appendix E. Table Characteristics of patients tested through SARS-CoV-2 screening for asymptomatic/paucisymptomatic patients

Appendix F. Figure Newly diagnosed SARS-CoV-2-positive patients per emergency department and strategy

Appendix G. Figure SARS-CoV-2 incidence rate in the Paris metropolitan area and per geographical department (75, 77, 92, 93, 95) of the emergency departments involved in the study

Appendix H. Table Characteristics of patients newly diagnosed with SARS-CoV-2 infection

Appendix I. Primary outcome modeling, sensitivity analyses, and intercluster and intracluster correlation coefficients

Appendix J. Comparison of the proportions of new SARS-CoV-2 diagnoses through screening in emergency departments and of positive tests through community screening for individuals aged 18+ of the geographical departments of the Paris metropolitan area screened during the same period

Appendix K. Figures Visits in the 18 emergency departments during the study period in 2021 and during the same period in 2019

Appendix L. Cluster randomised trials extension of the Consolidated Standards of Reporting Trials (CONSORT) checklist

Appendix M. Study group

Appendix A. Study protocol including the initial statistical analysis plan (SAP) as submitted to French data authorities, the final SAP, a summary of the changes in the SAP, and the DEPIST-COVID questionnaire

The initial version of the "DEPIST-COVID" protocol, version 1.1 dated January 08, 2021, was approved by the French authorities and was followed by version 2.0 dated April 06, 2021, which was also approved.

The amendment in version 2.0 concerned the following 3 elements:

a) Extension of the study duration by 30 days:

   See Research schedule, page 8 of the S1 Appendix:

   “2 months (with the possibility of extending each period by a maximum of 15 days maximum), i.e. 2 months and 30 days maximum + wash-out.”

b) Modification of the ancillary study, which corresponds to a separate protocol presented separately.

c) Change in the principal investigator for one center

The study protocol provided in the S1 appendix is the version 2.0 of the document.

EVALUATION OF THE BENEFIT OF A SYSTEMATIC OFFER OF NURSE-DRIVEN SARS-CoV-2 SCREENING BY RAPID TESTING IN EMERGENCY DEPARTMENTS IN THE PARIS METROPOLITAN AREA

DEPIST-COVID TRIAL

Version No. 2-0 of 06/04/2021
Project Code: APHP201625 / ID-RCB [*Recherches et Collections Biologiques* (Biological Collections and Research)] No.: **2020-A03532-37**

Coordinating Investigator : Judith LEBLANC, RN, PhD
East Paris clinical research platform
Hospital-University Group AP-HP [*Assistance Publique–Hôpitaux de Paris* (Public Hospitals of Paris)] Sorbonne University, St Antoine Hospital
+33 01 49 28 22 02
judith.leblanc@aphp.fr

Scientific Manager : Prof. Anne-Claude CREMIEUX, PU-PH [*Professeur des universités-praticien hospitalier* (University Professor Hospital Consultant)]
Department of Infectious and Tropical Diseases
Saint Louis Hospital
+33 01 42 49 45 72
anne-claude.cremieux@aphp.fr

Sponsor : Public Hospitals of Paris (AP-HP)
and by delegation: Clinical Research and Innovation Office (*Délégation à la Recherche Clinique et à l'Innovation*, DRCI)
Saint Louis Hospital
DRCI-[Site] Project point of contact : Fatiha Aced-Djennaoui
+33 01 44 84 17 08
fatiha.djennaoui@aphp.fr

Organisation responsible for the research : Prof. Tabassome SIMON
East Paris clinical research platform (URC-Est, CRC-Est, CRB-UPMC)
Hospital-University Group AP-HP. Sorbonne University, St Antoine Hospital
+33 01 49 28 22 02
saranesrine.salhi@aphp.fr
DRCI-CRU Project point of contact: Sarah SALHI

Clinical Research and Innovation Office (DRCI)
Hôpital St. Louis, 75010 Paris

Associated teams:

- Jérôme Le Goff, PU-PH, PU-PH, Microbiology Department, Saint-Louis Hospital
- Dominique Pateron, Emergency Department, Saint Antoine Hospital
- Vittoria Colizza, INSERM [*Institut national de la santé et de la recherche médicale* (National Institute of Health and Medical Research)], Pierre Louis Institute of Epidemiology and Public Health
- Arnaud FONTANET, Tiffany CHARMET, Epidemiology Research and Expertise Unit for Emerging Diseases, Pasteur Institute
- Renaud PIARROUX, Martine PIARROUX, Parasitology - Mycology, Pitié-Salpêtrière Hospital, AP-HP
- Nathalie de Castro, Department of Infectious Diseases, St Louis Hospital.
- Flore-Anne de Baudinière, User Representative, AP-HP

This document is the property of the DRCI/APHP. Any reproduction is strictly prohibited.
Version No. 4.0 dated 31/05/2019

TABLE OF CONTENTS

1 SYNOPTIC OVERVIEW 5

2 SCIENTIFIC JUSTIFICATION OF THE RESEARCH 10

2.1 CURRENT STATE OF KNOWLEDGE WITH REGARD TO RESEARCH 10
2.2 RESEARCH HYPOTHESES 14
2.3 DESCRIPTION OF THE POPULATION TO BE STUDIED AND JUSTIFICATION OF ITS CHOICE 15
2.4 DESCRIPTION OF PROCEDURES PERFORMED OR PRODUCTS USED IN ACCORDANCE WITH THEIR CURRENT USE 15
2.5 DESCRIPTION OF THE ACTS AND PROCEDURES ADDED BY THE RESEARCH 15
2.6 SUMMARY OF THE FORESEEABLE AND KNOWN BENEFITS AND RISKS FOR RESEARCH PARTICIPANTS 16

3 RESEARCH OBJECTIVES 17

3.1 PRIMARY OBJECTIVE OF THE RESEARCH 17
3.2 SECONDARY OBJECTIVES OF THE RESEARCH 17

4 RESEARCH DESIGN 17

4.1 PRIMARY OUTCOME 17
4.2 SECONDARY ENDPOINTS 17

5 DESCRIPTION OF THE RESEARCH METHODOLOGY 18

5.1 EXPERIMENTAL DESIGN 18
5.2 NUMBER OF PARTICIPATING SITES 21
5.3 DESCRIPTION OF THE MEASURES TAKEN TO REDUCE AND AVOID BIAS 21

6 CONDUCTING THE RESEARCH 22

6.1 RESEARCH SCHEDULE 22
6.2 STUDY OUTLINE 23
6.3 PRACTICAL IMPLEMENTATION 23

7 SCREENING CRITERIA 28

7.1 INCLUSION CRITERIA 28
7.2 NON-INCLUSION CRITERIA 28
7.3 RECRUITMENT METHODS 28

8 DISCONTINUATION RULES 28

8.1 CRITERIA AND PROCEDURES FOR EARLY TERMINATION OF A SUBJECT'S PARTICIPATION IN THE RESEARCH 28
8.2 METHODS OF REPLACING THESE PERSONS, IF APPLICABLE 29

9 EFFICACY ASSESSMENT 29

2.1. DESCRIPTION OF EFFICACY ASSESSMENT PARAMETERS 29

10 VIGILANCE 29

11 SPECIFIC RESEARCH COMMITTEES: SCIENTIFIC COMMITTEE 29

12 DATA MANAGEMENT 29

12.1 DATA COLLECTION METHODS 29
12.2 IDENTIFICATION OF DATA COLLECTED DIRECTLY IN CRFS THAT WILL BE CONSIDERED SOURCE DATA 30
12.3 RIGHT OF ACCESS TO DATA AND SOURCE DOCUMENTS 30
12.4 DATA PROCESSING AND STORAGE OF DOCUMENTS AND DATA 31
12.5 DATA 31

13 STATISTICAL ASPECTS 32

13.1 STATISTICAL JUSTIFICATION FOR SAMPLE SIZE 32
13.2 DESCRIPTION OF THE STATISTICAL METHODS 32

14 ANCILLARY STUDY 34

15 QUALITY CONTROL AND ASSURANCE 34

15.1 GENERAL ORGANISATION 34
15.2 MANAGEMENT OF NON-COMPLIANCE 34
15.3 AUDIT 35

This document is the property of the DRCI/APHP. Any reproduction is strictly prohibited.

15.4 SIGNED PRINCIPAL INVESTIGATOR'S INCURRED LIABILITIES 35

16 ETHICAL AND LEGAL ASPECTS 35

16.1 INFORMATION AND CONSENT COLLECTION PROCEDURES FOR RESEARCH PARTICIPANTS 35
16.2 PROHIBITION FOR THE PERSON TO PARTICIPATE IN ANOTHER RESEARCH STUDY OR EXCLUSION PERIOD PROVIDED FOR AT THE END OF THE RESEARCH, IF APPLICABLE 35
16.3 COMPENSATION OF SUBJECTS 36
16.4 REGISTRATION IN THE NATIONAL REGISTER OF INDIVIDUALS PARTICIPATING IN RESEARCH INVOLVING HUMAN SUBJECTS......36
16.5 LEGAL OBLIGATIONS 36
16.6 REQUEST FOR OPINION FROM THE ETHICS COMMITTEE EC 36
16.7 INFORMATION FROM THE ANSM [*AGENCE NATIONALE DE SECURITE DU MEDICAMENT ET DES PRODUITS DE SANTE* (FRENCH NATIONAL AGENCY FOR MEDICINES AND HEALTH PRODUCTS SAFETY)]......36
16.8 PROCEDURES RELATING TO IT REGULATIONS AND ITS FREEDOMS 36
16.9 AMENDMENTS TO THE RESEARCH 36
16.10 FINAL RESEARCH REPORT 37
16.11 ARCHIVING 37

17 FINANCING AND INSURANCE 37

17.1 SOURCE OF FUNDING 37
17.2 INSURANCE 37

18 RULES REGARDING PUBLICATION 37

18.1 MENTION OF THE AP-HP AFFILIATION FOR THE PROJECTS SPONSORED BY THE AP-HP......38
18.2 MENTION OF THE SPONSOR THE AP-HP (DRCI) IN THE "ACKNOWLEDGEMENTS" OF THE MANUSCRIPT38
18.3 MENTION OF THE FUNDING BODY IN THE "ACKNOWLEDGEMENTS" OF THE MANUSCRIPT 38

19 BIBLIOGRAPHY 39

20 LIST OF ADDENDA 42

This document is the property of the DRCI/APHP. Any reproduction is strictly prohibited.
Version No. 4.0 dated 31/05/2019

1 SYNOPTIC OVERVIEW

Full title	Evaluation of the Benefit of a Systematic Offer of Nurse-Driven SARS-CoV-2 Screening by Rapid Testing in Emergency Departments in the Paris Metropolitan Area
Acronym/reference	DEPIST-COVID trial
Principal Investigator	Judith LEBLANC
Scientific Manager	Prof. Anne-Claude CREMIEUX
Sponsor	Assistance Publique – Hôpitaux de Paris
Scientific Rationale	European countries today face another wave of the SARS-CoV-2 pandemic, which has led to a second lockdown in France in November 2020 in order to avoid overwhelming health services. To prevent or reduce a third wave, the strategy calls for vaccination, maintaining barrier measures and testing and isolating infected persons in order to break the cycles of infection. The latter objective is made difficult by the existence of asymptomatic carriers or paucisymptomatic carriers that have very few symptoms and that aren't tested. Identification of these carriers in the general population is usually based on a search for close contact persons from those who were tested positive or from identified clusters. Experiments of mass testing are being carried out in Liverpool or Slovakia but, in order for them to be effective, they must be repeated, which limits feasibility. Another strategy of wide screening in the general population to identify asymptomatic persons is to offer a systematic screening during medical consultations and particularly in the emergency departments (ED). This strategy grants access to the entire population attending health facilities, including persons with lower income. This strategy can be conducted continuously in order to: 1) contribute to controlling the epidemic by identifying and isolating asymptomatic persons and their close contacts; 2) provide an observation on the evolution of viral circulation in the general population. This strategy has, to our knowledge, not yet been evaluated and we propose to implement it in 18 emergency departments in the Paris Metropolitan area, one of the regions most affected by SARS-CoV-2.
Objective and main evaluation criteria	Evaluate the benefit of a systematic offer of SARS-CoV-2 screening by rapid testing (molecular multiplex PCR/RT-LAMP) to identify infected persons, associated with the usual practice of the EDs and compared to a period based on usual practice of the EDs.<br>**Primary outcome**: proportion of patients tested positive for SARS-CoV-2 amongst the flow of consulting adults.
Objectives and secondary evaluation criteria	– Compare the proportion of patients who have tested positive for SARS-CoV-2 amongst asymptomatic patients tested in the Paris Metropolitan area in the same period (the comparison will also be done

This document is the property of the DRCI/APHP. Any reproduction is strictly prohibited.
Version No. 4.0 dated 31/05/2019

	amongst the geographical area of each ED, amongst symptomatic patients tested and also amongst the entire tested population),<br>- Evaluate the feasibility of the screening strategy,<br>- Describe the patients tested and positive for SARS-CoV-2 in both periods:<br>o Describe the proportion of positive tests,<br>o Describe the proportion of asymptomatic patients among tested patients and among patients tested positive for SARS-CoV-2,<br>o For the symptomatic patients tested positive: describe the symptomatology,<br>o Describe the patient exposure factors according to the SARS-CoV-2 test result with the help of the DEPIST-COVID questionnaire,<br>- Describe sociodemographic and behavioural factors and habits associated with SARS-CoV-2 infection in patients tested in one of the following three schemes: DEPIST-COVID, ComCor and COVISAN,<br>- Estimate the incidence of new SARS-CoV-2 infections in the Paris Metropolitan area: proportion of positive patients and their profiles. Estimate the under-detection of cases in the region, and compare with results from the model experiment,<br>- Estimate the number of close contact persons who have been tested for SARS-CoV-2 and who have isolated over both periods.<br><br>The secondary evaluation criteria are:<br>- To compare the proportion of patients who have tested positive for SARS-CoV-2 among asymptomatic patients tested, and proportion in the Paris Metropolitan area in the same period and the comparison will also be made within the geographical areas of each ED (the proportion of patients who have tested positive for SARS-CoV-2 among symptomatic patients tested and within the entire population tested will also be noted in the study, as well as within the Paris Metropolitan region and within the geographical areas of each ED),<br>- Proportion of patients who have completed the questionnaire, proportion of offered, accepted and performed tests, and notified of the SARS-CoV-2 result and staff involved (nurses, caregivers, doctors, others),<br>- Proportion of patients who have tested positive for SARS-CoV-2 in both strategies (positive tests),<br>- Proportion of asymptomatic patients among the tested patients and among the tested patients positive in both strategies,<br>For the symptomatic patients tested positive in both strategies and in the total population: symptomatology,

This document is the property of the DRCI/APHP. Any reproduction is strictly prohibited.
Version No. 4.0 dated 31/05/2019

	Patient exposure factors in the two study strategies and according to the result of the SARS-CoV-2 test using the items in the DEPIST-COVID questionnaire,<br>- Sociodemographic, behavioural factors and habits associated with SARS-CoV-2 infection in patients tested in the following three schemes: DEPIST-COVID (positive cases, negative controls), ComCor (cases, controls) and COVISAN,<br>- Estimate of incidence in the region, estimate of under-detection of cases in the region, and comparison with results from the model experiment,<br>- Number of close contact persons who have been tested, positive for SARS-CoV-2 and isolated over both periods.
Experimental scheme	Multicentre, randomised, cluster and cross-over trial in two periods comparing a period with usual practice of emergency services (control strategy) with a period with a systematic SARS-CoV-2 testing offered associated with usual practice (intervention strategy).
Population concerned	All consulting adults (without upper age limit) visiting a participating department
Inclusion criteria	All consulting adults (without upper age limit) visiting a participating department
Non-inclusion criteria	N/A
Procedures or Product that are being researched	Systematic offer of rapid, nurse-driven SARS-CoV-2 screening (molecular multiplex or RT-LAMP) combined with usual practice
Control group	Usual diagnostic practices of SARS-CoV-2 in emergency departments
Other acts or procedures added by research	N/A
Expected benefits for participants and for society	The intervention strategy would allow:<br>- screening for rare or asymptomatic forms of SARS-CoV-2 infection that escape the syndromic diagnosis and thus contribute to the reduction of the virus's active cycles of infection,<br>- monitoring the community circulation of the SARS-CoV-2 virus in the Paris Metropolitan area on a sample representative of the general population of the Paris Metropolitan area and not on voluntary screening,<br>- estimating, by modelling, the incidence of new SARS-CoV-2 infections in the Paris Metropolitan area during the study period,<br>- monitoring the circulation of other respiratory viruses among symptomatic individuals.
Minimal risks and constraints added by the research	Nasopharyngeal sampling for SARS-CoV-2 diagnosis
Practical implementation	**Intervention strategy**

This document is the property of the DRCI/APHP. Any reproduction is strictly prohibited.
Version No. 4.0 dated 31/05/2019

	A self-administered questionnaire on SARS-CoV-2 symptoms, possible contact, exposure risk situations, as well as socio-demographic characteristics will be offered to patients enrolled who are able to complete it.<br>If necessary, the nurse will help the patient complete the questionnaire.<br>In the triage room or in a cubicle, SARS-CoV-2 screening will be offered by the nurse regardless of the answer to the questionnaire.<br>A nasopharyngeal sample will be taken by the nurse in accordance with the personal protection measures recommended in the hospital protocol, with the exception of patients who cannot have a nasopharyngeal sample (contraindication of thrombocytopenia or ENT malformation leading to an oropharyngeal sample being offered). Trained caregivers will also be authorised to carry it out under the responsibility of a physician or registered nurse at their institution.<br>The choice of test will be determined based on the presence of suggestive symptoms.<br>For patients with symptoms suggestive of SARS-CoV-2 infection, known as “symptomatic”: a multiplex PCR test will be performed with result in 75 minutes.<br>For patients with no symptoms suggestive of SARS-CoV-2 infection, known as “asymptomatic”: an RT-LAMP test will be performed.<br>In the event of a positive test, the patient will be treated according to the circuit defined by each hospital. Outpatients will be referred to the usual department for follow-up and contact tracing.<br>In the event of a negative test, the usual emergency procedure will follow.<br>A subsequent telephone follow-up of patients will be performed to retrieve their profile.<br><br>**Control strategy**<br>The doctor offers a SARS-CoV-2 test according to current recommendations. Patients with a positive test will be asked to complete the questionnaire. Patients who have not completed it will be contacted by telephone to answer the DEPIST-COVID questionnaire by telephone.
Number of subjects screened	104,000 consulting adults
Number of sites	18 sites in the Paris Metropolitan area
Research schedule	Duration of inclusion: 2 months (with the possibility of extending each period by a maximum of 15 days): 2 months and 30 days maximum + wash-out.<br>- Duration of participation (treatment + follow-up): 1 day (and up to 15 days ± 10 if monitored)<br>- Total duration: 2 months (and 30 days maximum + wash-out) + patient follow-up over a period of 15 days ± 10

This document is the property of the DRCI/APHP. Any reproduction is strictly prohibited.
Version No. 4.0 dated 31/05/2019

Number of inclusions expected per centre and per month	2889
Statistical analysis	There is no interim analysis planned. The analysis will be performed at the end of the research after the data review meeting and freezing of the database, according to the intention-to-treat principle and taking into account cluster randomisation
Source of funding	ANRS [Agence Nationale de la Recherche (National Research Agency)] – REACTing and the AP-HP

This document is the property of the DRCI/APHP. Any reproduction is strictly prohibited.
Version No. 4.0 dated 31/05/2019

2 SCIENTIFIC JUSTIFICATION OF THE RESEARCH

2.1 CURRENT STATE OF KNOWLEDGE WITH REGARD TO RESEARCH

2.1.1 Status of the epidemic and of control measures

As of 25 November, 2020, the SARS-CoV-2 pandemic is responsible for more than **1,400,000 deaths and nearly 60 million confirmed cases worldwide** [11]. Beyond its health impact, its economic impact is also considerable. To contain the first wave while avoiding a massive overflow of hospitals, most countries had to impose a generalised lockdown on their population such as in France, or localised to the most affected regions such as in China. However, some countries, such as South Korea, have succeeded in controlling the epidemic without strict population confinement, or have limited the health impact, such as Germany or China. These countries are particularly characterised by **extensive use of screening tests** to identify infected persons [2-4].

At the end of the first lockdown, some countries, mostly Asian, such as China, South Korea, Singapore, Hong Kong and Taiwan, countries marked by the SARS crisis, but also New Zealand, relied on a very reactive testing-trace-isolate system associated with the installation of sanitary cordon to control emerging epidemic outbreaks and cycles of infection. To date, some of these countries seem to have avoided the occurrence of a second wave.

European countries are today facing a second wave that has imposed a new curfew and/or lockdown in November 2020 to prevent health systems from being overwhelmed [5].

It is important today to learn from the past few months to avoid a new epidemic rebound when the second lockdown ends.

Taking these experiences into account, in order to control the spread of SARS-CoV-2, the strategy of exiting of lockdown in France must be based on two pillars [6, 7]:
- maintaining a certain level of individual barrier measures such as social distancing, wearing masks and hand hygiene, and also collective measures, such as the ban on gatherings and the closure of places of activities conducive to viral circulation [8].
- the screening and isolation of infected persons and their contacts to break the cycles of infection [9].

This last objective is made very difficult by the existence of **asymptomatic or paucisymptomatic carriers who do not consult and are not tested** and therefore do not know that they are carriers of the virus [9]. It is estimated that more than a quarter of people infected with SARS-CoV-2 remain completely asymptomatic throughout the infection [10]. Asymptomatic infections are seen in all age groups, including the elderly, as several studies on EHPADs [*Établissement d'hébergement pour personnes âgées dépendantes* (Nursing homes)] have shown since March 2020 [11]. The amount of virus isolated in asymptomatic forms is identical to that of symptomatic forms [12] and the contagiousness of these asymptomatic forms is now well established [13]. They are considered to be the main explanation for the **invisible spread of the virus** in the first quarter of 2020 in most countries and the **major reason for the difficulty in controlling this pandemic.**

It should also be remembered that 50% of transmissions occur during the pre-symptomatic phase of the source patient [10] which begins 48 hours before the onset of symptoms [14] which greatly complicates the control of transmission chains and explains the importance of quickly identifying carriers of the virus.

This document is the property of the DRCI/APHP. Any reproduction is strictly prohibited.
Version No. 4.0 dated 31/05/2019

This means **the importance of quickly identifying these asymptomatic or presymptomatic people before they transmit their infection**. Any delay between the onset of symptoms and isolation decreases the chances of controlling the epidemic [15].

2.1.2 The screening strategies currently implemented [16]

With the increase in the availability of tests, most countries have first sought to encourage **people with symptoms** to be tested by increasing the number of test sites, including in temporary locations (screening booths, lorries, marquees, etc.) and, in France, in particular by making tests accessible without a prescription and free of charge [16].

The identification of asymptomatic or paucisymptomatic infected people from the general population who will not consult initially relied on the search for contact cases from persons tested positive and large screening campaigns around identified clusters.

In different European countries, **systematic screening** is also offered to **certain particularly exposed populations** (hospital nursing staff, EHPAD teams and residents, for example), likely to be the cause of major clusters (screening before hospitalisation for surgery or delivery, for example) or in **communities of young adults** (university campus, for example) where the virus is particularly fast and silent due to the high level of asymptomatic forms. Modelling encourages these screening approaches [17, 18].

Other methods may be offered to increase the effectiveness of the screening:

Mass screening of the entire population of a country was proposed [19]. Iceland has tested 15% of its population and seems to have controlled the spread of the virus to date [20, 21]. Experiments are currently underway in certain cities such as Liverpool [22] or on a nation-wide scale such as Slovakia where two-thirds of the population have been tested [23]. However, to be effective, these mass screenings should be repeated regularly, which limits their feasibility.
In addition, the first results of the Liverpool experiment show that people from underprivileged neighbourhoods are three times less inclined to be tested for than those from more affluent neighbourhoods, even though they are more at the risk of SARS-CoV-2 infection [8, 24, 25].

Another broad screening strategy in the general public to identify asymptomatic people is to **offer systematic screening during any medical consultation**, as has long been practiced to fight the spread of infectious diseases that can remain asymptomatic, such as syphilis [26] or, more recently, to screen people who do not know about their HIV infection, first of all by the ACDC [27] and then in France [28].

According to our literature review, this strategy has not yet been evaluated in the context of SARS-CoV-2, probably because most countries have, until recently, had little real screening capacity.

Unlike the voluntary mass screening approach mentioned above, this strategy has the advantage that it can access the entire population visiting the healthcare facilities, including those with a lower socioeconomic status, and can be conducted continuously and thus:

1- contribute to the control of the epidemic by identifying and isolating asymptomatic people and their contacts, but also,

2- provide an observation on the evolution of viral circulation in the general population.

We propose to evaluate the systematic offer of SARS-CoV-2 **screening** in the emergency departments of the Paris Metropolitan area, as we did to assess the public health impact of the

This document is the property of the DRCI/APHP. Any reproduction is strictly prohibited.
Version No. 4.0 dated 31/05/2019

systematic offer of HIV screening [29]. This research will be **conducted prior to the ANRS DEPIST 14055 (HIV) study, which was being launched** in 18 emergency departments in the Paris Metropolitan area during the summer of 2020. The DEPIST-COVID research could therefore be carried out in a very short space of time, compatible with conducting a study on this rapidly evolving pandemic.

2.1.3 Rationale for a study on the proposal of systematic screening of SARS-CoV-2 in the emergency departments of the Paris Metropolitan area

Every year, the hospital emergency departments welcome 20 million patients [30], i.e. a volume equivalent to approximately 30% compared to the general population, including in the Paris Metropolitan area [31]. This high attendance makes it a favourable observation to assess the relevance of the systematic offer of screening to the general population.

It is also one of the only places that is frequented by **all segments of the population, including people with lower resources. These populations are more difficult to reach and are underrepresented** in mass screening campaigns in the general population, as shown by the recent experiment in Liverpool [23].

In a previous study on the systematic offer of HIV testing, the characteristics of the emergency department population (n=12,000) were compared to those of the general population and reflected the latter with a slight over-representation of people born in Sub-Saharan Africa [29].

This highlights the value of offering testing at the emergency department to identify asymptomatic infections among populations that may be at higher risk of SARS-CoV-2 infection and not be captured by other current screening schemes.

The Paris Metropolitan area region with a high population density was **one of the regions most affected by the SARS-CoV-2 epidemic** during the first wave in February-May 2020 and the remains so in the second wave from September 2020 to today [32]. However, the immunity of the population is undoubtedly quite far from having reached levels allowing it to be protected from another epidemic phase.

This second wave is occurring in a context of circulation of other respiratory viruses.

Hospital emergencies are a **particularly appropriate to offer systematic screening for SARS-CoV-2 infection** in any person with or without respiratory symptoms regardless of their reason for consultation. They have the advantage of having qualified staff being able to take samples and perform tests quickly and an environment that facilitates the rapid and easy organisation of tracing and isolation.

Following the example of other screening procedures assessed in these same departments by our team, these tests could be offered and performed by the nursing teams and the result would be available in less than 2 hours [29, 33], the median duration of a consultation in the emergency room being 3 hours [31].
During the period of seasonal viral epidemics, it is important that the rapid PCR test used in people with symptoms suggestive of respiratory infection can **also** detect other respiratory viruses (*influenzae* virus, respiratory syncytial virus, parainfluenza virus, seasonal coronavirus, rhinovirus). In people who do not have these symptoms, a rapid RT-LAMP test to detect only SARS-CoV-2 will be used.

There are many benefits to a systematic offering of screening for SARS-CoV-2 by rapid test in the emergency department. It would allow:

This document is the property of the DRCI/APHP. Any reproduction is strictly prohibited.
Version No. 4.0 dated 31/05/2019

1- to test for pauci or asymptomatic forms of SARS-CoV-2 infection that can escape other screening schemes, [9] and thus contribute to the reduction of the active cycles of infection,

2- to quickly confirm or refute the Covid-19 diagnosis in people with respiratory symptoms (syndromic diagnosis),

3- to limit the risks of transmission of persons tested positive thanks to the immediate and direct activation of the COVISAN scheme to support the isolation of persons who are not hospitalised (delivery of isolation measures/reduction of transmission risks), the search for contact cases by home testing of household members and the search for the source by questionnaire (a collaboration is established with Prof. Renaud Piarroux and the COVISAN team),

4- to analyse the exposure factors in persons tested positive using the DEPIST-COVID questionnaire, as well as to accompany participation in the ComCor study of the Pasteur Institute (paper questionnaire (see addendum 6) or online on the study site where possible) of the Pasteur Institute to assess the sociodemographic, behavioural factors and practices associated with SARS-CoV-2 infection (a collaboration is established with Prof. Arnaud Fontanet and the ComCor study team) to compare the characteristics of patients tested positive in three schemes: DEPIST-COVID, ComCor and COVISAN,

5- to monitor the community circulation of the SARS-CoV-2 virus in the Paris Metropolitan area on a sample of individuals consulting the emergency departments and not on biased voluntary screening, as well as to estimate by modelling the incidence of new SARS-CoV-2 infections (collaboration has been established with Prof. Vittoria Colizza and the ComCor study team) and the public health benefit of the screening process.

2.1.4 Context: Situation of the subject and involvement of the teams on the subject

2.1.4.1 Subject's situation

According to our literature review, there is currently no study evaluating the value of systematically offering screening to the general population in healthcare facilities (and particularly in the emergency departments) to identify people who are unaware of their SARS-CoV-2 infection and to better assess the prevalence of undiagnosed infections and therefore viral circulation. However, this strategy has been used for a long time, in addition to voluntary testing and active contact research, to fight the spread of infectious diseases that may remain asymptomatic, such as syphilis or HIV infection.

The originality of the proposed study is to adopt a comparative cluster-cross-over methodology per period (see below) to precisely document the benefit of the screening strategy being studied by aiming to reach a threshold of new cases. The threshold was estimated when calculating the sample size (see below). This involves quantifying, using a robust methodology, the benefit of the screening strategy for a population with and without symptoms.

The populations received vary from one emergency department to another, as do SARS-CoV-2 screening practices. Departments perform rapid PCRs for symptomatic patients; others do not have an offsite laboratory. The investigational design of the crossover cluster study will take into account this heterogeneity of the partaking persons and the practices by allowing each participating department to carry out the two evaluation phases. This design will make it possible to highlight the benefit of an intensification of testing at the 18 sites.

The results will contribute to reflection on the relevance of screening strategies and will encourage the rapid implementation of the strategy and its replication if it is deemed conclusive.

This document is the property of the DRCI/APHP. Any reproduction is strictly prohibited.
Version No. 4.0 dated 31/05/2019

2.1.4.2 Involvement of the teams

Our team has been working for a long time on testing in the emergency departments of the Paris Metropolitan area. Our previous study on routine testing in the emergency departments of the Paris Metropolitan area using rapid HIV tests performed by the nursing team in 2009-2010, funded by the ANRS and SIDACTION, showed that people who were unaware of their HIV seropositivity belonged almost all to the populations most exposed to HIV [29]. This work also allowed us to assess the prevalence of undiagnosed infections in the Paris Metropolitan area in the emergency department population, an assessment confirmed by other teams [34]. Following this study, we conducted a second study at the Paris Metropolitan area [33] emergency departments and showed that targeted screening for exposed populations was effective in identifying patients who were not aware of their seropositivity.

A new research project evaluating the implementation of this screening procedure by a pragmatic intervention trial has received the approval of the ANRS (DEPIST ANRS 14055). It was being set up in 18 emergency departments in the Paris Metropolitan area in September 2020. There is therefore a unique opportunity to assess, in very short and compatible times with the necessary reactivity of the public health intervention research during a health crisis linked to new infectious agents of an unpredictable nature, the strategy for the systematic offer of SARS-CoV-2 screening in the Paris Metropolitan area emergency departments. The DEPIST-COVID study will be conducted prior to the DEPIST study, the start of the enrolment period of which is postponed until after the completion of DEPIST-COVID. This study will therefore benefit from the entire implementation phase of the DEPIST study already conducted, which will make its start-up much faster.

The systematic offer for screening and non-targeted consultants is logical in the face of a respiratory infection affecting the entire adult population. It should be noted that several studies show that children under 10 years of age may be less sensitive to infection than adolescents and adults [20, 35], while adults over 60 years of age appear more sensitive.

This work is based on a team that is motivated to use its expertise in this field to improve knowledge and control of the SARS-CoV-2 pandemic. It also benefits from the support of UHF IMPEC, which brings together all the emergency services of the AP-HP and whose work-package on paramedical research is coordinated by J. Leblanc.

It is perfectly integrated into the ANRS-REACTing missions, whose field of activity is expanding to new infectious risks and which has been developing public health intervention and prevention research as well as research in nursing sciences for several years.

2.2 Research hypotheses

Our working hypotheses are that the systematic offering of SARS-CoV-2 screening by rapid emergency tests performed by nursing teams, combined with current diagnostic practice in patients with signs suggestive of Covid-19, would be superior to the control strategy and would allow:

- testing for rare or asymptomatic forms of SARS-CoV-2 infection, particularly in people who may escape other screening schemes, and thus improve the control of SARS-CoV-2 circulation in the Paris Metropolitan area, and compare the positivity rate among asymptomatic patients in the study to that of the Paris Metropolitan area region, while expecting to obtain a higher rate in the emergency department population,

This document is the property of the DRCI/APHP. Any reproduction is strictly prohibited.
Version No. 4.0 dated 31/05/2019

- to study the sociodemographic, behavioural factors and practices associated with SARS-CoV-2 infection in the population tested in the emergency department compared to those captured by other schemes (ComCor and COVISAN),

- estimating, by modelling, the incidence of new SARS-CoV-2 infections in the Paris Metropolitan area during the study period,

- to limit the risks of transmission of persons tested positive through the immediate and direct activation of the COVISAN scheme facilitating the steps of isolation, tracing, and source search.

2.3 Description of the population to be studied and justification of its choice

The Paris Metropolitan area is a region of metropolitan France with a high prevalence of SARS-CoV-2. According to the SIVIC data of 26 November 2020, the incidence rate in the Paris Metropolitan area is 115 per 100,000 inhabitants compared to 151 per 100,000 at the national level. There are departmental variations with an incidence in Paris of 90, in Seine Saint Denis at 142, the Val et Marne at 153, the Hauts de Seine at 108.

Of the 18 DEPIST study sites, 17 sites agreed to participate in the DEPIST-COVID study (see addendum 1). According to the data collected from the department heads during the week of 16 November 2020, emergency departments currently use PCR or RT-LAMP tests mainly for diagnosis purposes for patients with symptoms suggestive of SARS-CoV-2 or as screening for patients who need to be hospitalised for another reason before being transferred to the hospital department (to avoid hospital clusters), an average of 30 tests per day per department. The sites do not report using antigen testing.

During the previous 2 research projects, effective collaboration had been established with these sites, identified on their recruitment capacities and motivation.

2.4 Description of procedures performed or products used in accordance with their current use

In the context of the current pandemic, the health authorities recommend increasing the offer of SARS-CoV-2 testing. Thus, a decree of 24/07/2020 authorises nurses to perform, without medical prescription, nasopharyngeal sampling procedures for the screening of SARS-CoV-2 infection; trained caregivers are also authorised to do so under the responsibility of a physician or qualified nurse from their institution.

2.5 Description of the acts and procedures added by the research

The start of the study evaluation periods (i.e. control strategy period and intervention strategy period during which SARS-CoV-2 screening will be offered by nurses) will be randomly assigned for each site following a “cluster and crossover per period” randomisation.

During the intervention strategy period, the proposal of a self-administered questionnaire will be the other procedure added by the research (see addendum 2). The questionnaire concerns SARS-CoV-2 symptoms, contagion possibilities and situations at risk of exposure, as well as sociodemographic characteristics. It was developed by the research team from other questionnaires currently used in France (SocialCov and ComCor questionnaires, Pasteur Institute) or elsewhere [36].

Regardless of the answers to the questionnaire, SARS-CoV-2 screening by nasopharyngeal sampling will be offered by the triage nurse (*infirmier organisateur de l’accueil*, IOA) or the nurse in the cubicle.

This document is the property of the DRCI/APHP. Any reproduction is strictly prohibited.
Version No. 4.0 dated 31/05/2019

A multiplex PCR test for symptomatic patients or an RT-LAMP test for asymptomatic patients will be performed, according to the symptomatology reported in the self-questionnaire.

During this period, the doctor may also offer a test to the patient if he/she has not already been offered by the nurse.

After performing a test, the patient will be treated according to the circuit defined in each hospital.

In the event of a positive test, if the patient is hospitalised, his/her care will be carried out according to the circuit defined by each hospital. Non-hospitalised patients will be referred to COVISAN or the usual department for follow-up and contact tracing. In the event of a negative test, the usual emergency procedure will follow.

For patients with a positive SARS-CoV-2 test and who have not completed the DEPIST-COVID self-administered questionnaire, a subsequent telephone follow-up will be performed to retrieve their profile based on the questions in the self-administered questionnaire. Patients will be accompanied by the research staff to complete the ComCor questionnaire of the Pasteur Institute to facilitate the study of sociodemographic, behavioural factors and practices associated with SARS-CoV-2 infection.

2.6 Summary of the foreseeable and known benefits and risks for research participants

The expected adverse events during the SARS-CoV-2 screening practice from a nasopharyngeal sample are:

1. the appearance of discomfort or cough when the nasopharyngeal sample is taken,
2. the need to redo the test if it is invalid or read outside the time limits or if it must be confirmed.

Risks for people can be considered minimal.

Nurse screening for SARS-CoV-2 could become a common practice during the pandemic period in emergency services if it is shown that its implementation makes it possible:
1- to screen for the pauci or asymptomatic forms of SARS-CoV-2 infection that can escape other screening schemes, and thus contribute to the reduction of the active cycles of infection,

2- to quickly confirm or refute the Covid-19 diagnosis in people with respiratory symptoms (syndromic diagnosis),

3- to limit the risks of transmission of persons tested positive through the immediate and direct activation of the COVISAN scheme to support the isolation of persons who are not hospitalised (conveying isolation measures/reduction of transmission risks), the search for contact cases by home testing of household members and the search for the source by questionnaire,

4- to analyse the exposure factors in persons tested positive using the DEPIST-COVID questionnaire, as well as to accompany participation in the ComCor study of the Pasteur Institute to evaluate the sociodemographic, behavioural factors and practices associated with SARS-CoV-2 infection and to compare the characteristics of positive patients in three schemes: DEPIST-COVID, ComCor and COVISAN,

5- to monitor the community spread of the SARS-CoV-2 virus in the Paris Metropolitan area on a sample of individuals consulting in emergency departments and not on biased voluntary testing, as well as to estimate by modelling the incidence of new SARS-CoV-2 infections and the public health benefit of the testing process.

This document is the property of the DRCI/APHP. Any reproduction is strictly prohibited.
Version No. 4.0 dated 31/05/2019

3 RESEARCH OBJECTIVES

3.1 Primary objective of the research

The primary objective is to evaluate the benefit of the systematic offer of a rapid molecular multiplex PCR (RT-LAMP) SARS-CoV-2 screening test to identify infected people in combination with current emergency services practice compared to a control period based on current emergency services practice.

3.2 Secondary objectives of the research

The secondary objectives are:

- To compare the proportion of SARS-CoV-2-positive patients among asymptomatic patients tested to that of the Paris Metropolitan area during the same period (the comparison will also be made by geographical area of each emergency department, and among the symptomatic patients tested and among the entire tested population),
- Evaluate the feasibility of the screening strategy,
- Describe patients tested and positive for SARS-CoV-2 in both periods:
    - Describe the proportion of positive tests,
    - Describe the proportion of asymptomatic patients among tested patients and among patients tested positive for SARS-CoV-2,
    - For the symptomatic patients tested positive: describe the symptomatology,
    - Describe the patient exposure factors according to the SARS-CoV-2 test result with the help of the DEPIST-COVID questionnaire,
- Describe sociodemographic and behavioural factors and habits associated with SARS-CoV-2 infection in patients tested in one of the following three schemes: DEPIST-COVID (positive cases and negative controls), ComCor and COVISAN,
- Estimate the incidence of new SARS-CoV-2 infections in the Paris Metropolitan area: proportion of positive patients and their profiles. Estimate the under-detection of cases in the region, and compare with results from the model experiment,
- Estimate the number of close contact persons who have been tested for SARS-CoV-2 and who have isolated over both periods.

An ancillary study will focus on the evaluation of rapid SARS-CoV-2 detection techniques in asymptomatic patients, in particular rapid antigen testing (RAT) and rapid molecular testing (RT-LAMP), compared to a reference technique, RT-PCR (see § 10. Ancillary study).

4 RESEARCH DESIGN

4.1 Primary outcome

The primary outcome is: the proportion of patients tested positive for SARS-CoV-2 amongst the flow of consulting adults.

It is important to note that the denominator is a number of patients in the emergency departments and not the number of tests performed.

4.2 Secondary endpoints

This document is the property of the DRCI/APHP. Any reproduction is strictly prohibited.
Version No. 4.0 dated 31/05/2019

The secondary evaluation criteria are:

1. Compare the proportion of patients tested positive for SARS-CoV-2 amongst asymptomatic patients tested in the Paris Metropolitan area in the same period, as well as in the geographical area of each ED,
   (the proportion of patients positive for SARS-CoV-2 among the symptomatic patients tested and among the entire population tested will also be noted in the study, as well as within the Paris Metropolitan area and in each geographical area of the EDs),

2. Proportion of patients who have completed the questionnaire, proportion of offered, accepted and performed tests, and notified of the SARS-CoV-2 result and staff involved (nurses, caregivers, doctors, others),

3. Proportion of patients who have tested positive for SARS-CoV-2 in both strategies (positive tests),
   Proportion of asymptomatic patients among the patients tested and among the patients tested positive in the 2 strategies,
   For symptomatic patients who have tested positive in the 2 strategies and the total population: description of the symptomatology,
   Patient exposure factors in the two study strategies and according to the result of the SARS-CoV-2 test using the items in the DEPIST-COVID questionnaire,

4. Sociodemographic, behavioural factors, and practices associated with SARS-CoV-2 infection in patients tested in 3 schemes: DEPIST-COVID (positive cases and negative controls matched on age, sex and area of residence in the last 10 days), ComCor and COVISAN,

5. Estimate of incidence in the region, estimate of under-detection of cases in the region, and comparison with results from the model experiment,

6. Number of close contact persons who have been tested, positive for SARS-CoV-2 and isolated over both periods.

An ancillary study will focus on the evaluation of rapid techniques for detecting SARS-CoV2 in asymptomatic and symptomatic patients consulting in emergency departments participating in the DEPIST-COVID trial by comparing the performance of rapid antigen tests (TRA) on saliva self-sampling compared to the reference technique RT-PCR on saliva self-sampling. The ancillary study will be carried out during the intervention strategy period of the DEPIST-COVID trial and/or during an extension of the study inclusion period.
This ancillary study is the subject of a protocol appended to the DEPIST-COVID study, currently being validated by REACTING/CAPNET.

5 DESCRIPTION OF THE RESEARCH METHODOLOGY

5.1 Experimental Design

This interventional, prospective, multicentre study (n=18) is a two period, randomised, cluster and cross-over trial comparing:
- A period with a systematic offer of SARS-CoV-2 testing combined with the usual practice of emergency services (intervention strategy),
- A period based on usual practice (control strategy).

This document is the property of the DRCI/APHP. Any reproduction is strictly prohibited.
Version No. 4.0 dated 31/05/2019

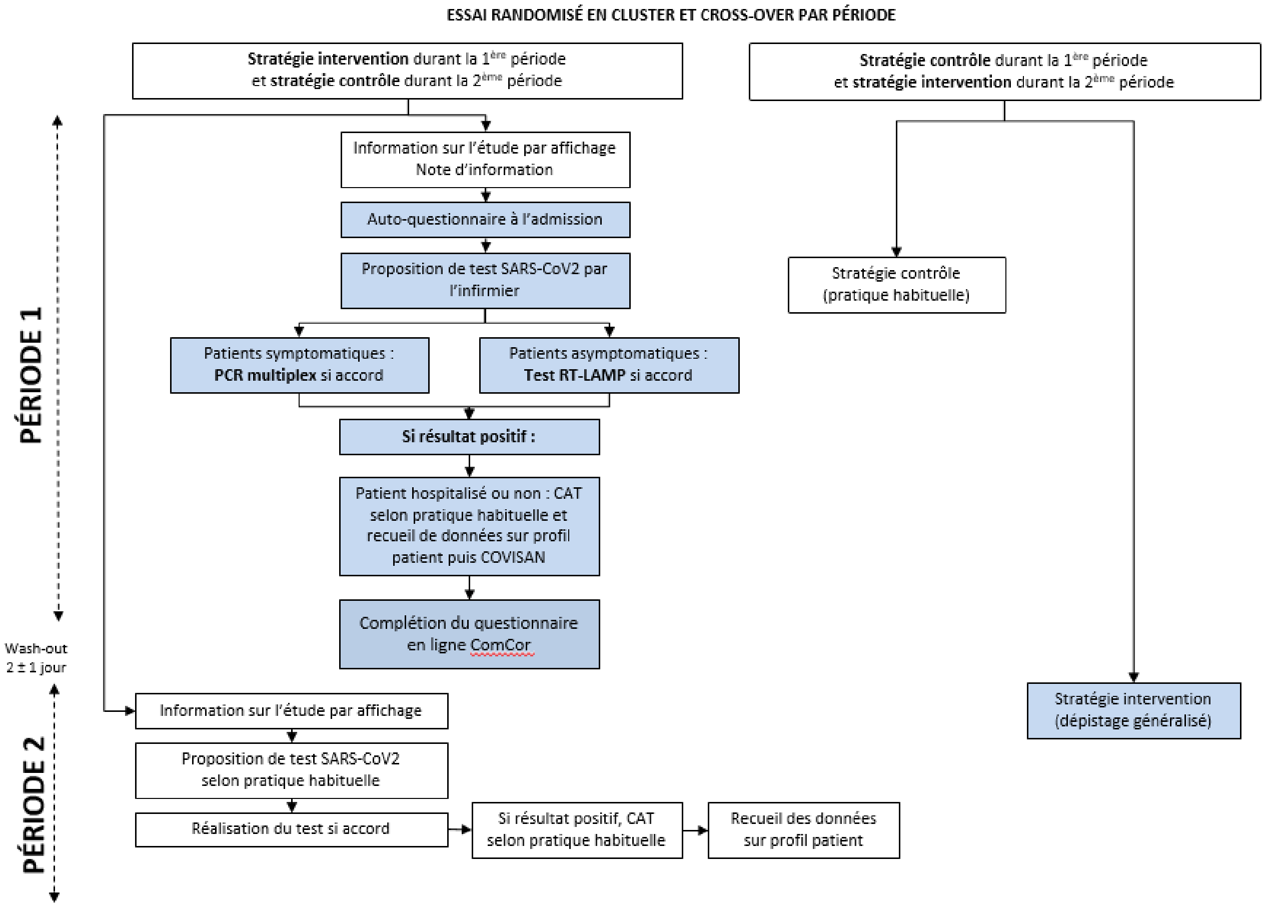

Individual randomisation cannot be considered for reasons of organisation, feasibility, and generalisability since it would require intermittent involvement by nurses for testing. The "cluster and period cross-over" method limits biases compared to a before-and-after type study.

Randomisation will determine the strategy to be applied per site to the first intervention period: either period 1 including a systematic offer of SARS-CoV-2 screening then period 2 with usual practice alone, or period 1 with usual practice alone then period 2 including the systematic offer of SARS-CoV-2 screening.

During the control period, the doctor will offer a SARS-CoV-2 screening test according to usual recommendations. At the time of writing of this project, the tests prescribed for patients with signs suggestive of SARS-CoV-2 infection are PCR or RT-LAMP tests.

During the intervention period, a self-administered questionnaire is offered to all patients over 18 years of age who are able to complete it (patients with a life-threatening emergency, with an altered state of consciousness, severe psychiatric disorders, a language barrier (oral) or deprived of liberty will not be included). This questionnaire will be available in paper format and may be completed by the patient alone or with the help of the nurse if necessary.

The testing is offered by the triage nurse (IOA) treating the patient upon arrival in the department or the nurse setting up the patient in the consultation cubicle. A multiplex PCR test is performed for patients with symptoms suggestive of respiratory infection or a simple RT-LAMP test for asymptomatic patients. During this period, the doctor may also offer a test to the patient if he/she has not already been offered by the nurse.

The duration of each period will be one month (with the possibility of extending the period to a maximum of 1 month and 15 days).

This document is the property of the DRCI/APHP. Any reproduction is strictly prohibited.
Version No. 4.0 dated 31/05/2019

For centers with an extension of the inclusion period to 1 month and 15 days, the second period from the same center will have a similar inclusion duration.

The number of patients enrolled per site and per period will vary. Under the current circumstances, a short "wash-out" period of at least 1 day is planned, allowing the removal of the equipment necessary for the intervention for the centers having started with the intervention.

The total duration of the study will be 2 months and 30 days maximum (+ wash-out) per center + patient follow-up over a period of 15 days ± 10.

Once regulatory authorisations have been obtained, the sites will be set up (January 2021), then the actual start-up of the 18 sites will be spread out over a period of about 30 days for feasibility reasons.

Due to the experimental design and the type of intervention, the trial will be conducted as open-label to participants and caregivers. The risk of bias related to the evaluation of the primary outcome in an open-label trial is here limited due to its objective nature (i.e., new infection diagnosed SARS-CoV-2+).

The DEPIST-COVID study will be conducted prior to the ANRS DEPIST trial, the start of which was initially planned for October 2020.
The ANRS DEPIST trial is a randomised, stepped-wedge trial and concerns the evaluation of a nurse-led HIV testing strategy in 18 emergency departments in the Paris Metropolitan area which will, for the most part, be the sites participating in the DEPIST-COVID study, which facilitates its implementation. The DEPIST trial obtained regulatory authorisations. The Coordinating Investigator (J. Leblanc) conducted a study start-up meeting at the 18 sites with the head of the department, the medical contacts and the nursing contacts in July 2020. Regulatory implementation meetings were underway in September 2020.

This document is the property of the DRCI/APHP. Any reproduction is strictly prohibited.
Version No. 4.0 dated 31/05/2019

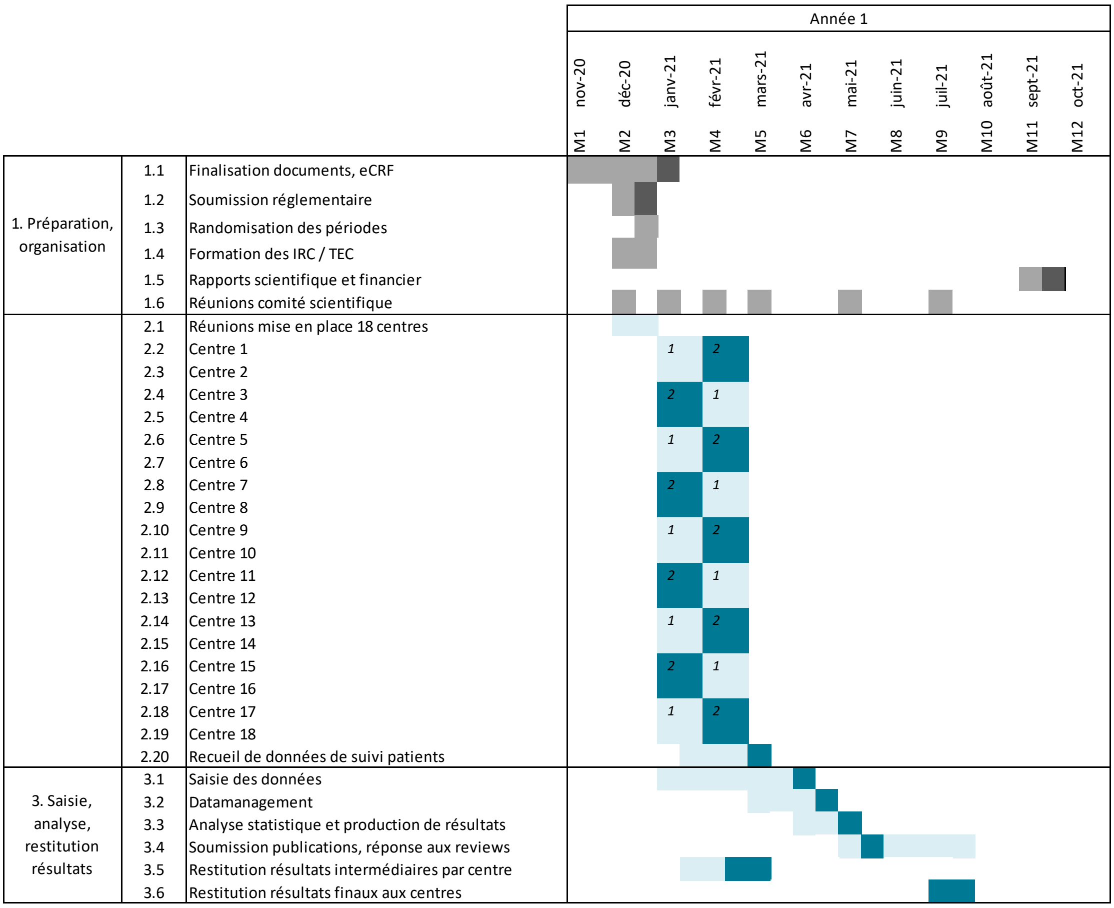

La période 1 correspond à la période Pratique actuelle ;
la période 2 à la période Dépistage SARS-CoV2 systématique + Pratique actuelle.
Les couleurs denses correspondent aux échéances des livrables

5.2 Number of participating sites

The multicentre research will involve 18 emergency departments in the Paris Metropolitan area (see addendum 1).

In each hospital participating in the research, a virologist contact will be identified to facilitate the collection of virology data.

5.3 Description of the measures taken to reduce and avoid bias

1.1.1. Identification of subjects

In this research, the subjects will be identified as follows:
site no. (3 numerical positions) – selection order no. of the person at the site (4 numerical positions)
This reference is unique and will be kept for the duration of the research.

1.1.2. Randomisation

The start-up of the intervention strategy will be randomly assigned for each site following a “cluster and cross-over per period” randomisation.

The randomisation unit will be the site with a stratification on the flow and equipment of the sites in the PCR SARS-CoV-2 automated system. The randomisation list will be drawn up before the start of the research by a biostatistician from the CRU-East independent of the study.

This document is the property of the DRCI/APHP. Any reproduction is strictly prohibited.
Version No. 4.0 dated 31/05/2019

6 CONDUCTING THE RESEARCH

When will people be informed and included in the study?	Individuals who receive an information sheet and whose express consent is requested
All patients will be informed of the study upon admission to the emergency department by signage (see addendum 3). Patients meeting the inclusion criteria (> 18 years) will receive an individual information sheet and will be included unless they do not consent to participate in the study.<br><br>The self-questionnaire will be offered to all patients able to complete it (patients with a life-threatening emergency, with an altered state of consciousness, a severe psychiatric disorder, a language barrier (oral) or deprived of liberty will not be included).	An information sheet (see addendum 4) will be given to patients meeting the inclusion criteria (> 18 years) as soon as he/she is admitted in the emergency department and express consent will be obtained.<br><br>The testing for SARS-CoV-2 by rapid test (multiple molecular/RT-LAMP) will be offered by the emergency department's principal investigator or nursing assistant to patients (or an investigating physician if the testing could not be offered before) regardless of the response to the questionnaire.

6.1 Research schedule

- Enrolment start date: January 2021
- Duration of the inclusion period: 2 months (with possibility of extension to 15 days per period + wash-out)
- Duration of participation for a patient: 1 day (visit to the ED) + telephone call within 15 days ± 10 to retrieve patient profile data if necessary
- Number of sites: 18 emergency departments of the Paris Metropolitan area of which 17 are participants in the DEPIST (HIV) study and including 13 emergency departments of the AP-HP

This document is the property of the DRCI/APHP. Any reproduction is strictly prohibited.
Version No. 4.0 dated 31/05/2019

6.2 Study outline

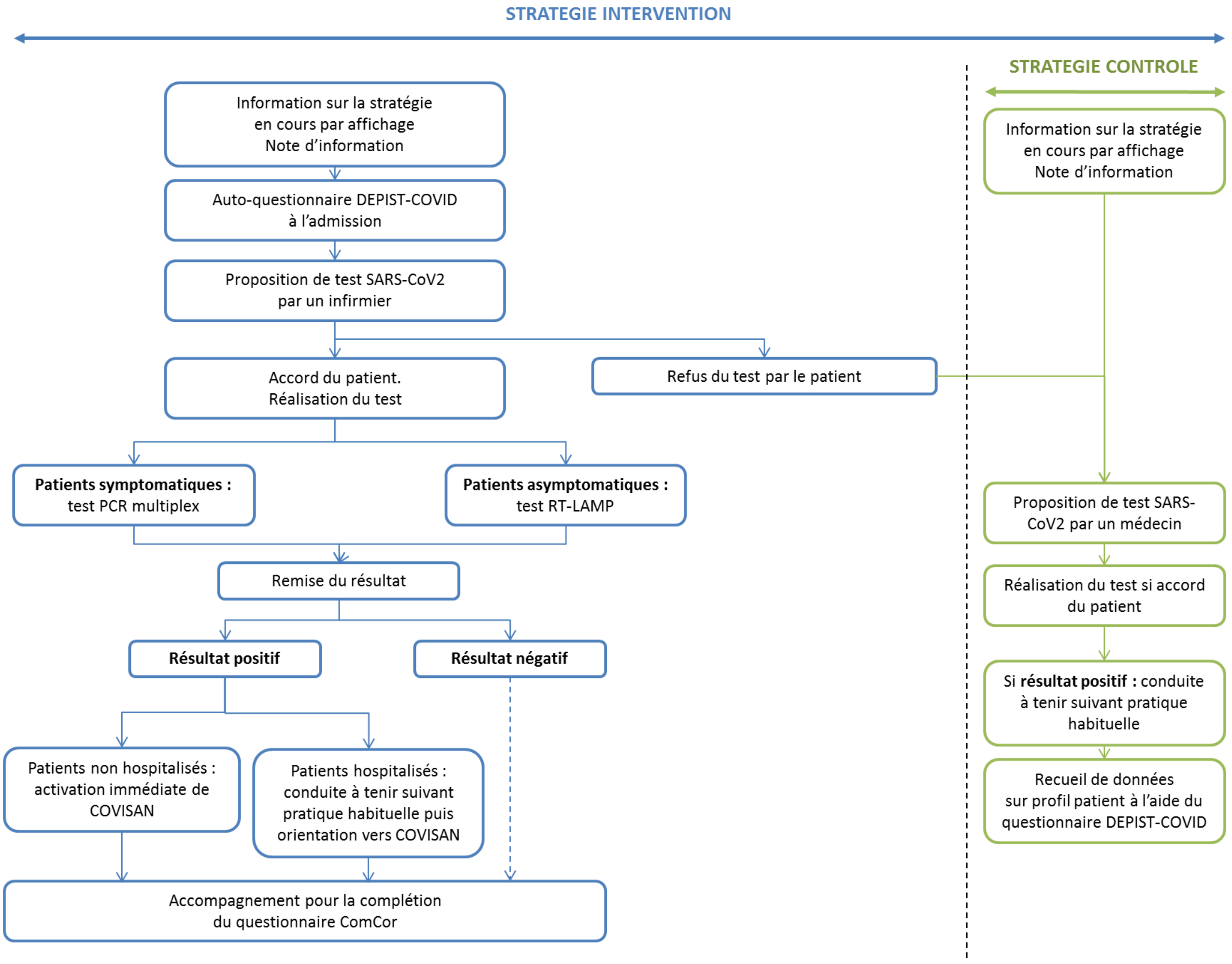

6.3 Practical implementation

6.3.1 Information and training phase

After site randomisation, the research team will contact the 18 participating sites. The testing material is delivered to each site: RT-LAMP tests and multiplex PCR tests. After the implementation meeting, a presentation of the study and material is organised at the start of the intervention period at each site. The research coordinator will conduct staff training in collaboration with the manufacturers providing the multiplex PCR and RT-LAMP tests. The presence of research staff (clinical research nurses and clinical study technicians) 5/7 days trained in the performance of the 2 selected tests will ensure training and support of the teams throughout the intervention period.

6.3.2 Patient information and consent collected

Patients are informed of the DEPIST-COVID study by signage. An information sheet specific to this study is distributed to participants as soon as they are admitted to the emergency department and their express consent is obtained.

This document is the property of the DRCI/APHP. Any reproduction is strictly prohibited.
Version No. 4.0 dated 31/05/2019

6.3.3 Intervention strategy

- A self-administered questionnaire (see addendum 2) on SARS-CoV-2 symptoms, contagion possibilities, situations at risk of exposure as well as socio-demographic characteristics will be offered to enrolled patients who are able to complete it. It was developed by the research team from other questionnaires currently used in France (SocialCov and ComCor questionnaires, Pasteur Institute) or elsewhere [36].
- The patient will fill out the questionnaire and submit it directly to the triage nurse (IOA), or the attending nurse at the time of care. If necessary, the nurse will complete the questionnaire with the patient.
- In the triage room or in a cubicle, SARS-CoV-2 screening will be offered by the nurse regardless of the answer to the questionnaire. According to the decree of 24/07/2020, nurses are authorised to perform, without medical prescription, nasopharyngeal sampling procedures for SARS-CoV-2 infection testing; trained caregivers are also authorised to do so under the responsibility of a physician or a qualified nurse from their institution.
- A nasopharyngeal sample for SARS-CoV-2 test will be taken in a cubicle by the nurse in compliance with the performance conditions and in particular the personal protection measures recommended in the hospital protocol, with the exception of patients who cannot have a nasopharyngeal sample (thrombocytopenia, ENT malformation). In case of contraindication to nasopharyngeal sampling, oropharyngeal sampling is indicated, according to the HAS recommendations [37]. The choice of test will be determined based on the presence of suggestive symptoms.
- The patient will note agreement to participate in the study on page 1.
- A patient with one or more symptoms listed in the patient questionnaire will be considered symptomatic.
- A multiplex PCR test will be performed for patients with symptoms suggestive of SARS-CoV-2 infection, known as “symptomatic”, on medical advice. The analysis time is 75 minutes.
- For patients with no symptoms suggestive of SARS-CoV-2 infection, known as “asymptomatic”, an RT-LAMP test will be performed. The analysis time is 5 to 15 minutes.

In the event of a positive test,

- The patient will be treated according to the circuit defined in each hospital with particular attention to the following points: awareness, isolation measures, and contact tracing/tracking.
- The research staff will ensure that the DEPIST-COVID questionnaire received at the emergency room is completed in an exhaustive manner. Patients who have not completed the DEPIST-COVID questionnaire will be contacted by telephone to complete it.
- Patients who are not hospitalised will be managed via COVISAN or the usual department for follow-up and contact tracing. The scheme will be activated as soon as possible to facilitate the transmission of isolation and transmission risk reduction measures as well as to carry out contact tracing with the testing of household members during a home visit and the search for the source by questionnaire.
- Patients will be accompanied by the research staff to complete the Pasteur Institute paper or online ComCor questionnaire (Addendum 6), depending on availability, to facilitate the study of sociodemographic factors, behaviour, and practices associated with SARS-CoV-2 infection.
- For each positive patient, the next patient with a negative test (and matched on age, sex and area of residence in the last 10 days) will be helped to complete the ComCor questionnaire.

In the event of a negative test, the action to be taken will follow the usual emergency procedure.

This document is the property of the DRCI/APHP. Any reproduction is strictly prohibited.
Version No. 4.0 dated 31/05/2019

In both cases (positive or negative):

- The agreement to participate in the study will be noted on page 4 of the questionnaire and/or by the nurse in the patient file.
- The time of the test and the result will be tracked on the back of the questionnaire and/or in the medical record.
- The team will send the test result to the patient.

6.3.4 Control strategy

The doctor offers a SARS-CoV-2 test according to current recommendations. At the time of writing of this project, the tests are PCR tests prescribed for diagnosis for patients presenting symptoms suggestive of Covid-19 or when screening patients who need to be hospitalised for another reason before being transferred to the hospital department (to avoid hospital clusters). The patient will be cared for according to the circuit defined in each hospital. Patients with a positive test will be asked to complete the DEPIST-COVID questionnaire. Patients who have not completed it will be contacted by telephone to answer questions by phone.

6.3.5 Criteria for choosing tests

The choice of test used is based on available data on sensitivity and specificity, on criteria for handling in the emergency department (storage, number of operations, time for reading, published experiments) and on the test supply capacities. These criteria led to the selection of the following tests with a CE marking:

1 - For patients with symptoms suggestive of respiratory infection: the test is based on a multiplex PCR technique detecting SARS-CoV-2 and all other community-acquired viral infections (Qiastat, Qiagen)[36,37]. This test involves performing a nasopharyngeal swab using a thin, flexible swab with a flocked nylon tip, inserting the swab into the test cassette and inserting it into the Qiastat automated PCR system, which performs all analytical steps. The result is available in 75 minutes. This test does not require any prior technical experience and can therefore be easily performed in an ED. In addition, the direct introduction of the swab into a cassette minimises the handling of an infectious sample and thus limits any risk of secondary contamination.

2 - For asymptomatic patients: an RT-LAMP (Abbott ID NOW, Abbott) test to detect the SARS-CoV-2 genome will be used.

It has several advantages:

- The technique is quick and easy to implement by staff not qualified in biology;
- It can be performed directly after sampling without handling infectious fluids;
- The result is available in less than 15 minutes, thus facilitating patient management;
- Single temperature genome amplification provides a simple and space-saving machine;
- The analytical performances (sensitivity and specificity) are close to those obtained with RT-PCR performed in a laboratory.

The RT-LAMP test is more sensitive than an antigen test and therefore more compatible with a diagnosis performed in a healthcare facility, while the antigen test is more suitable for mass testing in the outpatient department. Positive tests with the RT-LAMP technique performed in asymptomatic patients will not be confirmed by PCR.

This test is also performed using a nasopharyngeal swab. The swab is inserted into a cassette containing reagents that inactivate the virus by heating to 56°C. After inactivation, the viral genome is amplified by using isothermal nucleic acid amplification technology (NAAT) to detect the RNA-dependent RNA polymerase gene (RdRp) segment of the SARS-CoV-2. A real-time analysis

This document is the property of the DRCI/APHP. Any reproduction is strictly prohibited.
Version No. 4.0 dated 31/05/2019

delivers a result after the start of amplification between 5 minutes for the strong positives at 13 minutes for the weak positives and negatives. The machine prints a result label with the patient identification.

This document is the property of the DRCI/APHP. Any reproduction is strictly prohibited.

6.3.6 Summary table of the research chronology

	Intervention strategy	Control strategy
Information for patients by posters in all 18 emergency services (see addendum 3).<br>Distribution of an information sheet (see addendum 2) to patients meeting the inclusion criteria (> 18 years old) and collection of express consent.	R	R
Offer of a self-questionnaire (see addendum 2) to the patients included and able to complete the questionnaire. Agreement to participate in the study noted by the patient on page 1 of the questionnaire	R	
Offer of SARS-CoV-2 testing by the nurse or caregiver, regardless of the answer to the questionnaire	R	
Nasopharyngeal sampling performed and result provided	R (In addition to usual practice, a nasopharyngeal sample RT-LAMP test is performed for asymptomatic patients.)	S<br>(usual practice based primarily on a SARS-CoV-2 diagnosis by PCR in hospitalised patients or symptomatic patients)
For patients tested SARS-CoV-2+ who have not completed questionnaires: collection of data from the DEPIST-COVID questionnaire during a telephone call.	R	R
Assistance in completing a ComCor questionnaire from the Pasteur Institute in paper format (see addendum 6) (or online on the study site as possible), in order to facilitate the study of sociodemographic, behavioural factors and practices associated with SARS-CoV-2 infection.	R	

S: usual practice in the context of Care; R: practice added by the Research

This document is the property of the DRCI/APHP. Any reproduction is strictly prohibited.
Version No. 4.0 dated 31/05/2019

7 SCREENING CRITERIA

7.1 Inclusion criteria

The inclusion criteria will be all adult patients (with no upper age limit) consulting a participating department.

7.2 Non-inclusion criteria

Not applicable

7.3 Recruitment methods

	Number of subjects
Total number of subjects screened	*104,000*
Number of sites	*18*
Inclusion period (months)	*2*
Number of subjects/site	*5,778*
Number of subjects/site/month	***2,889***

8 DISCONTINUATION RULES

8.1 Criteria and procedures for early termination of a subject’s participation in the research

- Any patient may stop participating in the research at any time and for any reason. The reason for this will be sought by the site contact. If the patient is treated during the intervention strategy period, the usual treatment will be adopted.
- For patients lost to follow-up and diagnosed as positive for SARS-CoV-2, the investigator must make every effort to contact the patient again (and document it in the source file).
- The investigator may temporarily or permanently discontinue a subject’s participation in the research for any reason that has an impact on his/her safety or that would best serve the subject’s interests.

In the event that a subject’s research is prematurely terminated, or if consent is withdrawn, his/her data collected before the early termination may be used.

The case report form must list the various reasons for discontinuing participation in the research:

- ☐ Adverse reaction
- ☐ Other medical problem
- ☐ Subject’s personal reason
- ☐ Explicit withdrawal of consent.

This document is the property of the DRCI/APHP. Any reproduction is strictly prohibited.
Version No. 4.0 dated 31/05/2019

8.2 Methods of replacing these persons, if applicable

Subjects who have prematurely stopped their participation in the research following withdrawal of consent will not be replaced.

- Discontinuation of a site's participation in the research

In the event that one (or more) site(s) withdraw before the start of the research, the trial's scientific committee will reassess the selection of a new site that will then be invited to participate.

- Discontinuation of part or all of the research

The sponsor, the AP-HP, reserves the right to permanently suspend enrollment, at any time, if it turns out that the enrollment objectives are not met.

9 EFFICACY ASSESSMENT

2.1. Description of efficacy assessment parameters

The primary outcome, i.e. proportion of patients tested positive for SARS-CoV-2 among the flow of consulting adults, will be evaluated at the end of the 15-day ± 10 follow-up period, based on the patient's medical record. The efficacy data will be analysed according to the statistical analysis plan defined in the relevant section.
The secondary outcomes will be assessed using patient self-administered questionnaire data, virological confirmation data collected from referring physicians, aggregated data from emergency department flows and data collected using locally used software (URQUAL, ORBIS, SUMD, for example).

10 VIGILANCE

In this study, adverse events (serious or not) are not to be reported to the sponsor. The notification must be made within the framework of the vigilance implemented within the context of care for the product or practice that is the subject of the research (pharmacovigilance for a drug, materiovigilance for a medical device, etc.).

11 SPECIFIC RESEARCH COMMITTEES: SCIENTIFIC COMMITTEE

The scientific committee consisting of the coordinating investigator, the scientific manager, the head of the structure responsible for monitoring the research and the teams associated with the project (cited on page 2) will participate in defining the general organisation of the research, will coordinate the information, will initially determine the methodology and will monitor the progress of the research. The committee will meet at the start of the research and then once a month during the inclusion period.

12 DATA MANAGEMENT

12.1 Data collection methods

The data of all patients who have completed a self-administered questionnaire will be collected in an electronic case report form (CleanWeb™). The case report forms will be prepared by the research coordinator in collaboration with the CRU-East/CRC-East.
The CTT/CRN will check that the paper self-questionnaires are completed and that they make it possible to obtain:

This document is the property of the DRCI/APHP. Any reproduction is strictly prohibited.
Version No. 4.0 dated 31/05/2019

- the data of patients over 18 years of age who have completed the self-administered questionnaire as well as the data on the offer, acceptance and completion of SARS-CoV-2 screening tests during the intervention strategy period,
- data from patients over 18 years of age who are tested for SARS-CoV-2 during the control period.

During the 2 periods, the CTT or the CRN will record the general data per site on a daily basis with:

- the total number of patients,
- the number of patients > 18 years,
- the number of patients > 18 years able to give consent (outside of a life-threatening emergency, altered state of consciousness, severe psychiatric disorders, oral language barrier or deprived of liberty),
- the number of patients > 18 years of age who have undergone diagnostic tests for respiratory infection prescribed by emergency physicians as well as virological confirmation data.

These data will be entered directly into an Excel spreadsheet per site on the secure shared space of the Clinical Research Platform of East Paris.

During the 2 periods of the research, the results of the virological analyses of respiratory infection prescribed by emergency physicians to included patients will be collected. Virological data from patients who have performed a SARS-CoV-2 screening test without completing a self-administered questionnaire will also be collected in the electronic case report form (CleanWeb).
Virology results will be reported by the CTT to the referring virologist.
The data will be completed on the paper questionnaire and then entered into Cleanweb by the CTT.

In addition and if possible within the timeframe imposed by the project, the flow data by severity and time of patient visit (including: date and time of entry, month and year of birth, sex, reason for visit given to the triage nurse or IOA, diagnosis at discharge and severity (in order to count patients who are eligible for the self-questionnaire), date and time of discharge) will be collected via an extraction from the clinical activity database used by the sites (URQUAL, ORBIS, SUMD, for example) and which will be anonymised. If necessary, data extraction will be the subject of a request to the dedicated committees (request for authorisation from the scientific and ethics committee of the health data warehouse for the AP-HP services, for example).

12.2 Identification of data collected directly in CRFs that will be considered source data

Data from patient self-administered questionnaires including data on the offer and acceptance of SARS-CoV-2 testing and data on SARS-CoV-2 tests performed and results.

12.3 Right of access to data and source documents

12.3.1 Access to data

In accordance with GCP:
- the sponsor is responsible for obtaining the agreement of all parties involved in the research to ensure direct access to all research sites, source data, source documents and reports for quality control and audit purposes by the sponsor,
- the investigators shall provide individuals responsible for monitoring, quality control and auditing research involving human subjects with the documents and individual data strictly necessary for this control, in accordance with the laws and regulations in force.

This document is the property of the DRCI/APHP. Any reproduction is strictly prohibited.
Version No. 4.0 dated 31/05/2019

12.3.2 Source documents

The source document, defined as any original document or object that can prove the existence or accuracy of a piece of data or fact recorded during the research, will be kept for 15 years by the investigator or by the hospital if it is a hospital medical record.

12.3.3 Confidentiality of data

The persons responsible for quality control of research involving human subjects (Article L.1121-3 of the French Public Health Code), will take all necessary precautions to ensure the confidentiality of information relating to the research project, to the persons participating in it, and in particular with regard to their identity and the results obtained.
These persons, in the same way as the investigators themselves, are subject to professional secrecy (in accordance with the conditions defined by Articles 226-13 and 226-14 of the Criminal Code).
During the research involving human subjects and at its end, the data collected on the participants and sent to the sponsor by the investigators (or any other specialised stakeholders) will be made non-identifying.
Under no circumstances should they clearly show the names of the persons concerned or their address.
Only a coded number specific to the research indicating the order of enrolment of subjects will be recorded.
The sponsor will ensure that each person participating in the research has consented to the collection of individual data concerning him/her that are strictly necessary for the quality control of the research.
The sponsor will also ensure that a sample of patients who have undergone a nurse-led SARS-CoV-2 test has consented to this.

12.4 Data processing and storage of documents and data

12.4.1 Identification of the data controller and the place of data management

Data management will be performed by a data manager of the CRU-East and statistical analysis will be performed by a biostatistician of the CRU-East, under the responsibility of Prof. Tabassome SIMON. The estimation of the incidence of new SARS-CoV-2 infections by modelling will be provided by V. Colizza of the Pierre Louis Institute of Epidemiology and Public Health, INSERM. All data will be sent there.

12.4.2 Data entry

The input of non-identifying data will be done on an electronic medium via an internet browser.

12.5 Data

The AP-HP is the owner of the data and any use or transmission to a third party cannot be made without its prior agreement.

This document is the property of the DRCI/APHP. Any reproduction is strictly prohibited.
Version No. 4.0 dated 31/05/2019

13 STATISTICAL ASPECTS

13.1 Statistical justification for sample size

The estimate of the sample size is based on the comparison of 2 proportions (control group vs intervention group) using a Fisher's exact test, with, for the control group, a proportion of patients tested positive for SARS-CoV-2 among the enrolled patients of 0.1% and a proportion of 0.2% for the intervention group, bilateral α=5% and β=10%.

The proportion of the control group was set using the incidence rate of SARS-CoV-2 among the general population in the Paris Metropolitan area. The incidence rate was 0.12% on 26/11/2020. It was estimated that the proportion of 0.10% was close to the baseline proportion of new SARS-CoV-2 positive cases among a flow of patients in the emergency department with usual screening practices. We estimated that the intervention would lead to a doubling of the proportion, i.e. 0.20%, which represents a substantial clinical gain. The correlations used are extracted from the DICI-VIH study (intra-cluster correlation: rho=0.00014, correlation between-cluster: rho12=0.00009). The calculation was performed with SAS software (version 9.4; SAS Institute, Inc., Cary, NC) and resulted in the need to enrol 104,000 patients [38], i.e. a total enrolment period of 2 months in 18 emergency departments (including 1 month intervention, 1 month control and wash-out).

Simulations were performed using different incidence rates and worst case scenario involving the largest number of patients to be enrolled, i.e. 104,000 patients. The 18 departments confirmed that they had ample potential for inclusion.

The calculation was performed in consultation with V. Colizza, INSERM, involved in the project and M. Cachanado from the biostatistics team of the CRU-East.

According to the feasibility data provided by the emergency departments and based on the experience of two research projects on testing in the emergency department, we estimate that approximately 16,700 tests (RT-LAMP and PCR multiplex) could be performed by nurses during the intervention period among 50,000 eligible patients, i.e. approximately 930 tests per site in 1 month and approximately 31 tests per day per site (19 RT-LAMP and 12 PCR multiplex per day).

13.2 Description of the statistical methods

The analysis will be performed at the end of the research, after a data review meeting and freezing of the database. An analysis plan will be drafted and any changes will be documented.

The characteristics of the sites and patients will be described. The proportion of patients tested positive for other respiratory viruses will be described. Qualitative data will be described by means of numbers and proportions, and quantitative variables will be described using means, standard deviations, ranges, medians and interquartile ranges.

The primary analysis will be performed according to the intent-to-treat (ITT) principle. The primary outcome will be analysed using a generalised linear mixed model (Poisson distribution or any other distribution adapted to the data) with the strategy as a fixed effect, the centre x strategy interaction as a random effect and the centre as a random intercept.

For the other secondary outcomes:

This document is the property of the DRCI/APHP. Any reproduction is strictly prohibited.
Version No. 4.0 dated 31/05/2019

- The proportion of patients tested positive for SARS-CoV-2 among the asymptomatic patients tested and that in the Paris Metropolitan area at the same period will be compared using an asymptotic Wald test.
  The proportion obtained in each emergency department will be compared to that of the department's IRIS geographical area using an asymptotic Wald test.
  The same comparisons will be made among the symptomatic patients tested and across the entire tested population.

- The feasibility of the testing will be described: proportion of patients who have completed the questionnaire, proportion of offered, accepted and performed tests, and notified of the SARS-CoV-2 result and staff involved (nurses, caregivers, doctors, others),

- The proportion of patients tested positive for SARS-CoV-2 among the patients tested, the proportion of asymptomatic patients among the patients tested and the proportion of asymptomatic patients among the patients tested positive for SARS-CoV-2 will be described in the 2 strategies.

- The symptoms of patients tested positive for SARS-CoV-2 will be described in both strategies and in the total population.

- Exposure factors will be described in both strategies using the items in the DEPIST-COVID questionnaire. The exposure factors associated with the positivity of the SARS-CoV-2 test will be analysed using a generalised linear mixed model (binomial distribution or other distribution adapted to the data). The exposure factors will be considered fixed effects and the site as a random intercept.

- The sociodemographic, behavioural and practical factors of diagnosed patients associated with positive SARS-CoV-2 infection will be described for the three schemes (DEPIST-COVID, ComCor and COVISAN) according to the types of variables (proportion, mean and standard deviation or median, Q1-Q3) and presented with their 95% confidence interval. The factors will also be analysed in the population of DEPIST-COVID cases (positive tested patients) and controls (negative and matched tested patients).

- The number of contact persons tested which are SARS-CoV-2 positive and have isolated in both periods will be described.

Missing values from the questionnaires will not be replaced; this strategy will be reassessed according to the proportions of missing values obtained.

Estimation of the incidence of COVID-19 cases in the population in the Paris Metropolitan area.

We will consider the cohort of patients arriving at the emergency department over time and the proportion of positive tests for COVID-19. The incidence rate among patients during a given week will be calculated as the ratio between the number of positive tested patients during that week and the total number of patients admitted to the emergency room during that week. The ratio will first be calculated by age group (for example: 0-18; 19-40; 40-64; 65+) and by sex, and weighted by the proportion of the general population of the strata in the region. The confidence interval of the estimate is obtained using a rescaled Poisson distribution (DKES estimator) [39]. The results will be compared to those from an age-stratified mathematical that has already been validated and used to estimate the impact of lockdown during the first wave and for the proposal of exit strategies [40]. Calibrated on the number of hospitalisations in the region, this model allows us to estimate the total number of cases and therefore the detection rate of virological monitoring in region [41].

This document is the property of the DRCI/APHP. Any reproduction is strictly prohibited.
Version No. 4.0 dated 31/05/2019

14 ANCILLARY STUDY

See attached protocol "Depist-Covid ancillary study"

15 QUALITY CONTROL AND ASSURANCE

15.1 General organisation

The sponsor must ensure the safety and respect of the people who have agreed to participate in the research. It must set up a quality assurance system to best monitor the conduct of the research at the research sites.

To this end, the sponsor will define a strategy for opening the sites and may, if necessary, implement data quality control.

15.1.1 Strategy for opening sites

The site opening strategy will be determined before the start of the research.

15.1.2 Data quality control

A Clinical Research Associate (CRA) appointed by the sponsor will ensure the proper conduct of the research, the collection of data generated in writing, their documentation, recording and report, in accordance with the Standard Operating Procedures implemented within the DRCI.
The investigator and the members of his/her team agree to make themselves available during quality control visits carried out at regular intervals by the Clinical Research Associate.

15.1.3 Case report form

All information required by the protocol must be recorded in the case report forms. The data must be collected as they are obtained, and be precisely recorded in these forms. Each missing piece of data must be coded.
This electronic case report form will be implemented at each site using an Internet data collection medium. A document to assist in the use of this tool will be provided to the investigators.
The completion of the case report form via the internet by the investigator will allow the CRA to quickly view the data remotely. The investigator is responsible for the accuracy, quality, and relevance of all data entered. In addition, when they are entered, these data are immediately verified through consistency checks. As such, the investigator must validate any change in value in the CRF. These changes are subject to an audit trail. A justification may be included in the comments.
A paper printout will be requested at the end of the study, authenticated (dated and signed) by the investigator. The original of this document will be archived by the sponsor. A copy of the authenticated document must be archived by the investigator.

15.2 Management of non-compliance

Any event occurring as a result of non-compliance with the protocol, standard operating procedures, or legislative and regulatory provisions in force by an investigator or any other person involved in the conducting of the research must be the subject of a non-compliance report to the sponsor.

This document is the property of the DRCI/APHP. Any reproduction is strictly prohibited.
Version No. 4.0 dated 31/05/2019

These non-compliances will be managed in accordance with the sponsor's procedures.

15.3 Audit

The investigators undertake to accept the quality assurance audits carried out by the sponsor as well as the inspections carried out by the competent authorities. All data, documents and reports may be subject to audits and regulatory inspections without the possibility of medical confidentiality.

An audit may be conducted at any time by persons appointed by the sponsor and independent of the persons in charge of the research. Its purpose is to ensure the quality of the research, the validity of its results, and compliance with the law and regulations in force.

The persons who direct and monitor the research agree to comply with the sponsor's requirements regarding an audit

The audit may apply to all stages of the research, from the development of the protocol, to the publication of the results, and the classification of the data used or produced as part of the research.

15.4 Signed Principal Investigator's incurred liabilities

Before starting the research, each investigator will provide the research sponsor's representative with a copy of his/her dated and signed staff curriculum vitæ and carrying his/her RPPS [*Répertoire Partagé des Professionnels de Santé* (Collective Database of Health Professionals)] number, or ADELI [*Automatisation des listes* (List Automation)] number. The CV must include previous participation in research and training related to clinical research.

Each investigator commits to comply with legislative and regulatory obligations and to conduct the research in accordance with the regulations, while abiding by the terms of the Declaration of Helsinki in force.

The principal investigator of each participating site will sign a commitment of responsibilities (DRCI-type document) which will be given to the sponsor's representative.

The investigators and their collaborators will sign a delegation of functions form specifying the role of each and will provide their CVs.

16 ETHICAL AND LEGAL ASPECTS

16.1 Information and consent collection procedures for research participants

An existing information sheet in several languages will be distributed during the two periods to all eligible emergency consulting patients and express consent will be collected (see addendum 4). The patient will also note his/her agreement to participate on page 1 of the questionnaire and/or the nurse will mention it in the medical record.

16.2 Prohibition for the person to participate in another research study or exclusion period provided for at the end of the research, if applicable

During his/her participation in the research, the subject may not participate in another research protocol involving human subjects without discussing it with the doctor who is monitoring him/her as part of the research.

This document is the property of the DRCI/APHP. Any reproduction is strictly prohibited.
Version No. 4.0 dated 31/05/2019

However, subjects may participate in other non-interventional type research. Simultaneous participation in another interventional research study not involving SARS-CoV-2 screening is authorised.

16.3 Compensation of subjects

16.3.1 Reimbursement of expenses incurred

Not applicable

16.3.2 Indemnification

Not applicable

16.4 Registration in the national register of individuals participating in research involving human subjects

Not applicable

16.5 Legal obligations

Public Hospitals of Paris (AP-HP) is the sponsor for this research and, by delegation, the Department of Clinical Research and Development (DRCD) undertakes its tasks, in accordance with Article L.1121-1 of the French Public Health Code. Public Hospitals of Paris reserves the right to discontinue the research at any time for medical or administrative reasons; in this case, the investigator will be notified.

16.6 Request for opinion from the Ethics Committee EC

The AP-HP, as the sponsor, obtains the favourable opinion of the relevant EC for the interventional research with minimal risk and constraints, prior to its implementation, within the scope of its competences and in accordance with the laws and regulations in force.

16.7 Information from the ANSM [*Agence nationale de sécurité du médicament et des produits de santé* (French National Agency for Medicines and Health Products Safety)]

The sponsor, the AP-HP, will send the favourable opinion of the EC and the protocol summary to the ANSM for information.

16.8 Procedures relating to IT regulations and its freedoms

The computer file used for this research is implemented in accordance with French (modified Data Protection Act) and European (General Data Protection Regulation - GDPR) regulations.

- Commitment of compliance with the “Reference methodology” MR 001

This research falls within the framework of the “Reference Methodology for the processing of personal data implemented in the context of research in the health field” (MR-001 as amended). The AP-HP, the research sponsor, has signed a commitment to comply with this “Reference Methodology”.

16.9 Amendments to the research

Any substantial amendment made to the protocol by the coordinating investigator must be sent to the sponsor for approval. After this agreement, the sponsor must obtain a favourable opinion from the EC prior to its implementation.
The information sheet and consent form may be revised if necessary, particularly in the event of a substantial amendment to the research or the occurrence of adverse effects.

This document is the property of the DRCI/APHP. Any reproduction is strictly prohibited.
Version No. 4.0 dated 31/05/2019

16.10 Final research report

The final report of the research involving human subjects mentioned in Article R1123-67 of the PHC is prepared and signed by the sponsor and the investigator. A summary of the report written according to the reference plan of the competent authority must be sent to the competent authority within one year, after the end of the research, corresponding to the end of the participation of the last person participating in the research.

16.11 Archiving

The specific documents of a research project involving human subjects at risk and minimal constraints will be archived by the investigator and the sponsor for a period of 15 years after the end of the research project.
In particular, this indexed archiving involves:

- The "research" folders for the Investigator and the sponsor including (non-exhaustive list):
  - The successive versions of the protocol (identified by the version number and version date)
  - the EC's opinions
  - letters of correspondence
  - the inclusion list or register
  - the appendices specific to the research
  - Final research report
- The data collection documents

17 FINANCING AND INSURANCE

17.1 Source of funding

ANRS – REACTing and the AP-HP

17.2 Insurance

The Sponsor shall take out, for the entire duration of the research, insurance covering its own civil liability as well as that of any doctor involved in the conduct of the research. It also provides full compensation for the harmful consequences of the research to the person taking part and his/her beneficiaries, unless it can prove that the damage is not attributable to its fault or to any other party involved, without the possibility of invoking the act of a third party or the voluntary withdrawal of the person who had initially agreed to participate in the research.

Public Hospitals of Paris (AP-HP) has taken out insurance with the company HDI-GLOBAL SE through BIOMEDIC-INSURE, guaranteeing its civil liability as well as that of any participant (doctor or staff involved in conducting the research), in accordance with Article L.1121-10 of the PHC.

18 RULES REGARDING PUBLICATION

The rank of authors will be defined based on the effective contribution of each site referent to recruitment and each member of the scientific committee to the design, implementation of the study and writing of the article, according to rules that will be defined during the first meetings of study participants.

This document is the property of the DRCI/APHP. Any reproduction is strictly prohibited.
Version No. 4.0 dated 31/05/2019

The ranking of authors will be as follows: J. Leblanc (first author), T. Simon (penultimate author), A.-C. Crémieux (last author).

18.1 Mention of the AP-HP affiliation for the projects sponsored by the AP-HP

The AP-HP will be mentioned in the affiliations of the authors of the publications that will result from this research and a copy of the publication will be sent to the DRCD.

- If an author has several affiliations, the order in which the institutions are cited (the AP-HP, University, INSERM, etc.) does not matter,
- Each of these affiliations must be identified by an address separated by a semi-colon,
- The AP-HP institution must appear under the acronym "AP-HP" first in the address followed precisely by: The AP-HP, hospital, department, city, postal code, France.

18.2 Mention of the sponsor the AP-HP (DRCI) in the "acknowledgements" of the manuscript

The AP-HP (DRCD) will be mentioned as being the sponsor of the research as follows:
"The sponsor was Public Hospitals of Paris (Delegation for Clinical Research and Innovation)"

18.3 Mention of the funding body in the "acknowledgements" of the manuscript

The project funding sources will be mentioned, as follows:
This research will be registered on the site http://clinicaltrials.gov/ NCT04756609

This document is the property of the DRCI/APHP. Any reproduction is strictly prohibited.
Version No. 4.0 dated 31/05/2019

19 BIBLIOGRAPHY

1. Dashboard by the Center for Systems Science and Engineering, The John Hopkins University. https://coronavirus.jhu.edu/map.html. Published: April 2021. Accessed date: 08/04/2021
2. Ryu S, Ali ST, Jang C, Kim B, Cowling BJ. Effect of Nonpharmaceutical Interventions on Transmission of Severe Acute Respiratory Syndrome Coronavirus 2, South Korea, 2020. *Emerg Infect Dis*. Jun 2 2020;26(10)doi:10.3201/eid2610.201886
3. Pan A, Liu L, Wang C, et al. Association of Public Health Interventions With the Epidemiology of the COVID-19 Outbreak in Wuhan, China. *JAMA*. Apr 10 2020;323(19):1-9. doi:10.1001/jama.2020.6130
4. Reintjes R. Lessons in contact tracing from Germany. *BMJ*. Jun 25 2020;369:m2522. doi:10.1136/bmj.m2522
5. Han E, Tan MMJ, Turk E, et al. Lessons learnt from easing COVID-19 restrictions: an analysis of countries and regions in Asia Pacific and Europe. *Lancet*. Nov 7 2020;396(10261):1525-1534. doi:10.1016/s0140-6736(20)32007-9
6. MacIntyre CR. Case isolation, contact tracing, and physical distancing are pillars of COVID-19 pandemic control, not optional choices. *Lancet Infect Dis*. Jun 15 2020;doi:10.1016/s1473-3099(20)30512-0
7. ECDC. Updated rapid risk assessment from ECDC on coronavirus disease (COVID-19) pandemic in the EU/EEA and the UK: resurgence of cases. *Euro Surveill*. Aug 2020;25(32)doi:10.2807/1560-7917.Es.2020.25.32.2008131
8. Chang S, Pierson E, Koh PW, et al. Mobility network models of COVID-19 explain inequities and inform reopening. *Nature*. Nov 10 2020;doi:10.1038/s41586-020-2923-3
9. Pullano G, Di Domenico L, Sabbatini CE, et al. Underdetection of COVID-19 cases in France in the exit phase following lockdown. *medRxiv (accepted in Nature)*. 2020:2020.08.10.20171744. doi:10.1101/2020.08.10.20171744
10. Santé publique France. Synthèse rapide COVID-19. Part des formes asymptomatiques et transmission du SARS-CoV-2 en phase pré-symptomatique. Saint-Maurice. https://www.santepubliquefrance.fr/maladies-et-traumatismes/maladies-et-infections-respiratoires/infection-a-coronavirus/documents/synthese-rapide-des-connaissances/part-des-formes-asymptomatiques-et-transmission-du-sars-cov-2-en-phase-pre-symptomatique.-synthese-rapide-covid-19. Published : July 2020. Accessed date: 10/09/2020
11. Kimball A, Hatfield KM, Arons M, et al. Asymptomatic and Presymptomatic SARS-CoV-2 Infections in Residents of a Long-Term Care Skilled Nursing Facility - King County, Washington, March 2020. *MMWR Morb Mortal Wkly Rep*. Apr 3 2020;69(13):377-381. doi:10.15585/mmwr.mm6913e1
12. Lee S, Kim T, Lee E, et al. Clinical Course and Molecular Viral Shedding Among Asymptomatic and Symptomatic Patients With SARS-CoV-2 Infection in a Community Treatment Center in the Republic of Korea. *JAMA Intern Med*. Aug 6 2020;doi:10.1001/jamainternmed.2020.3862
13. Zhang J, Wu S, Xu L. Asymptomatic carriers of COVID-19 as a concern for disease prevention and control: more testing, more follow-up. *Biosci Trends*. Jul 17 2020;14(3):206-208. doi:10.5582/bst.2020.03069
14. He X, Lau EHY, Wu P, et al. Temporal dynamics in viral shedding and transmissibility of COVID-19. *Nat Med*. May 2020;26(5):672-675. doi:10.1038/s41591-020-0869-5
15. Kretzschmar ME, Rozhnova G, Bootsma MCJ, van Boven M, van de Wijgert J, Bonten MJM. Impact of delays on effectiveness of contact tracing strategies for COVID-19: a modelling study. *Lancet Public Health*. Aug 2020;5(8):e452-e459. doi:10.1016/s2468-2667(20)30157-2
16. ECDC. European Centre for Disease Prevention and Control. Population-wide testing of SARS-CoV-2: country experiences and potential approaches in the EU/EEA and the UK Stockholm. https://www.ecdc.europa.eu/en/publications-data/population-wide-testing-sars-cov-2-country-experiences-and-potential-approaches#no-link. Published on: August 2020. Accessed date: 07/03/2021
17. Grassly NC, Pons-Salort M, Parker EPK, White PJ, Ferguson NM. Comparison of molecular testing strategies for COVID-19 control: a mathematical modelling study. *Lancet Infect Dis*. Aug 18 2020;doi:10.1016/s1473-3099(20)30630-7

This document is the property of the DRCI/APHP. Any reproduction is strictly prohibited.
Version No. 4.0 dated 31/05/2019

18. Firth JA, Hellewell J, Klepac P, Kissler S, Kucharski AJ, Spurgin LG. Using a real-world network to model localized COVID-19 control strategies. *Nat Med*. Oct 2020;26(10):1616-1622. doi:10.1038/s41591-020-1036-8
19. Peto J, Alwan NA, Godfrey KM, et al. Universal weekly testing as the UK COVID-19 lockdown exit strategy. *Lancet*. May 2 2020;395(10234):1420-1421. doi:10.1016/s0140-6736(20)30936-3
20. Gudbjartsson DF, Helgason A, Jonsson H, et al. Spread of SARS-CoV-2 in the Icelandic Population. *N Engl J Med*. Jun 11 2020;382(24):2302-2315. doi:10.1056/NEJMoa2006100
21. Gudbjartsson DF, Norddahl GL, Melsted P, et al. Humoral Immune Response to SARS-CoV-2 in Iceland. *N Engl J Med*. Sep 1 2020;doi:10.1056/NEJMoa2026116
22. Iacobucci G. Covid-19: Mass population testing is rolled out in Liverpool. *BMJ*. Nov 3 2020;371:m4268. doi:10.1136/bmj.m4268
23. Mahase E. Covid-19: Mass testing in Slovakia may have helped cut infections. *BMJ*. Dec 8 2020;371:m4761. doi:10.1136/bmj.m4761
24. Pullano G, Valdano E, Scarpa N, Rubrichi S, Colizza V. Evaluating the effect of demographic factors, socioeconomic factors, and risk aversion on mobility during the COVID-19 epidemic in France under lockdown: a population-based study. *The Lancet Digital Health*. 2020;2(12):e638-e649. doi:10.1016/S2589-7500(20)30243-0
25. Wise J. Covid-19: Concerns persist about purpose, ethics, and effect of rapid testing in Liverpool. *BMJ*. Dec 2 2020;371:m4690. doi:10.1136/bmj.m4690
26. Guthe T. Prevention of venereal infections. *Bull World Health Organ*. 1958;19(3):405-26.
27. Centers for Disease Control and Prevention. Revised recommendations for HIV testing of adults, adolescents, and pregnant women in health-care settings. http://www.cdc.gov/mmwr/preview/mmwrhtml/rr5514a1.htm. Published: December 2006. Accessed date: 03/10/2016
28. Haute Autorité de Santé. Dépistage de l'infection par le VIH, Stratégies et dispositif de dépistage, Synthèse et recommandations. http://www.sante.gouv.fr/IMG/pdf/synthese_depistage_vih_HAS_2009-2.pdf. Published: October 2009. Accessed date: 13/09/2018
29. d'Almeida KW, Kierzek G, de Truchis P, et al. Modest public health impact of nontargeted human immunodeficiency virus screening in 29 emergency departments. *Arch Intern Med*. 2012;172(1):12-20. doi:10.1001/archinternmed.2011.535
30. DREES. Les établissements de santé. Edition 2017. Panoramas de la DREES Santé, Direction de la recherche, des études, de l'évaluation et des statistiques. http://drees.solidarites-sante.gouv.fr/etudes-et-statistiques/publications/panoramas-de-la-drees/article/les-etablissements-de-sante-edition-2017. Published: June 2017. Accessed date: 10/08/2018
31. CREU. Commission Régionale d'Experts Urgences Île-de-France (CREU). Activité des services d'urgences Ile-de-France. https://www.iledefrance.ars.sante.fr/sites/default/files/2017-02/Urgences-CREU-Rapport-IDF-2015.pdf. Published: November 2016. Accessed date: 13/10/2020
32. Santé publique France. COVID-19 : Point épidémiologique du 03 septembre 2020. https://www.santepubliquefrance.fr/maladies-et-traumatismes/maladies-et-infections-respiratoires/infection-a-coronavirus/documents/bulletin-national/covid-19-point-epidemiologique-du-3-septembre-2020. Published: September 2020. Accessed date: 10/09/2020
33. Leblanc J, Hejblum G, Costagliola D, et al. Targeted HIV Screening in Eight Emergency Departments: The DICI-VIH Cluster-Randomized Two-Period Crossover Trial. *Ann Emerg Med*. Oct 30 2017;doi:10.1016/j.annemergmed.2017.09.011
34. Marty L, Cazein F, Panjo H, Pillonel J, Costagliola D, Supervie V. Revealing geographical and population heterogeneity in HIV incidence, undiagnosed HIV prevalence and time to diagnosis to improve prevention and care: estimates for France. *J Int AIDS Soc*. Mar 2018;21(3):e25100. doi:10.1002/jia2.25100
35. Viner RM, Mytton OT, Bonell C, et al. Susceptibility to and transmission of COVID-19 amongst children and adolescents compared with adults: a systematic review and meta-analysis. *medRxiv*. 2020:2020.05.20.20108126. doi:10.1101/2020.05.20.20108126
36. Fisher KA, Tenforde MW, Feldstein LR, et al. Community and Close Contact Exposures Associated with COVID-19 Among Symptomatic Adults ≥18 Years in 11 Outpatient Health Care Facilities - United States, July 2020. *MMWR Morb Mortal Wkly Rep*. Sep 11 2020;69(36):1258-1264. doi:10.15585/mmwr.mm6936a5
37. Haute Autorité de Santé. COVID-19 : avis favorable au prélèvement oropharyngé en cas de contre-indication au nasopharyngé. https://www.has-sante.fr/jcms/p_3203097/fr/covid-19-avis-

This document is the property of the DRCI/APHP. Any reproduction is strictly prohibited.
Version No. 4.0 dated 31/05/2019

favorable-au-prelevement-oropharynge-en-cas-de-contre-indication-au-nasopharynge. Published: Sept 2020. Accessed date: 09/10/2020
38. Giraudeau B, Ravaud P, Donner A. Sample size calculation for cluster randomized cross-over trials. *Stat Med*. Nov 29 2008;27(27):5578-85. doi:10.1002/sim.3383
39. Dobson AJ, Kuulasmaa K, Eberle E, Scherer J. Confidence intervals for weighted sums of Poisson parameters. *Stat Med*. Mar 1991;10(3):457-62. doi:10.1002/sim.4780100317
40. Di Domenico L, Pullano G, Sabbatini CE, Boëlle PY, Colizza V. Impact of lockdown on COVID-19 epidemic in Île-de-France and possible exit strategies. *BMC Med*. Jul 30 2020;18(1):240. doi:10.1186/s12916-020-01698-4
41. Pullano G, Di Domenico L, Sabbatini CE, et al. Underdetection of COVID-19 cases in France in the exit phase following lockdown. *medRxiv*. 2020:2020.08.10.20171744. doi:10.1101/2020.08.10.20171744

This document is the property of the DRCI/APHP. Any reproduction is strictly prohibited.
Version No. 4.0 dated 31/05/2019

20 LIST OF ADDENDA

Addendum 1: List of participating sites and investigators

Addendum 2: Consultant's self-questionnaire

Addendum 3: Posters

Addendum 4: Information sheet for the consultant

Addendum 5: Information sheet for the consultant for the ancillary study

Addendum 6: DEPIST-COVID reference case questionnaire

This document is the property of the DRCI/APHP. Any reproduction is strictly prohibited.
Version No. 4.0 dated 31/05/2019

Final statistical analysis plan
English version

1 FINAL ANALYSIS PLAN

1.1 Description of planned statistical methods

Descriptive analysis

The characteristics of the sites and patients will be described. The proportion of patients tested positive for other respiratory viruses will be described. Qualitative data will be described by means of numbers and proportions, and quantitative variables will be described using means, standard deviations, ranges, medians and interquartile ranges. A flow chart presenting the population included and analysed will be presented.

Primary endpoint analysis

The primary endpoint is the proportion of patients tested positive for SARS-CoV-2 amongst the flow of consulting adults. The denominator is a number of patients in the emergency departments (and not the number of tests performed).

Patients considered positive for SARS-CoV-2 will be those with a positive test result and whose SARS-CoV2+ status is not known. Patients considered non-positive for SARS-CoV-2 will be patients not tested, patients tested negative or with an undeterminate result and patients tested positive with a known SARS-CoV2+ status.

The primary analysis will be performed according to the intent-to-treat (ITT) principle. The primary outcome will be analysed using a generalised linear mixed model (Poisson distribution or any other distribution adapted to the data) with the centre as a random intercept and the strategy, the period and the period x strategy interaction as fixed effects. The incidence of SARS-CoV2 or the evolution of the epidemic over time will be taken into account if appropriate. Fixed variables could be added to the model if appropriate.

Secondary endpoints analyses

- The proportion of patients tested positive for SARS-CoV-2 among the asymptomatic or paucisymptomatic patients tested by nurses and that in the Paris Metropolitan area at the same period will be compared using an asymptotic Wald test.
  The proportion obtained in each emergency department will be compared to that of the department's IRIS geographical area using an asymptotic Wald test. The same comparisons will be made across the entire tested population.

- The feasibility of the testing will be described: proportion of patients who have completed the questionnaire, proportion of offered, accepted and performed tests, and notified of the SARS-CoV-2 result and staff involved (nurses, caregivers, doctors, others),

- The proportion of patients tested positive for SARS-CoV-2 among the patients tested by nurses, the proportion of asymptomatic patients among the patients tested by nurses and the proportion of asymptomatic patients among the patients tested positive for SARS-CoV-2 will be described.

- The symptoms of patients tested positive for SARS-CoV-2 will be described in the patients completed the questionnaire.

- Exposure factors will be described using the items in the DEPIST-COVID questionnaire. Among patients tested, the exposure factors associated with the positivity of the SARS-CoV-2 test will be analysed using a generalised linear mixed model (binomial distribution or other distribution adapted to the data). The exposure factors will be considered as fixed effects and the site as a random intercept. Other fixed variables could be added to the model if appropriate.

- The sociodemographic, behavioural and practical factors of diagnosed patients associated with positive SARS-CoV-2 infection will be described for the schemes (DEPIST-COVID, ComCor) according to the types of variables (proportion, mean and standard deviation or median, Q1-Q3) and presented with their 95% confidence interval. If appropriate, the factors will also be analysed in the population of DEPIST-COVID cases (positive tested patients) and controls (negative and matched tested patients).

URC-Est, AP-HP will be in charge of the analysis using SAS software, Version 9.4 (SAS Institute, Inc., Cary, NC) and R freeware, Version 4.1.3 (R Core Team, 2022).

Estimation of the incidence of COVID-19 cases in the population in the Paris Metropolitan area.
We will consider the cohort of patients arriving at the emergency department over time and the proportion of positive tests for COVID-19. The incidence rate among patients during a given week will be calculated as the ratio between the number of positive tested patients during that week and the total number of patients admitted to the emergency room during that week. The ratio will first be calculated by age group (for example: 0-18; 19-40; 40-64; 65+) and by sex, and weighted by the proportion of the general population of the strata in the region. The confidence interval of the estimate is obtained using a rescaled Poisson distribution (DKES estimator). The results will be compared to those from an age-stratified mathematical that has already been validated and used to estimate the impact of lockdown during the first wave and for the proposal of exit strategies. Calibrated on the number of hospitalisations in the region, this model allows us to estimate the total number of cases and therefore the detection rate of virological monitoring in region [41].

IPLESP, SUMO, INSERM will be in charge of the analysis.

1.2 Hypotheses for calculating the required number of subjects

The estimate of the sample size is based on the comparison of 2 proportions (control group vs intervention group) using a Fisher's exact test, with, for the control group, a proportion of patients tested positive for SARS-CoV-2 among the enrolled patients of 0.1% and a proportion of 0.2% for the intervention group, bilateral α=5% and β=10%.

The proportion of the control group was set using the incidence rate of SARS-CoV-2 among the general population in the Paris Metropolitan area. The incidence rate was 0.12% on 26/11/2020. It was estimated that the proportion of 0.10% was close to the baseline proportion of new SARS-CoV-2 positive cases among a flow of patients in the emergency department with usual screening practices.

The correlations used are extracted from the DICI-VIH study (intra-cluster correlation: rho=0.00014, correlation between-cluster: rho12=0.00009). The calculation was performed with SAS software (version 9.4; SAS Institute, Inc., Cary, NC) and resulted in the need to enrol 104,000 patients [38], i.e. a total enrolment period of 2 months in 18 emergency departments (including 1 month intervention, 1

month control and wash-out). Simulations were performed using different incidence rates and worst case scenario involving the largest number of patients to be enrolled, i.e. 104,000 patients.

1.3 Method for taking into account missing, unused or invalid data

Missing values for the primary outcome will not be replaced.
Missing values from the questionnaires will not be replaced; this strategy will be reassessed according to the proportions of missing values obtained.

1.4 Selection of populations

The primary analysis will be performed according to the intent-to-treat (ITT) principle. The primary outcome, i.e. proportion of patients tested positive for SARS-CoV-2 among the flow of consulting adults, will be evaluated at the end of the 15-day ± 10 follow-up period, based on the patient's medical record.

For primary and secondary outcomes, data will be extracted from:

- patient self-administered questionnaire data (DEPIST-COVID),
- from virological confirmation data collected from referring physicians,
- aggregated data from emergency department flows,
- and data collected using locally used software (URQUAL, ORBIS, SUMD, for example).

1.5 Summary of the main changes since the validation of the protocol

For the primary endpoint, the analysis plan was revised to mention that the incidence of SARS-CoV2 or the evolution of the epidemic over time could be taken into account and that fixed variables could be added to the model. Theperiod x strategy interaction is usually added as a fixed variable and this was corrected from the initial analysis plan.
The analysis plan was revised for the secondary endpoints due to data that could not be collected. Symptoms, exposure factors and follow up data in the COVISAN monitoring system were not collected for patients tested outside of DEPIST-COVID screening during the intervention period and for patients tested during the control period. Data were also not collected for close contact persons. Objectives and analysis plan were adapted accordingly.

	Coordinator / biostatistician	Coordinator data management and statistic URC-Est
First name Name	Judith LEBLANC	Alexandra ROUSSEAU
Date	21/04/2022	22/04/2022
Signature		

**Study**	DEPIST-COVID
**Promotion/gestion code**	APHP201625
**Study coordinator**	J. LEBLANC

Changes to the analysis plan

Primary endpoint analysis

The primary outcome will be analysed using a generalised linear mixed model (Poisson distribution or any other distribution adapted to the data) with the centre as a random intercept, the centre-by-period interaction as random effect and the strategy, the period and the incidence rate-by-period interaction as fixed effects. The logarithm of the number of patients will be included as an offset term in the model.
The period will be modelled as weekly (1 to 15).
Patients considered non-positive for SARS-CoV-2 will be patients not tested, patients tested negative or with an undeterminate result and patients tested positive with a known SARS-CoV2+ status for less than 3 months.

Secondary endpoints analyses

- Factors associated with performing systematic nurse-driven screening will be analysed in patients who had screening offer using a generalised linear mixed model (binomial distribution) with the centre as a random intercept and the patient characteristics as fixed effects.

- The factors associated with new SARS-CoV-2 diagnoses will be analysed using a generalised linear mixed model (binomial distribution) with the centre as a random intercept and patient characteristics as fixed effects.

- The proportions of new SARS-CoV-2 diagnoses through systematic nurse-driven screening overall and per ED, and proportion among all patients tested in EDs, will be compared to the proportion of positive tests among adults tested in the geographical departments of the Paris Metropolitan area using the Miettinen-Nurminen method for risk difference and 95% CI estimations.

DEPIST-COVID STUDY

Systematic Offer of Nurse-Driven SARS-CoV2 Screening in Emergency Departments in the Paris Metropolitan Area

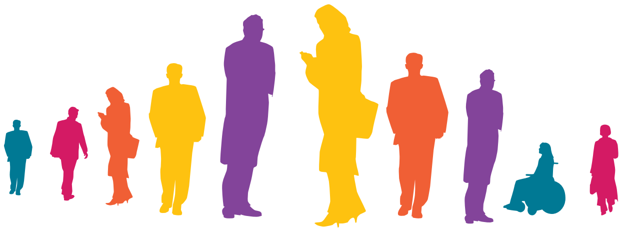

|__|__|__|__|__|__|
Date

N° DOSSIER :

|__|__|__| |__|__|__|__|__|
Center Patient

Sir, Madam,

A study is currently being conducted in this hospital to improve Covid-19 screening.
This infection linked to SARS-COV2 coronavirus can cause symptoms such as fever and respiratory symptoms but one can also be infected and show subtle signs such as a runny nose or a sore throat or even not show any signs of the disease. Yet, it is important to know it to avoid spreading the infection to others.

We are asking you to participate in this study carried out by the ANRS (Agence Nationale de Recherche sur le Sida et les Hépatites) and the AP-HP.

For this, we need to ask you several questions. The questionnaire is confidential. It will not be included in your medical file. Once completed, it will be rendered anonymous and will be used exclusively for the statistical analyses of data. You will give the completed questionnaire to the nurse who will take care of you. If you prefer, the nurse can help you fill out the questionnaire.

The nurse will offer you a free Covid-19 screening by a rapid test using a nasopharyngeal swab.
Your participation is voluntary. If you decide not to participate, it will have no consequence on the care that you receive in this department.

Thank you for your participation.

The AP-HP obtained favourable approval of this research project from the Committee for the Protection of Persons on 11/02/2021. The electronic file used for this research is implemented in conformity with the requirements of French (modified Database and Privacy law) and European regulations (RGPD: data protection regulation). You have the right to access and rectify any information in your file. You also have the right to oppose the transmission of your information covered by professional confidentiality used within this program. These rights apply to the health care professionals taking care of you. If you are encountering difficulties in the exercise of your rights, you may contact the Data Protection Officer of the AP-HP via email: protection.donnees.dsi@aphp.fr. This person will be able to explain the legal remedies available to you under the CNIL law. You may also exercise your right to object directly with the CNIL (www.cnil.fr). Once this project is completed and its data analysed, you can be informed of the global results by the intermediary of the research coordinator: J. LEBLANC, 01 49 28 22 02, DEPIST-COVID study, AP-HP

Coller ici l'étiquette patient

N° DOSSIER :

|__|__|__| |__|__|__|__|__|
Centre Patient

PLEASE FILL OUT THIS QUESTIONNAIRE BY CHECKING THE SQUARE CORRESPONDING TO YOUR RESPONSE ☒

1 You are: ☐ male ☐ female

2 What is your date of birth? |__|__| Month |__|__|__|__| Year

3 Do you have symptoms suggestive of Covid-19? ☐ Yes ☐ No

> **If yes, please precise:**

- ☐ Fever (>38°C) or fever sensation
- ☐ Unusual fatigue
- ☐ Headache
- ☐ Cough
- ☐ Breathing difficulty (shortness of breath)
- ☐ Sore throat
- ☐ Runny nose
- ☐ Loss of smell
- ☐ Loss of taste
- ☐ Diarrhoea
- ☐ Muscular pain
- ☐ Other

> **If yes, date of first symptoms:** |__|__| Day |__|__| Month |__|__|__|__| Year

4 What is your postcode? |__|__|__|__|__|

5 In which country were you born? ☐ France ☐ Other

Please specify your country of birth: ....

6 Until what age did you study? |__|__| years

7 Currently (over the last month): ☐ You work ☐ You do not work ☐ You are seeking employment ☐ You are a student

> **If you work:**

- **Do you work away from home?:** ☐ Yes ☐ No ☐ Partially
- **Does your occupation expose you to contacts with:**
  - Members of the public? ☐ Yes ☐ No
  - Sick persons? ☐ Yes ☐ No

8 What is your medical coverage:

- ☐ Social security without supplementary health insurance
- ☐ Social security with supplementary health insurance
- ☐ No medical coverage
- ☐ CMU (Universal medical cover)
- ☐ AME (State medical assistance)
- ☐ Other

9 Do you suffer from a chronic disease (diabetes, high blood pressure, angina pectoris, chronic bronchitis, asthma or chronic respiratory disease)? ☐ Yes ☐ No

10 Do you currently smoke cigarettes? ☐ Yes ☐ No

> **If yes, do you smoke:** ☐ Every day ☐ Occasionally

11 Do you live within a community (in a hostel or student halls for example)? ☐ Yes ☐ No

12 How many people do you currently live with? |__|__| people in total

13 Do you have children or students in your home? ☐ Yes ☐ No

OVER THE LAST 10 DAYS:

14 Apart from at home, have you always worn a mask? ☐ Yes ☐ No

15 Have you always worn a mask with friends or family that you do not live with? ☐ Yes ☐ No

16 Have you always kept within at least one meter in the following situations?

- At work or school: ☐ Yes ☐ No
- With friends or family: ☐ Yes ☐ No

17 Have you been in close contact with someone who has tested positive for Covid-19?

☐ Yes, 1-2 times ☐ Yes, 3 times or more ☐ No

> If yes, please precise:

☐ Family who you live with ☐ Family who you do not live with ☐ Friend

☐ Colleague ☐ Notification by the TousAntiCovid app ☐ Other

18 Have you been out of your home:

- For work? ☐ Yes, 1-2 times ☐ Yes, 3 times or more ☐ No
- For your studies? ☐ Yes, 1-2 times ☐ Yes, 3 times or more ☐ No

19 Have you used public transport? ☐ Yes, 1-2 times ☐ Yes, 3 times or more ☐ No

20 Have you been to a restaurant or a bar? ☐ Yes, 1-2 times ☐ Yes, 3 times or more ☐ No

21 Have you been to a gym/sports hall? ☐ Yes, 1-2 times ☐ Yes, 3 times or more ☐ No

22 Have you been to a gathering of more than 5 people?

☐ Yes, 1-2 times ☐ Yes, 3 times or more ☐ No

> If yes, for the busiest gathering:

- What type of gathering was it?

☐ For work ☐ At school/university ☐ A cultural site (cinema, theatre)

☐ A religious gathering ☐ A private gathering (friends/family) ☐ Other

- Was the gathering in: ☐ a Closed space ☐ An open space ☐ Both
- How many people were there: ☐ 5-10 ☐ 10-20 ☐ 20-50 ☐ > 50 people
- Were you wearing a mask? ☐ Yes ☐ No
  - > If yes: did you wear the mask the whole time? ☐ Yes ☐ No
- Were you more than one meter away from every person? ☐ Yes ☐ No

23 Have you been hospitalised? ☐ Yes, 1-2 times ☐ Yes, 3 times or more ☐ No

24 Have you taken a Covid-19 screening test? ☐ Yes, 1-2 times ☐ Yes, 3 times or more ☐ No

> If yes :

- Was it a: ☐ Nasopharyngeal swab ☐ Blood test (serology) ☐ Saliva swab
- Was the test? ☐ Positive ☐ Negative
  - > If positive: what was the date of the last positive test? |_|_| Day |_|_| Month |_|_|_|_| Year

25 How would you evaluate your risk of getting infected with Covid-19 compared to other people?

Risk: ☐ Very high ☐ High ☐ Similar ☐ Low ☐ Very low

PARTIE A REMPLIR PAR L'INFIRMIER(E)

Le patient accepte de participer à l'étude et de réaliser un test : ☐ Oui ☐ Non

26 **Test réalisé :** ☐ Oui ☐ Non

27 **Type de test :** ☐ RT-LAMP ☐ PCR multiplex

SI TEST NON RÉALISÉ

28 **Motifs** (Plusieurs choix possibles) :

☐ Refus :

- ☐ Déclare un test récent :
  date : |__|__| |__|__|__|__|
  Mois Année
- ☐ Déclare ne pas avoir de risque
- ☐ Patient vacciné contre la Covid-19
- ☐ Autre refus

☐ Consultant dans l'impossibilité de donner un accord

☐ Consultant parti

☐ Impossibilité liée au service

Commentaires :

Nom de l'infirmier(e) :

SI TEST RÉALISÉ

29 **Heure de lancement du test :** |__|__| h |__|__|

30 **Heure de lecture du test :** |__|__| h |__|__|

31 **Résultat du test SARS-CoV2 :**

☐ Négatif

☐ Positif

☐ Ininterprétable

> Refaire le test : ☐ Négatif ☐ Positif ☐ Ininterprétable

32 **Autres virus respiratoires (si applicable) :**

☐ Négatif

☐ Positif

> Précisez le virus

33 **Rendu du résultat au consultant :**

☐ Oui ☐ Non

> Si non : motif :

34 **Informations complémentaires :**

Test proposé par :

☐ Infirmier(e) ☐ Aide-soignant(e) ☐ Médecin

☐ Equipe de recherche

Test réalisé par :

☐ Infirmier(e) ☐ Aide-soignant(e) ☐ Médecin

☐ Equipe de recherche

POUR LE PERSONNEL DE RECHERCHE

ÉTUDE ANCILLAIRE : ☐ Oui ☐ Non

Test Antigénique :	☐ Oui ☐ Non	Résultat :	☐ Positif ☐ Négatif ☐ Ininterprétable
Milieu de transport :	☐ Oui ☐ Non	Résultat :	☐ Positif ☐ Négatif ☐ Ininterprétable

Si nécessaire, indiquer le numéro du questionnaire **Depist Covid** du patient **témoin :** ....

DEVENIR DU PATIENT : ☐ Ambulatoire ☐ Hospitalisé ☐ Soins intensifs

© DESIGN G. ALEXANDRE CHEYROU

Appendix B. Figure Geographical location of the 18 emergency departments involved in the DEPIST-COVID trial (Paris metropolitan area)

Fig B1. Geographical location of the 18 emergency departments involved in the DEPIST-COVID trial (Paris metropolitan area)

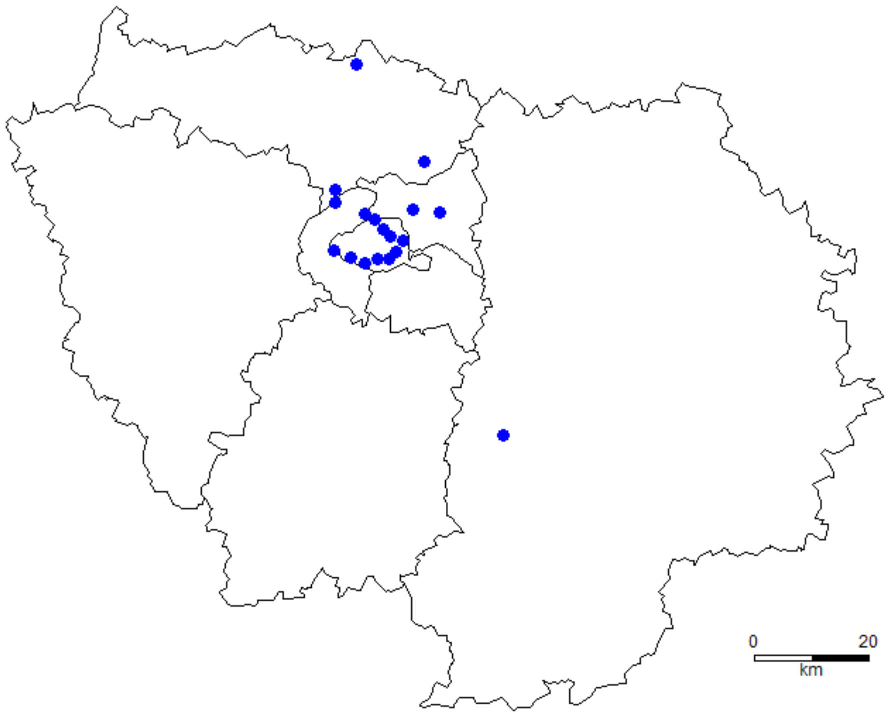

References:
R Core Team (2022). R: A language and environment for statistical computing. R Foundation for Statistical Computing, Vienna, Austria. URL https://www.R-project.org/.
Hijmans, R.J. (2022). raster: Geographic Data Analysis and Modeling. R package version 4.1.3. https://CRAN.R-project.org/package=raster.

Appendix C. Table Emergency department characteristics and study period duration

Table C1. Emergency department characteristics and study period duration

**ED center**	**1**	**2**	**3**	**4**	**5**	**6**	**7**	**8**	**9**	**10**	**11**	**12**	**13**	**14**	**15**	**16**	**17**	**18**
Pediatric care	0	0	0	0	1	0	1	0	1	0	1	1	0	0	0	1	0	0
University hospital	1	1	1	1	0	1	1	1	0	1	1	1	1	1	0	1	0	1
Inner Paris ED	1	1	1	0	0	0	0	1	0	0	0	0	1	1	0	0	1	1
Annual patient admissions in 2021[a]	74654	50891	57430	43990	91752	39977	96287	76855	53692	33083	59663	68957	58560	34630	26183	57635	62700	47550
SARS-CoV-2 device for rapid testing in routine care in the ED	1	1	1	1	0	1	0	1	0	0	0	0	1	1	0	0	1	0
Control study period (days)[b]	37	33	26	34	35	35	36	29	40	30	32	30	31	40	40	35	35	25
Intervention study period (days)[b]	37	33	26	34	35	35	36	29	38	30	32	30	31	40	40	35	35	30
Wash-out study period (days)[b]	1	2	1	1	2	1	2	1	2	1	2	1	2	1	1	2	7	2

Data are presented as numbers or No 0/Yes 1.

ED: Emergency department.

[a]Annual patient admissions included pediatric admissions.

[b]Duration of the intervention, control, and wash-out periods (days), median [first quartile, third quartile]: 34.5 [30.0, 36.0]; 34.5 [30.0, 36.0]; 1.5 [1.0, 2.0].

Appendix D. Table Characteristics of patients who underwent and declined SARS-CoV-2 screening for asymptomatic/paucisymptomatic patients during the intervention period

Table D1. Characteristics of patients who underwent and declined SARS-CoV-2 screening for asymptomatic/paucisymptomatic patients during the intervention period

	**Total population N=5 841***		**SARS-CoV-2 screening for asymptomatic / paucisymptomatic patients performed n=4 283**		**SARS-CoV-2 screening for asymptomatic / paucisymptomatic patients declined n=1 558**		**Risk difference**
	N	n (%) or median [Q1, Q3]	N	n (%) or median [Q1, Q3]	N	n (%) or median [Q1, Q3]	% or median (95% CI)
**Age (years)**	5771	40.0 [28.0, 55.0]	4257	41.0 [29.0, 56.0]	1514	37.0 [27.0, 54.0]	4.0 (2.6, 5.4)
**Sex**	5831		4278		1553		
Female		2696 (46.2)		1942 (45.4)		754 (48.6)	-3.2 (-6.1, -0.3)
**SARS-CoV-2 symptoms**	5610		4148		1462		
Yes		888 (15.8)		794 (19.1)		94 (6.4)	12.7 (10.9, 14.4)
**Symptom delay (days)**	578	3.0 [1.0, 7.0]	530	3.0 [1.0, 7.0]	48	4.0 [2.5, 11.0]	-1.0 (-3.4, 1.4)
**Country of birth**	5214		3945		1269		
France		3130 (60.0)		2338 (59.3)		792 (62.4)	-3.1 (-6.2, -0.05)
Other		2084 (40.0)		1607 (40.7)		477 (37.6)	3.1 (0.05, 6.2)
**Region of birth**	1853		1440		413		
Europe (outside France)		268 (14.5)		211 (14.7)		57 (13.8)	0.9 (-3.2, 4.4)
Americas outside the Caribbean		87 (4.7)		74 (5.1)		13 (3.1)	2.0 (-0.4, 3.8)
Asia-Middle East		224 (12.1)		180 (12.5)		44 (10.7)	1.8 (-1.9, 5.1)
France overseas and Caribbean		63 (3.4)		48 (3.3)		15 (3.6)	-0.3 (-2.7, 1.5)
Sub-Saharan Africa		474 (25.6)		369 (25.6)		105 (25.4)	0.2 (-4.7, 4.8)
North Africa		729 (39.3)		551 (38.3)		178 (43.1)	-4.8 (-10.3, 0.5)
Oceania		5 (0.3)		5 (0.3)		0 (0)	0.3 (-0.6, 0.8)
**Until what age did you study? (year)**	4528	20.0 [18.0, 24.0]	3431	20.0 [18.0, 24.0]	1097	20.0 [18.0, 24.0]	0.0 (-0.3, 0.3)
**Currently (within last month)**	5202		3925		1277		
You work		3034 (58.3)		2268 (57.8)		766 (60.0)	-2.2 (-5.3, 0.9)
You do not work		1414 (27.2)		1115 (28.4)		299 (23.4)	5.0 (2.2, 7.7)
You are searching for a job		368 (7.1)		257 (6.5)		111 (8.7)	-2.1 (-4.0, -0.5)
You are a student		321 (6.2)		233 (5.9)		88 (6.9)	-1.0 (-2.6, 0.5)
You are a student and you work		65 (1.2)		52 (1.3)		13 (1.0)	0.3 (-0.5, 0.9)
**Do you work outside your home?**	2949		2207		742		
Yes		2226 (75.5)		1663 (75.4)		563 (75.9)	-0.5 (-4.0, 3.1)
Partially		360 (12.2)		264 (12.0)		96 (12.9)	-1.0 (-3.9, 1.7)
**Do you have a profession that exposes you to contact with the public?**	2916		2188		728		
Yes		1898 (65.1)		1424 (65.1)		474 (65.1)	-0.03 (-4.0, 4.0)
**Medical coverage**	5166		3893		1273		
Social security with or without "mutuelle"		4205 (81.4)	.	3157 (81.1)	.	1048 (82.3)	-1.2 (-3.6, 1.3)

	**Total population N=5 841***		**SARS-CoV-2 screening for asymptomatic / paucisymptomatic patients performed n=4 283**		**SARS-CoV-2 screening for asymptomatic / paucisymptomatic patients declined n=1 558**		**Risk difference**
	N	n (%) or median [Q1, Q3]	N	n (%) or median [Q1, Q3]	N	n (%) or median [Q1, Q3]	% or median (95% CI)
CMU ('Couverture Médicale Universelle') or AME ('Aide Médicale d'Etat') or other		807 (15.6)	.	610 (15.7)	.	197 (15.5)	0.2 (-2.2, 2.4)
No coverage		154 (3.0)		126 (3.2)		28 (2.2)	1.0 (-0.04, 2.0)
**Follow-up for a chronic disease****	5092		3840		1252		
Yes		1198 (23.5)		960 (25.0)		238 (19.0)	6.0 (3.4, 8.5)
**Do you live in a community (foyer, student residence for example)?**	5047		3838		1209		
Yes		570 (11.3)		436 (11.4)		134 (11.1)	0.3 (-1.8, 2.2)
**How many people currently live with you in your home?**	5027	2.0 [1.0, 3.0]	3823	2.0 [1.0, 4.0]	1204	2.0 [1.0, 3.0]	0.0 (0.0, 0.0)
**Do you have children or students in your home?**	5131		3904		1227		
Yes		1941 (37.8)		1506 (38.6)		435 (35.5)	3.1 (0.01, 6.2)
**Have you ever had a screening test for COVID-19?**	5213		3877		1336		
Yes		3175 (60.9)	.	2263 (58.4)	.	912 (68.3)	-9.9 (-12.8, -6.9)
**Result**	2909		2142		767		
Positive		335 (11.5)		240 (11.2)		95 (12.4)	-1.2 (-4.0, 1.4)
**Time to prior positive test (days)**	308	114.5 [36.5, 183.5]	223	137.0 [56.0, 192.0]	85	55.0 [23.0, 157.0]	82.0 (46.2, 117.8)
**How do you rate your risk of being infected with COVID-19 compared to the average person?**	4931		3735		1196		
High or very high	.	834 (16.9)	.	664 (17.8)	.	170 (14.2)	3.6 (1.2, 5.8)
Identical	.	1664 (33.7)		1278 (34.2)		386 (32.3)	1.9 (-1.1, 5.0)
Low or very low	.	2433 (49.3)	.	1793 (48.0)	.	640 (53.5)	-5.5 (-8.7, -2.3)
**Emergency department discharge status*****	1793	.	1359	.	434	.	.
Hospitalization	.	24 (1.3)	.	24 (1.8)	.	0 (0)	1.8 (0.9, 2.6)
Only emergency department visit: outpatients	.	1769 (98.7)	.	1335 (98.2)	.	434 (100)	-1.8 (-2.6, -0.9)

** Patients who accepted the rapid test but did not have a test performed were not included in this table. In univariate analysis, factors associated with performing screening for asymptomatic/paucisymptomatic patients were the following: older age, male sex, having mild symptoms, not being from France, being unemployed, being followed up for a chronic disease, not being screened previously, and self-assessment of a high risk of infection.*

*** Chronic diseases: diabetes, arterial hypertension, angina pectoris, chronic bronchitis, asthma or chronic respiratory disease*

**** For this variable of the DEPIST-COVID questionnaire: data were collected in 8 centers, and it was not possible to collect them later in the remaining 10 centers. Findings are presented for 8 centers (n=2,226 patients, missing data: 19.5% in the 8 centers).*

Q1: First quartile; Q3: Third quartile; CI: Confidence interval.

Appendix E. Table Characteristics of patients tested through SARS-CoV-2 screening for asymptomatic/paucisymptomatic patients

Table E1. Characteristics of patients tested through SARS-CoV-2 screening for asymptomatic/paucisymptomatic patients

	**Total population N=4 192***		**New SARS-CoV-2 diagnoses n=224**		**Negative SARS-CoV-2 tests n=3 968**		**Risk difference**
	N	n (%) or median [Q1, Q3]	N	n (%) or median [Q1, Q3]	N	n (%) or median [Q1, Q3]	% or median (95% CI)
**Age (years)**	4166	41.0 [29.0, 56.0]	224	46.0 [31.0, 63.5]	3942	40.0 [29.0, 55.0]	6.0 (1.6, 10.4)
**Sex**	4187	.	224	.	3963	.	
Female	.	1900 (45.4)	.	97 (43.3)	.	1803 (45.5)	-2.2 (-8.7, 4.5)
**Test**	4192	.	224	.	3968	.	
Rapid molecular SARS-CoV-2 test	.	3611 (86.1)	.	108 (48.2)	.	3503 (88.3)	-40.1 (-46.6, -33.5)
Rapid multiplex respiratory virus test	.	581 (13.9)	.	116 (51.8)	.	465 (11.7)	40.1 (33.5, 46.6)
**SARS-CoV-2 symptoms**	4062	.	216	.	3846	.	
Yes**	.	766 (18.9)	.	133 (61.6)	.	633 (16.5)	45.1 (38.4, 51.5)
**Fever (>38°C) or feeling feverish**	754	.	130	.	624	.	
Yes	.	200 (26.5)	.	52 (40.0)	.	148 (23.7)	16.3 (7.5, 25.5)
**Unusual tiredness**	758	.	133	.	625	.	
Yes	.	328 (43.3)	.	64 (48.1)	.	264 (42.2)	5.9 (-3.3, 15.2)
**Headache**	759	.	132	.	627	.	
Yes	.	319 (42.0)	.	51 (38.6)	.	268 (42.7)	-4.1 (-12.9, 5.2)
**Cough**	756	.	131	.	625	.	
Yes	.	236 (31.2)	.	67 (51.1)	.	169 (27.0)	24.1 (14.9, 33.2)
**Breathing difficulty (shortness of breath)**	757	.	133	.	624	.	
Yes	.	213 (28.1)	.	37 (27.8)	.	176 (28.2)	-0.4 (-8.2, 8.4)
**Throat pain**	758	.	132	.	626	.	
Yes	.	157 (20.7)	.	30 (22.7)	.	127 (20.3)	2.4 (-4.7, 10.8)
**Common cold (runny nose)**	757	.	130	.	627	.	
Yes	.	160 (21.1)	.	19 (14.6)	.	141 (22.5)	-7.9 (-14.1, -0.2)
**Loss of smell**	755	.	131	.	624	.	
Yes	.	35 (4.6)	.	10 (7.6)	.	25 (4.0)	3.6 (-0.3, 9.6)
**Loss of taste**	756	.	132	.	624	.	
Yes	.	38 (5.0)	.	13 (9.8)	.	25 (4.0)	5.8 (1.4, 12.3)
**Diarrhoea**	758	.	132	.	626	.	
Yes	.	103 (13.6)	.	12 (9.1)	.	91 (14.5)	-5.4 (-10.4, 1.1)
**Muscle pain**	760	.	132	.	628	.	
Yes	.	223 (29.3)	.	46 (34.8)	.	177 (28.2)	6.7 (-1.8, 15.8)
**Others**	756	.	132	.	624	.	
Yes	.	67 (8.9)	.	8 (6.1)	.	59 (9.5)	-3.4 (-7.4, 2.4)
**Country of birth**	3865	.	193	.	3672	.	

	Total population N=4 192*		New SARS-CoV-2 diagnoses n=224		Negative SARS-CoV-2 tests n=3 968		Risk difference
	N	n (%) or median [Q1, Q3]	N	n (%) or median [Q1, Q3]	N	n (%) or median [Q1, Q3]	% or median (95% CI)
France	.	2290 (59.2)	.	88 (45.6)	.	2202 (60.0)	-14.4 (-21.4, -7.1)
Other	.	1575 (40.8)	.	105 (54.4)	.	1470 (40.0)	14.4 (7.1, 21.4)
**Region of birth**	1411	.	98	.	1313	.	
Europe (outside France)	.	207 (14.7)	.	10 (10.2)	.	197 (15.0)	-4.8 (-9.9, 3.0)
Americas outside the Caribbean	.	73 (5.2)	.	1 (1.0)	.	72 (5.5)	-4.5 (-6.2, 0.1)
Asia-Middle East	.	176 (12.5)	.	8 (8.2)	.	168 (12.8)	-4.6 (-9.1, 2.7)
France overseas and Caribbean	.	47 (3.3)	.	3 (3.1)	.	44 (3.4)	-0.3 (-2.6, 5.3)
Sub-Saharan Africa	.	364 (25.8)	.	39 (39.8)	.	325 (24.8)	15.0 (5.6, 25.2)
North Africa	.	538 (38.1)	.	37 (37.8)	.	501 (38.2)	-0.4 (-9.8, 9.8)
Oceania	.	5 (0.4)	.	0 (0)	.	5 (0.4)	-0.4 (-0.9, 3.4)
**Until what age did you study? (years)**	3363	20.0 [18.0, 24.0]	147	20.0 [17.0, 22.0]	3216	20.0 [18.0, 24.0]	0.0 (-0.6, 0.6)
**Currently (within last month)**	3845	.	194	.	3651	.	
You work	.	2218 (57.7)	.	102 (52.6)	.	2116 (58.0)	-5.4 (-12.6, 1.7)
You do not work	.	1097 (28.5)	.	71 (36.6)	.	1026 (28.1)	8.5 (1.9, 15.6)
You are searching for a job	.	250 (6.5)	.	9 (4.6)	.	241 (6.6)	-2.0 (-4.3, 2.0)
You are a student		229 (6.0)	.	6 (3.1)	.	223 (6.1)	-3.0 (-4.9, 0.5)
You are a student and you work	.	51 (1.3)	.	6 (3.1)	.	45 (1.2)	1.9 (0.1, 5.4)
**Do you work outside your home?**	2156	.	101	.	2055	.	
Yes	.	1622 (75.2)	.	83 (82.2)	.	1539 (74.9)	7.3 (-1.5, 13.9)
Partially	.	259 (12.0)	.	6 (5.9)	.	253 (12.3)	-6.4 (-10.0, 0.2)
**Do you have a profession that exposes you to contact with the public?**	2139	.	101	.	2038	.	
Yes	.	1393 (65.1)	.	68 (67.3)	.	1325 (65.0)	2.3 (-7.5, 11.0)
**Do you have a profession that exposes you to contact with sick people?**	1917	.	89	.	1828	.	
Yes	.	410 (21.4)	.	19 (21.3)	.	391 (21.4)	-0.04 (-7.6, 9.7)
**Medical coverage**	3814	.	185	.	3629	.	
Social security with or without "mutuelle"	.	3094 (81.1)	.	140 (75.7)	.	2954 (81.4)	-5.7 (-12.5, 0.05)
CMU (‘Couverture Médicale Universelle’) or AME (‘Aide Médicale d'Etat’) or other	.	595 (15.6)	.	33 (17.8)	.	562 (15.5)	2.4 (-2.7, 8.6)
No coverage	.	125 (3.3)	.	12 (6.5)	.	113 (3.1)	3.4 (0.6, 7.9)
**Follow-up for a chronic disease*** **	3762	.	192	.	3570	.	
Yes	.	940 (25.0)	.	61 (31.8)	.	879 (24.6)	7.1 (0.8, 14.2)

	Total population<br>N=4 192*		New SARS-CoV-2 diagnoses<br>n=224		Negative SARS-CoV-2 tests<br>n=3 968		Risk difference
	N	n (%)<br>or median [Q1, Q3]	N	n (%)<br>or median [Q1, Q3]	N	n (%)<br>or median [Q1, Q3]	%<br>or median<br>(95% CI)
**Do you live in a community (foyer, student residence for example)?**	3762	.	187	.	3575	.	
Yes	.	426 (11.3)	.	30 (16.0)	.	396 (11.1)	5.0 (0.3, 11.0)
**How many people currently live with you in your home?**	3745	2.0 [1.0, 4.0]	187	2.0 [1.0, 4.0]	3558	2.0 [1.0, 3.0]	0 (0.0, 1.0)
**Do you have children or students in your home?**	3824	.	191	.	3633	.	
Yes	.	1474 (38.5)	.	84 (44.0)	.	1390 (38.3)	5.7 (-1.3, 13.0)
**Have you been in contact with someone who tested positive in the last 10 days?**	3800	.	185	.	3615	.	
Yes	.	851 (22.4)	.	55 (29.7)	.	796 (22.0)	7.7 (1.4, 14.8)
**Have you ever had a screening test for COVID-19?**	3798	.	189	.	3609	.	
Yes	.	2196 (57.8)	.	80 (42.3)	.	2116 (58.6)	-16.3 (-23.3, -9.0)
**Time to prior positive test (days)**	198	148.0 [70.0, 204.0]	6	163.0 [103.0, 222.0]	192	148.0 [67.5, 202.0]	15.0 (*not defined*)
**How do you rate your risk of being COVID-19+?**	3657	.	176	.	3481	.	
High or very high	.	651 (17.8)	.	51 (29.0)	.	600 (17.2)	11.7 (5.4, 18.9)
Identical	.	1249 (34.2)	.	69 (39.2)	.	1180 (33.9)	5.3 (-1.8, 12.8)
Low or very low	.	1757 (48.0)	.	56 (31.8)	.	1701 (48.9)	-17.0 (-23.7, -9.7)
**Emergency department discharge status********	1328	.	36	.	1292	.	.
Hospitalization	.	24 (1.8)	.	2 (5.6)	.	22 (1.7)	3.9 (-0.3, 16.5)
Only emergency department visit: outpatients	.	1304 (98.2)	.	34 (94.4)	.	1270 (98.3)	-3.9 (-16.5, 0.3)
**Among patients with rapid multiplex respiratory virus test, other respiratory viruses**	581	.	116	.	465	.	.
Yes*****	.	77 (13.3)	.	5 (4.3)	.	72 (15.5)	-11.2 (-15.7, -5.2)

** Patients with indeterminate results or a known positive SARS-CoV-2 status were not included in this table. In univariate analysis, factors associated with new infections diagnosed through screening for asymptomatic/paucisymptomatic patients were mainly older age, having mild symptoms, not being from France, particularly being from Sub-Saharan Africa, being unemployed, not having medical coverage, living in a community, being a case contact and not being previously screened.*

*** Among patients who underwent a rapid molecular SARS-CoV-2 test, 258 (7.4%) reported having symptoms. Among patients who underwent a rapid multiplex respiratory virus test, 508 (90.1%) reported having symptoms.*

**** Chronic diseases: diabetes, arterial hypertension, angina pectoris, chronic bronchitis, asthma or chronic respiratory disease*

***** For this variable of the DEPIST-COVID questionnaire, data were collected in 8 centers, and it was not possible to collect them later in the remaining 10 centers. Findings are presented for 8 centers (n=1,510 patients, missing data: 12.1% in the 8 centers).*

****** Rhinovirus/Enterovirus: n=29 (37.7%), Coronavirus OC43: n=12 (15.6%), Coronavirus NL63: n=10 (13.0%), Human Metapneumovirus A+B: n=10 (13.0%), Respiratory Syncytial Virus A+B: n=6 (7.8%), Parainfluenza virus 3: n=3 (3.9%), Bocavirus: n=2 (2.6%), Parainfluenza virus 4: n=2 (2.6%), Bocavirus | Coronavirus HKU1: n=1 (1.3%), Coronavirus 229E: n=1 (1.3%), Influenza A: n=1 (1.3%). Among SARS-CoV-2+ patients: Rhinovirus/Enterovirus: n=2, Bocavirus: n=1, Parainfluenza virus 4: n=1, Respiratory Syncytial Virus A+B: n=1.*

Q1: First quartile; Q3: Third quartile; CI: Confidence interval.

Appendix F. Figure Newly diagnosed SARS-CoV-2-positive patients per emergency department and strategy

Fig F1. Newly diagnosed SARS-CoV-2-positive patients per emergency department and strategy

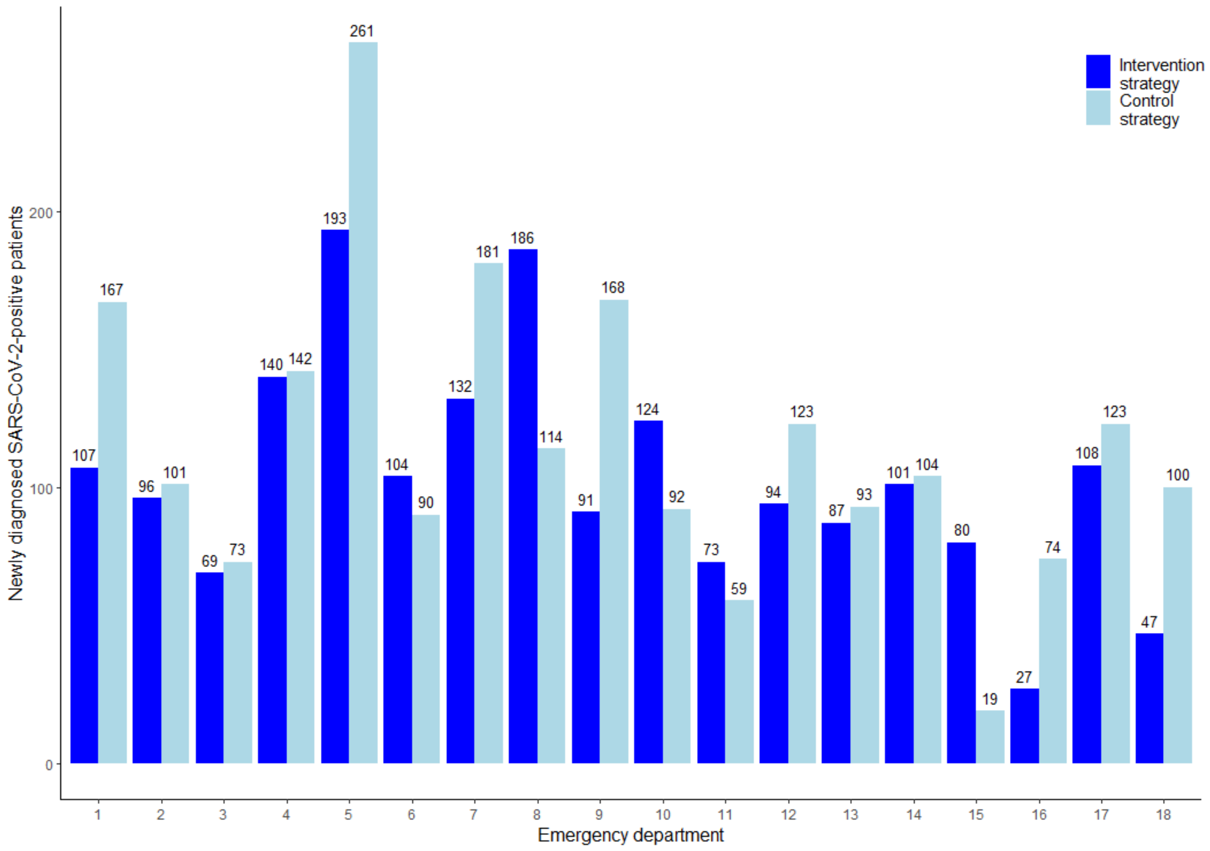

Appendix G. Figure SARS-CoV-2 incidence rate in the Paris metropolitan area and per geographical department (75, 77, 92, 93, 95) of the emergency departments involved in the study

Fig G1. SARS-CoV-2 incidence rate in the Paris metropolitan area and per geographical department (75, 77, 92, 93, 95) of the emergency departments involved in the study

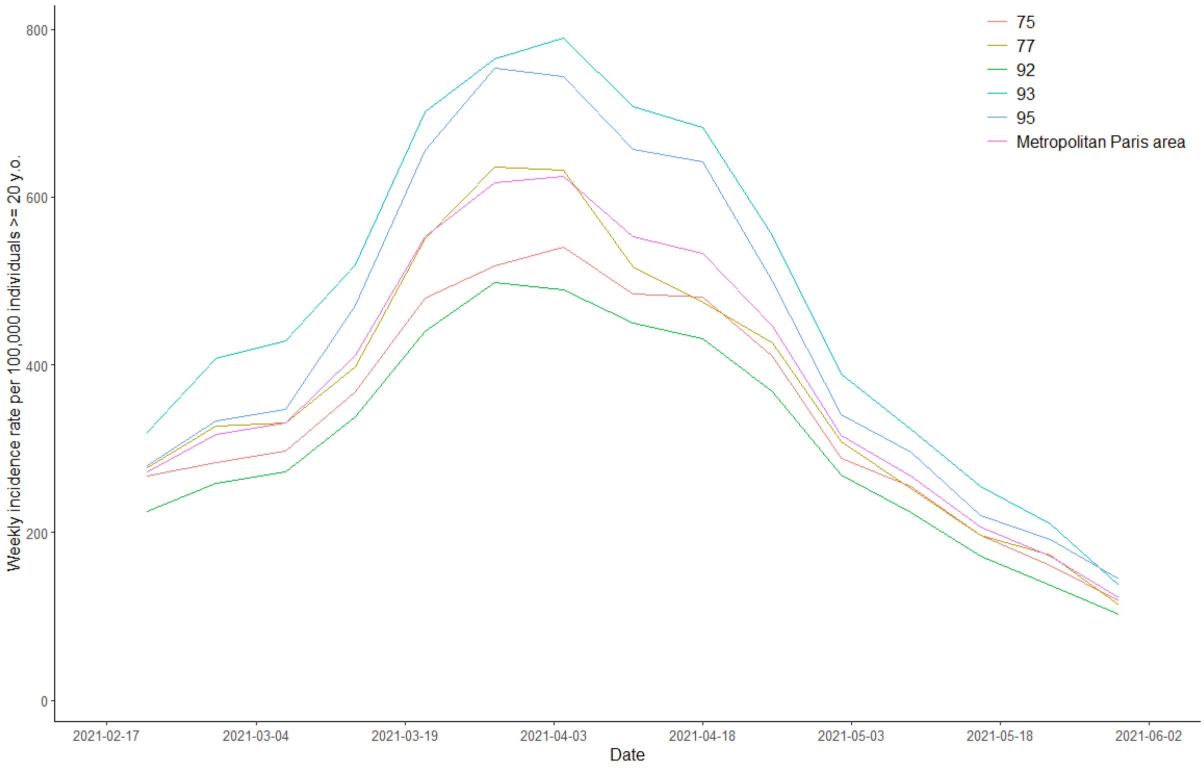

y.o.: years old

Appendix H. Table Characteristics of patients newly diagnosed with SARS-CoV-2 infection

Table H1. Characteristics of patients newly diagnosed with SARS-CoV-2 infection

	**Total population N=3 943**		**Control period n=2 084**		**Intervention period n=1 859**		**Risk difference**
	N	n (%) or median [Q1, Q3]	n	n (%) or median [Q1, Q3]	n	n (%) or median [Q1, Q3]	% or median (95% CI)
**Age (years)**	3943	59.7 [41.0, 75.0]	2084	61.0 [41.4, 77.0]	1859	58.0 [40.0, 74.0]	3.0 (1.0, 5.0)
**Sex**	3943		2084		1859		
Female		1845 (46.8)		947 (45.4)		898 (48.3)	-2.9 (-6.0, 0.3)
**Center**	3943		2084		1859		
1		274 (6.9)		167 (8.0)		107 (5.8)	2.3 (0.7, 3.8)
2		197 (5.0)		101 (4.8)		96 (5.2)	-0.3 (-1.7, 1.0)
3		142 (3.6)		73 (3.5)		69 (3.7)	-0.2 (-1.4, 1.0)
4		282 (7.2)		142 (6.8)		140 (7.5)	-0.7 (-2.4, 0.9)
5		454 (11.5)		261 (12.5)		193 (10.4)	2.1 (0.1, 4.1)
6		194 (4.9)		90 (4.3)		104 (5.6)	-1.3 (-2.7, 0.08)
7		313 (7.9)		181 (8.7)		132 (7.1)	1.6 (-0.1, 3.3)
8		300 (7.6)		114 (5.5)		186 (10.0)	-4.5 (-6.2, -2.9)
9		259 (6.6)		168 (8.1)		91 (4.9)	3.2 (1.6, 4.7)
10		216 (5.5)		92 (4.4)		124 (6.7)	-2.3 (-3.7, -0.8)
11		132 (3.3)		59 (2.8)		73 (3.9)	-1.1 (-2.3, 0.03)
12		217 (5.5)		123 (5.9)		94 (5.1)	0.8 (-0.6, 2.3)
13		180 (4.6)		93 (4.5)		87 (4.7)	-0.2 (-1.5, 1.1)
14		205 (5.2)		104 (5.0)		101 (5.4)	-0.4 (-1.9, 0.9)
15		99 (2.5)		19 (0.9)		80 (4.3)	-3.4 (-4.5, -2.4)
16		101 (2.6)		74 (3.6)		27 (1.5)	2.1 (1.1, 3.1)
17		231 (5.9)		123 (5.9)		108 (5.8)	0.09 (-1.4, 1.6)
18		147 (3.7)		100 (4.8)		47 (2.5)	2.3 (1.1, 3.5)

Q1: First quartile; Q3: Third quartile; CI: Confidence interval.

Appendix I. Primary outcome modeling, sensitivity analyses, and intercluster and intracluster correlation coefficients

1. Final model

The following models were compared using likelihood ratio tests:

a. A model including the center as a random intercept, the center-by-weekly period interaction as a random effect, as well as the strategy (intervention *vs.* control) and the weekly period as fixed effects.
b. A model including the same variables (Model a) and the community incidence rate for SARS-CoV-2 as a fixed effect.
c. A model including the same variables (Model b) and the community incidence rate-by-weekly period interaction.

The selected model was **Model c**.

Table I1. Coefficient estimates from the generalized linear mixed model with a Poisson distribution including the center as a random intercept, the center-by-weekly period interaction as a random effect and the strategy, weekly period and community incidence rate-by-weekly period interaction as fixed effects (SAS PROC Glimmix)

**Variable**	**Coefficient**	**Standard error**	**t test**	***p* value**	**Relative risk**	**(95% Confidence interval) relative risk**
Strategy	0.02	0.04	0.48	0.63	1.02	(0.94 to 1.11)
Weekly period	-0.09	0.02	-4.45	<.0001		
Community incidence rate	0.00	0.00	-0.75	0.46		
Community incidence rate-by-weekly period interaction	0.00	0.00	5.33	<.0001		

**Covariance**	**Coefficient**	**Standard error**
Center	0.07	0.03
Center-by-period interaction	0.00	0.00

The variability among centers was estimated as small (0.07) compared to the associated standard error (0.03).

The ratio of the 'Generalized Chi-Square statistic' and its degrees of freedom was 1.17 and close to 1. The scaled Pearson statistic for the conditional distribution was similar (1.17). These arguments are not in favour of an overdispersion of the data.

Significant weekly period effect ($p<0.0001$) and community incidence rate-by-weekly period interaction ($p<0.0001$) were identified.

Table I2. Significant random effects

Variable	Center	Coefficient	Standard error	t test	*p* value
Intercept	Center 1	-0.36	0.14	-2.61	0.01
Center-by-period interaction	Center 11	0.05	0.02	2.48	0.02
Intercept	Center 13	0.30	0.15	2.02	0.05
Intercept	Center 5	0.31	0.14	2.17	0.04
Intercept	Center 6	0.33	0.13	2.59	0.01
Intercept	Center 8	-0.37	0.14	-2.72	0.01

According to the random parameters of the final model, the intracluster variability was not negligible. Significant differences were observed in 5 centers. Given the multiplicity of the tests, the interpretation is limited.

2. Sensitivity analyses

Table I3. Analysis considering a binarized period (0/1)

Variable	Coefficient	Standard error	t test	*p* value	Relative risk	(95% Confidence interval) relative risk
Intervention	-0.02	0.07	-0.25	0.80	0.98	(0.84 to 1.15)

Covariance	Coefficient	Standard error
Center	0.08	0.04
Center-by-period interaction	0.03	0.02

Table I4. Analysis including variables used for randomization (screening equipment in emergency departments and flow in emergency departments)

Variable	Coefficient	Standard error	t test	*p* value	Relative risk	(95% Confidence interval) relative risk
Intervention	0.02	0.04	0.53	0.60	1.02	(0.94 to 1.11)

Covariance	Coefficient	Standard error
Center	0.06	0.03
Center-by-period interaction	0.00	0.00

Table I5. Analysis including strategy * week_since_roll_out interaction

Variable	Coefficient	Standard error	t test	*p* value	Relative risk	(95% Confidence interval) relative risk
Intervention	-0.02	0.07	-0.28	0.78	0.98	(0.85 to 1.13)

Covariance	Coefficient	Standard error
Center	0.07	0.03
Center-by-period interaction	0.00	0.00

Table I6. Analysis at the center level

Model	Coefficient	Standard error	t test	*p* value	Relative risk	(95% Confidence interval) relative risk	
Final model	0.02	0.04	0.48	0.63	1.02	0.94	1.11
Model without center 1	0.04	0.04	0.92	0.36	1.04	0.96	1.14
Model without center 2	0.02	0.04	0.39	0.70	1.02	0.93	1.11
Model without center 3	0.02	0.04	0.52	0.60	1.02	0.94	1.11
Model without center 4	0.01	0.04	0.23	0.82	1.01	0.93	1.10
Model without center 5	0.00	0.04	-0.05	0.96	1.00	0.91	1.09
Model without center 6	0.04	0.04	0.81	0.42	1.04	0.95	1.13
Model without center 7	0.01	0.04	0.29	0.77	1.01	0.93	1.10
Model without center 8	-0.01	0.05	-0.25	0.80	0.99	0.90	1.08
Model without center 9	0.04	0.04	0.82	0.41	1.04	0.95	1.13
Model without center 10	0.02	0.04	0.38	0.71	1.02	0.93	1.11
Model without center 11	0.03	0.04	0.71	0.48	1.03	0.95	1.13
Model without center 12	0.02	0.04	0.44	0.66	1.02	0.94	1.11
Model without center 13	0.03	0.04	0.68	0.50	1.03	0.94	1.12
Model without center 14	0.02	0.04	0.46	0.64	1.02	0.93	1.12
Model without center 15	0.00	0.04	0.02	0.98	1.00	0.92	1.09
Model without center 16	0.03	0.04	0.69	0.49	1.03	0.95	1.12
Model without center 17	0.02	0.04	0.42	0.67	1.02	0.93	1.11
Model without center 18	0.03	0.04	0.76	0.45	1.03	0.95	1.13

A sensitivity analysis was performed at the center level, successively excluding one center from the sample and estimating the parameters of the 18 samples. No center influenced the estimation of the strategy fixed parameter of the model; the response to the intervention did not vary.

3. Intercluster and intracluster correlation coefficients

The intercluster correlation coefficient ρ_{12} and intracluster correlation coefficient ρ were calculated *a posteriori* following the Donner formula [1]. Small correlation values were found:

$\rho_{12} = 0.0012$

$\rho = 0.0035$

The basic assumption for the final model (the intercluster correlation was zero) was supported by the data.

Reference

1. Donner A, Klar N, Zou G. Methods for the statistical analysis of binary data in split-cluster designs. Biometrics. 2004;60(4):919-25. Epub 2004/12/21. doi: 10.1111/j.0006-341X.2004.00247.x. PubMed PMID: 15606412.

Appendix J. Comparison of the proportions of new SARS-CoV-2 diagnoses through screening in emergency departments and of positive tests through community screening for individuals aged 18+ of the geographical departments of the Paris metropolitan area screened during the same period

a. Table J1. New SARS-CoV-2 infections diagnosed through screening in emergency departments for asymptomatic/paucisymptomatic patients and positive tests through community screening for asymptomatic adults in the geographical departments of the Paris metropolitan area during the same period

**SARS-CoV-2 positive tests**	**Total population**		**SARS-CoV-2 screening for asymptomatic / paucisymptomatic patients in EDs**		**Community screening for SARS-CoV-2 in the Paris metropolitan area***		**Risk difference (95% Confidence interval)**
	N	n (%)	n	n (%)	n	n (%)	
	3404867	.	4283	.	3400584	.	.
Yes	.	156522 (4.6)	.	224 (5.2)	.	156298 (4.6)	0.6 (0.01, 1.3)
No	.	3248345 (95.4)	.	4059 (94.8)	.	3244286 (95.4)	-0.6 (-1.3, -0.01)

** Source: Community screening data were provided by Santé publique France on May 20, 2022.*

b. Table J2. New SARS-CoV-2 infections diagnosed through screening in emergency departments (EDs) for asymptomatic/paucisymptomatic patients per ED and positive tests through community screening for asymptomatic adults tested in the corresponding geographical department

**SARS-CoV-2 positive tests**	**Total population**		**SARS-CoV-2 screening for asymptomatic / paucisymptomatic patients in EDs**		**Community screening for SARS-CoV-2 in the geographical department***		**Risk difference (95% Confidence interval)**
	N	n (%)	n	n (%)	n	n (%)	
Center 1	453511		429		453082		
		15782 (3.5)		23 (5.4)		15759 (3.5)	**1.9 (0.1, 4.4)**
Center 2	414301		357		413944		
		14778 (3.6)		10 (2.8)		14768 (3.6)	-0.8 (-2.0, 1.5)
Center 3	346384		129		346255		
		12805 (3.7)		2 (1.6)		12803 (3.7)	-2.1 (-3.3, 1.8)
Center 4	426629		176		426453		
		15235 (3.6)		11 (6.3)		15224 (3.6)	2.7 (-0.05, 7.3)
Center 5	130485		269		130216		
		8270 (6.3)		14 (5.2)		8256 (6.3)	-1.1 (-3.2, 2.2)
Center 6	203354		201		203153		
		17124 (8.4)		13 (6.5)		17111 (8.4)	-2.0 (-4.6, 2.3)
Center 7	140284		322		139962		
		8993 (6.4)		15 (4.7)		8978 (6.4)	-1.8 (-3.6, 1.1)
Center 8	401022		149		400873		

SARS-CoV-2 positive tests	Total population		SARS-CoV-2 screening for asymptomatic / paucisymptomatic patients in EDs		Community screening for SARS-CoV-2 in the geographical department*		Risk difference (95% Confidence interval)
	N	n (%)	n	n (%)	n	n (%)	
		15514 (3.9)		4 (2.7)		15510 (3.9)	-1.2 (-2.8, 2.8)
Center 9	164729		345		164384		
		7660 (4.7)		19 (5.5)		7641 (4.6)	0.9 (-1.1, 3.8)
Center 10	196232		152		196080		
		9869 (5.0)		7 (4.6)		9862 (5.0)	-0.4 (-2.8, 4.2)
Center 11	219037		198		218839		
		18140 (8.3)		23 (11.6)		18117 (8.3)	3.3 (-0.4, 8.5)
Centere 12	191265		136		191129		
		8740 (4.6)		11 (8.1)		8729 (4.6)	**3.5 (0.008, 9.3)**
Center 13	454731		173		454558		
		16666 (3.7)		5 (2.9)		16661 (3.7)	-0.8 (-2.4, 2.9)
Center 14	508063		281		507782		
		16050 (3.2)		10 (3.6)		16040 (3.2)	0.4 (-1.2, 3.3)
Center 15	180978		331		180647		
		13634 (7.5)		49 (14.8)		13585 (7.5)	**7.3 (3.9, 11.5)**
Center 16	199805		353		199452		
		5243 (2.6)		2 (0.6)		5241 (2.6)	**-2.1 (-2.5, -0.6)**
Center 17	441912		147		441765		
		13321 (3.0)		2 (1.4)		13319 (3.0)	-1.7 (-2.6, 1.8)
Center 18	383530		135		383395		
		7680 (2.0)		4 (3.0)		7676 (2.0)	1.0 (-0.8, 5.4)

** Source: Community screening data were provided by Santé publique France on May 20, 2022.*

For each emergency department (ED) taken separately, the proportion of new infections diagnosed through ED screening for asymptomatic/paucisymptomatic patients was higher for 3 EDs compared to the proportion of positive tests among asymptomatic individuals tested in the corresponding geographical department.

c. Table J3. New SARS-CoV-2 diagnoses through screening in emergency departments and positive tests through community screening for adults in the geographical departments of the Paris metropolitan area during the same period

SARS-CoV-2 positive tests	Total population		SARS-CoV-2 screening in EDs		Community screening for SARS-CoV-2 in the Paris metropolitan area*		Risk difference (95% Confidence interval)
	N	n (%)	N	n (%)	N	n (%)	
	4720858	.	40367	.	4680491	.	
Yes	.	365032 (7.7)	.	3943 (9.8)	.	361089 (7.7)	2.1 (1.8, 2.3)
No	.	4355826 (92.3)	.	36424 (90.2)	.	4319402 (92.3)	-2.1 (-2.3, -1.8)

** Source: Community screening data were provided by Santé publique France on May 20, 2022.*

Appendix K. Figures Visits in the 18 emergency departments during the study period in 2021 and during the same period in 2019

a. Fig K1. Visits to the 18 emergency departments during the same period in 2019 and 2021

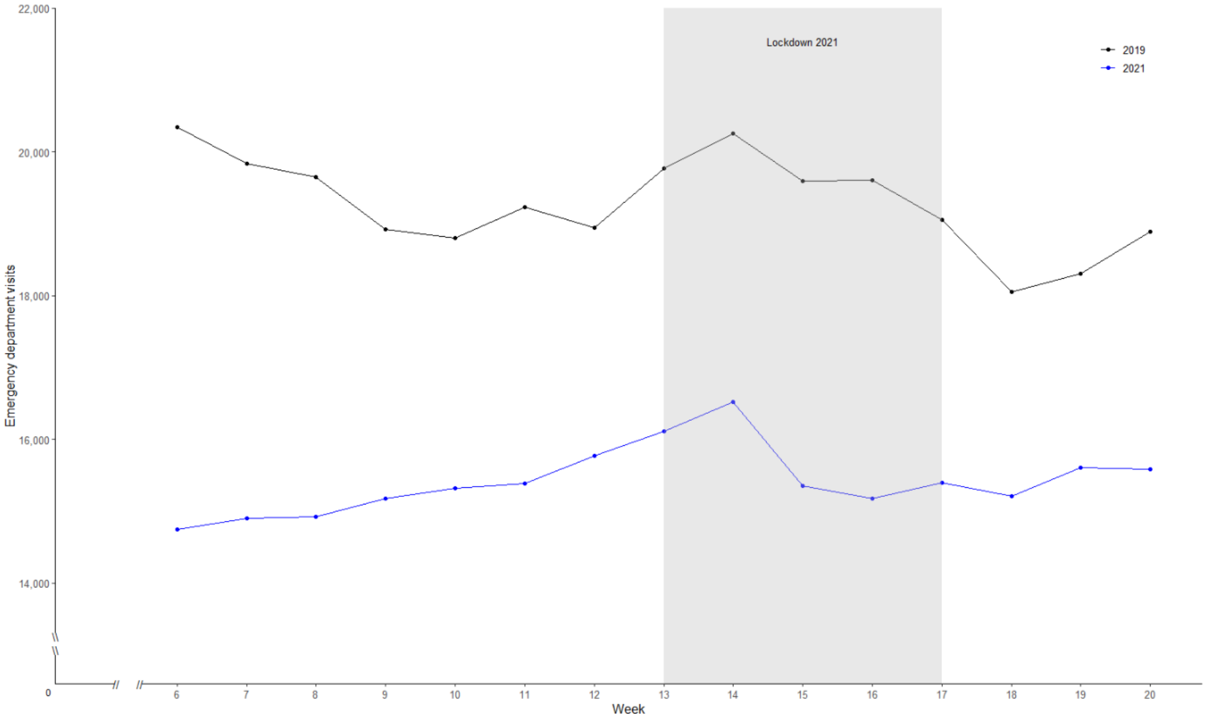

Source: Observatoire Régional des Soins Non Programmés (ORNSP). Activité des services d'urgences en Ile-de-France 2021 [Available from: https://orsnp-idf.fr/wp-content/uploads/2022/06/20220606_rapport_annuel_urgences_2021_VF.pdf. Published: June 2022. Accessed date: 07/06/2023

b. Fig K2. Proportion of emergency department visits without hospital admission per week in the 18 emergency departments during the same period in 2019 and in 2021

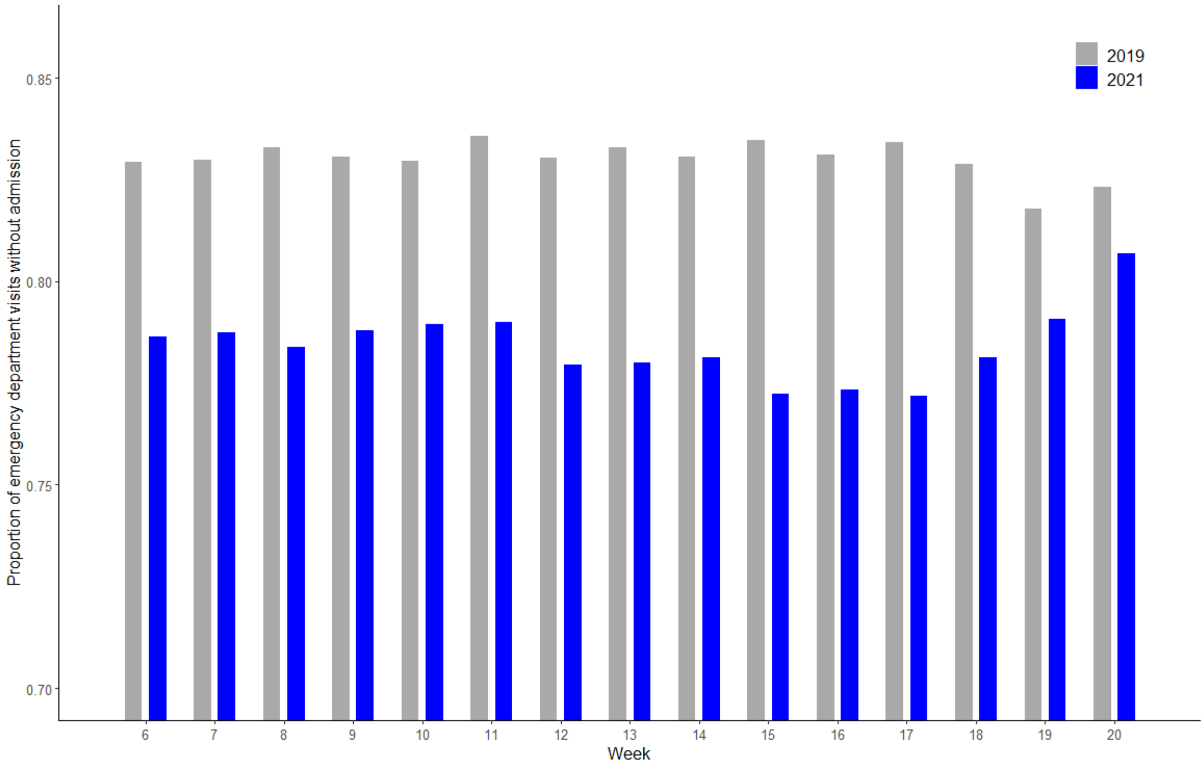

Source: Observatoire Régional des Soins Non Programmés (ORNSP). Activité des services d'urgences en Ile-de-France 2021 [Available from: https://orsnp-idf.fr/wp-content/uploads/2022/06/20220606_rapport_annuel_urgences_2021_VF.pdf. Published: June 2022. Accessed date: 07/06/2023

Appendix L. Cluster randomised trials extension of the Consolidated Standards of Reporting Trials (CONSORT) checklist

Table 1: CONSORT 2010 checklist of information to include when reporting a cluster randomised trial

Section/Topic	Item No	Standard Checklist item	Extension for cluster designs	Paragraph numbers per section
**Title and abstract**				
	1a	Identification as a randomised trial in the title	Identification as a cluster randomised trial in the title	Title
	1b	Structured summary of trial design, methods, results, and conclusions (for specific guidance see CONSORT for abstracts)[1,2]	See table 2	Abstract, paragraphs 1-4
**Introduction**				
**Background and objectives**	2a	Scientific background and explanation of rationale	Rationale for using a cluster design	Introduction, paragraphs 1-5<br><br>Discussion, paragraph 9
	2b	Specific objectives or hypotheses	Whether objectives pertain to the cluster level, the individual participant level or both	Introduction, paragraph 5<br><br>Methods, paragraph 18-20
**Methods**				
**Trial design**	3a	Description of trial design (such as parallel, factorial) including allocation ratio	Definition of cluster and description of how the design features apply to the clusters	Methods, paragraphs 1, 3, 5, 6
	3b	Important changes to methods after trial commencement (such as eligibility criteria), with reasons		Not applicable
**Participants**	4a	Eligibility criteria for participants	Eligibility criteria for clusters	Methods, paragraphs 3,4
	4b	Settings and locations where the data were collected		Methods, paragraph 3
**Interventions**	5	The interventions for each group with sufficient details to allow replication, including how and when	Whether interventions pertain to the cluster level, the individual participant level or both	Methods, paragraphs 8-16<br><br>Figure 3

		they were actually administered		S3 Appendix
**Outcomes**	6a	Completely defined pre-specified primary and secondary outcome measures, including how and when they were assessed	Whether outcome measures pertain to the cluster level, the individual participant level or both	Methods, paragraphs 18-20
	6b	Any changes to trial outcomes after the trial commenced, with reasons		S1 appendix (final statistical analysis plan and a summary of the changes)
**Sample size**	7a	How sample size was determined	Method of calculation, number of clusters(s) (and whether equal or unequal cluster sizes are assumed), cluster size, a coefficient of intracluster correlation (ICC or *k*), and an indication of its uncertainty	Methods, paragraph 21
	7b	When applicable, explanation of any interim analyses and stopping guidelines		Not applicable
**Randomisation:**				
**Sequence generation**	8a	Method used to generate the random allocation sequence		Methods, paragraph 5
	8b	Type of randomisation; details of any restriction (such as blocking and block size)	Details of stratification or matching if used	Methods, paragraph 5
**Allocation concealment mechanism**	9	Mechanism used to implement the random allocation sequence (such as sequentially numbered containers), describing any steps taken to conceal the sequence until interventions were assigned	Specification that allocation was based on clusters rather than individuals and whether allocation concealment (if any) was at the cluster level, the individual participant level or both	Methods, paragraph 5
**Implementation**	10	Who generated the random allocation sequence, who enrolled	Replace by 10a, 10b and 10c	Methods, paragraph 5

		participants, and who assigned participants to interventions		
	10a		Who generated the random allocation sequence, who enrolled clusters, and who assigned clusters to interventions	Methods, paragraph 5
	10b		Mechanism by which individual participants were included in clusters for the purposes of the trial (such as complete enumeration, random sampling)	Methods, paragraphs 4-5
	10c		From whom consent was sought (representatives of the cluster, or individual cluster members, or both), and whether consent was sought before or after randomisation	Methods, paragraphs 3-4
**Blinding**	11a	If done, who was blinded after assignment to interventions (for example, participants, care providers, those assessing outcomes) and how		Methods, paragraph 7
	11b	If relevant, description of the similarity of interventions		Not applicable
**Statistical methods**	12a	Statistical methods used to compare groups for primary and secondary outcomes	How clustering was taken into account	Methods, paragraphs 22-33
	12b	Methods for additional analyses, such as subgroup analyses and adjusted analyses		Not applicable
**Results**				
**Participant flow (a diagram is strongly recommended)**	13a	For each group, the numbers of participants who were randomly assigned, received intended treatment, and were analysed for the	For each group, the numbers of clusters that were randomly assigned, received intended treatment, and were analysed for the primary outcome	Results, paragraphs 1-7<br>Figure 1

		primary outcome		
	13b	For each group, losses and exclusions after randomisation, together with reasons	For each group, losses and exclusions for both clusters and individual cluster members	Not applicable
**Recruitment**	14a	Dates defining the periods of recruitment and follow-up		Abstract, paragraph 2<br><br>Methods, paragraph 1<br><br>Figure 3
	14b	Why the trial ended or was stopped		Not applicable
**Baseline data**	15	A table showing baseline demographic and clinical characteristics for each group	Baseline characteristics for the individual and cluster levels as applicable for each group	Table 1
**Numbers analysed**	16	For each group, number of participants (denominator) included in each analysis and whether the analysis was by original assigned groups	For each group, number of clusters included in each analysis	Results, paragraphs 1-7<br><br>Figure 2
**Outcomes and estimation**	17a	For each primary and secondary outcome, results for each group, and the estimated effect size and its precision (such as 95% confidence interval)	Results at the individual or cluster level as applicable and a coefficient of intracluster correlation (ICC or k) for each primary outcome	Results, paragraphs 7-12<br><br>S4, S5, S9, S10 appendixes
	17b	For binary outcomes, presentation of both absolute and relative effect sizes is recommended		Results, paragraphs 7-12<br><br>S4, S5, S9, S10 appendixes
**Ancillary analyses**	18	Results of any other analyses performed, including subgroup analyses and adjusted analyses, distinguishing pre-specified from exploratory		Not applicable
**Harms**	19	All important harms or unintended effects in each group (for specific guidance see CONSORT for harms[3])		Not applicable

**Discussion**				Discussion, paragraphs 1-16
**Limitations**	20	Trial limitations, addressing sources of potential bias, imprecision, and, if relevant, multiplicity of analyses		Discussion, paragraphs 11-13
**Generalisability**	21	Generalisability (external validity, applicability) of the trial findings	Generalisability to clusters and/or individual participants (as relevant)	Discussion, paragraphs 1-16
**Interpretation**	22	Interpretation consistent with results, balancing benefits and harms, and considering other relevant evidence		Discussion, paragraphs 1-16
**Other information**				
**Registration**	23	Registration number and name of trial registry		Methods, paragraph 2
**Protocol**	24	Where the full trial protocol can be accessed, if available		S1 appendix
**Funding**	25	Sources of funding and other support (such as supply of drugs), role of funders		Title page

** Note: page numbers optional depending on journal requirements*

REFERENCES

[1] Hopewell S, Clarke M, Moher D, Wager E, Middleton P, Altman DG, et al. CONSORT for reporting randomised trials in journal and conference abstracts. *Lancet* 2008, 371:281-283

[2] Hopewell S, Clarke M, Moher D, Wager E, Middleton P, Altman DG at al (2008) CONSORT for reporting randomized controlled trials in journal and conference abstracts: explanation and elaboration. *PLoS Med* 5(1): e20

[3] Ioannidis JP, Evans SJ, Gotzsche PC, O'Neill RT, Altman DG, Schulz K, Moher D. Better reporting of harms in randomized trials: an extension of the CONSORT statement. *Ann Intern Med* 2004; 141(10):781-788.

Appendix M. Study group

The study was performed on behalf of the DEPIST-COVID group, which includes the investigators who led the data collection (listed below [all in France], emergency department/virology department), and on behalf of the FHU IMPEC group, as well as Flore-Anne de Baudinière involved in the study's conception and in the implementation and monitoring of the trial; Dominique Pateron, Marine Cachanado, Dominique Damas, Alexandra Rousseau who were involved in the study's conception and follow-up; Johanna Kalsch, Violaine Baron, Malick Cissé, Charlotte Cossé-Barthomier, Clara Grelaud, Marie Vayssettes, Léopoldine Legrain, Benjamin Laverdant, Nina Temam, Clara Duran, Pierrick Abouquir who were involved in the follow-up of the trial.

Hôpital Lariboisière, Assistance Publique–Hôpitaux de Paris (AP-HP): Xavier Eyer, Charlotte Attali, Chloé Maygnan-Guerin / Hervé Jacquier.
Hôpital Cochin, AP-HP: Hélène Roland, Florence Dumas / Jean-François Méritet.
Hôpital Ambroise Paré, AP-HP: Amandine Calafat, Abdeslame Boughrara, Sébastien Beaune / Elyanne Gault.
Centre Hospitalier de Melun: Alain-Gil Mpela / Aurélia Pitsch, Marie Picque.
Groupement Hospitalier de Territoire Nord-Ouest Vexin Val-d'Oise: Alexandra Stéphane, Chantal Huynh-Ba, Agnès Ricard-Hibon / Pascale Martres.
Centre Hospitalier d'Argenteuil: Valérie Torck, Catherine Le Gall / Laurence Courdavault.
Hôpital Saint Louis, AP-HP: Maud Anastassiou, Anne Pouessel, Olivier Peyrony, Jean-Paul Fontaine / Linda Feghoul, Nadia Mahjoub.
Centre Hospitalier de Gonesse: Soraya Mokhtari, Mustapha Youssef / Wacila Berkani.
Hôpital Avicenne, AP-HP: Christelle Hilaire-Schneider, Frédéric Adnet, Sheila Gasmi / Ségolène Brichler.
Hôpital Jean Verdier, AP-HP: Stéphanie Le Sachey, Romain Dufau
Hôpital Pitié-Salpêtrière, AP-HP: Pierre Hausfater, Marta Cancella de Abreu / Basma Abdi.
Hôpital Tenon, AP-HP: Hélène Goulet, Hélène Buzelin-Cesto / Corinne Amiel.
Hôpital Beaujon, AP-HP: Boris Couve, Matthieu Gay, Prabakar Vaittinada Ayar / Frédéric Bert.
Hôpital Paris St Joseph: Anne Boureau, Olivier Ganansia / Alban Le-Monnier.
Hôpital Bichat, AP-HP: Carine Gauffriaud, Antoine Guillet, Christophe Choquet / Benoit Visseaux.
Hôpital Européen Georges Pompidou, AP-HP: Anne-Laure Féral, Alicia Maunoir / David Veyer.
Hôpital Louis Mourier, AP-HP: Nicolas Javaud, Francis Gace, Carten Correia / Luce Landraud.
Hôpital Saint Antoine, AP-HP: Youri Yordanov / Aurélie Schnuriger.
