# Supplementary material for: Intensified screening for SARS-CoV-2 in 18 emergency departments in the Paris metropolitan area, France (DEPIST-COVID): A cluster-randomized, two-period, crossover trial
Source: PLoS Med. 2023 Dec 7;20(12):e1004317. doi: 10.1371/journal.pmed.1004317 (PMC10735176; doi:10.1371/journal.pmed.1004317)
Supplement: Attachment — Submitted filename: Response_Editor_reviewers_R1_20230906.docx [file pmed.1004317.s002.docx]

**Manuscript "Intensified screening for SARS-CoV-2 in 18 emergency departments: the cluster-randomized, two-period, crossover DEPIST-COVID trial"**

**(PMEDICINE-D-23-01467R1)**

**Answer to Editor and Reviewer comments and requests**

# EDITOR COMMENTS

GENERAL COMMENTS

Author’s answer

We thank you for the global assessment and encouraging remarks.

The comments have been addressed as detailed below.

1. Please cite the reference numbers in square brackets (e.g., “We used the techniques developed by our colleagues [19] to analyze the data”). Citations should be preceding punctuation.

Author’s answer

Reference numbers in the manuscript and eSupplement have been carefully checked to comply with the recommendation. Only S1 Appendix "Study protocol as submitted to the French authorities" has not been modified to keep an identical version to the one submitted.

2. Please cite your Supporting Information as outlined here: <https://journals.plos.org/plosmedicine/s/supporting-information>

Author’s answer

The text has been revised to ensure adherence to the PLOS Medicine guidelines.

3. Causal language - In trials, there is usually a distinction in the language in terms of causal vs associational for primary and secondary trial outcomes. It would be beneficial to use associational language in the discussion and other sections for secondary outcomes.

Author’s answer

According to your suggestion, the abstract and the discussion sections were revised as follows:

*“The ED population seems to be more affected than the population of the region”*

(revised manuscript, page 3, line 60)

*“The proportion of new diagnoses among this asymptomatic/paucisymptomatic population in EDs seems to be higher than that in the asymptomatic population screened during the same period in the region.* *Positivity appeared higher in EDs in individuals born in countries other than France, corroborating studies reporting higher infection rates in minority groups (…)”*

(revised manuscript, page 18, lines 437-441)

*In conclusion, intensified ED screening was unlikely to bring a substantial benefit to the detection of SARS-CoV-2 infections. This strategy could facilitate access to a* *population that appears to be more affected than the population of the region and could help to estimate the incidence of asymptomatic/paucisymptomatic infections.*

(revised manuscript, page 20, lines 510-513)

# ACADEMIC EDITOR COMMENTS

Author’s comment

We greatly appreciate your highly relevant remarks that enabled us to significantly improve the text.

All your comments have been addressed as detailed below.

1. I agree with one of the reviewers who said that there should be more information on the screening of the community in Paris. How was this data collected and how representative is the screened population?

Author’s answer

Following your comment and the comment of Reviewer #3, additional information regarding the screening data in the Paris metropolitan region has been added to clarify the text. These data are not research data and are collected routinely by Santé Publique France, a French organization responsible for surveillance of SARS-CoV-2 screening data. They include all SARS-CoV-2 screening data for individuals tested in the community and in healthcare settings in the region. The data by day and by geographical department are accessible online [23].

Symptomatic status is routinely collected but this part of the data is not made public. At the request of the research team, screening data in the region including symptomatic status were provided by Santé Publique France on May 20, 2022.

In the text, the screening data in the region are referred to as "community screening” data for ease of reading. The Methods section has been revised, as follows:

*“Measurements*

*(...) Community screening data routinely collected for all individuals screened in the Paris metropolitan region [23], including symptomatic status, were provided by Santé Publique France.*

*(...) The secondary outcomes were as follows: (...) (c) Proportions of* *new SARS-CoV-2 infections diagnosed through ED screening for asymptomatic/paucisymptomatic patients versus* *proportions of positive tests through community screening for asymptomatic adults in the geographical departments of the Paris metropolitan region during the same period, overall and per ED; proportion of new SARS-CoV-2 infections diagnosed* *through ED screening for all patients versus proportion of positive tests* *through community screening for all adults in the geographical departments of the Paris metropolitan region during the same period”*

(revised manuscript, page 9, lines 212-213 and page 10, lines 226-234)

*“Statistical analysis (...) The proportions of new SARS-CoV-2 infections diagnosed* *through ED screening for asymptomatic/paucisymptomatic patients overall, per ED and the same proportion through ED screening for all patients were compared to the proportions of positive tests through community screening for adults screened in the* *geographical departments of the Paris metropolitan region using the* *Miettinen-Nurminen (MN) method [25-27] for risk difference and* *95% confidence interval (CI) estimations.*

(revised manuscript, page 11, lines 282-287)

The findings section was also revised:

*“At a regional level, the proportion of new diagnoses through ED screening for asymptomatic/paucisymptomatic patients was higher than the proportion of positive tests through community screening for asymptomatic adults in the departments of the Paris metropolitan region (224/4,283 (5.2%) and 156,298/3,400,584 (4.6%), respectively, risk difference: 0.6% (95% CI [0.01%, 1.3%]; p=0.048)) (S10 Appendix).*

*The proportion of new diagnoses through ED screening for all patients was higher than the proportion of positive tests through community screening for adults in the departments of the Paris metropolitan region (3,943/40,367 (9.8%) and 361,089/4,680,491 (7.7%), respectively, risk difference: 2.1% (95% CI [1.8%, 2.3%]; p<0.001)) (S10 Appendix).”*

(revised manuscript, page 16, lines 392-400)

The abstract was revised as follows:

*“Secondary outcomes included: proportion of new diagnoses through ED screening for asymptomatic/paucisymptomatic patients versus proportion of positive tests through community screening for asymptomatic adults in the* *Paris metropolitan region (…).*

*(…) The proportion of new diagnoses through* *ED screening for asymptomatic/paucisymptomatic patients was higher than that through community screening in the Paris metropolitan region (224/4,283 (5.2%) and 156,298/3,400,584 (4.6%), respectively; risk difference: 0.6% (95% CI [0.01%, 1.3%]; p=0.048).”*

(revised manuscript, page 2, lines 40-42 and page 3, lines 52-54)

Appendix S10 presents the data analysed for these secondary objectives and has also been revised accordingly.

2. It was at some points unclear whether authors were referring to the data from community screening or to results from modelling. Maybe clarify upfront that these two sources are used for comparison with the general population and clarify throughout the manuscript which of two you are referring to.

Author’s answer

We compared the results of ED screening with those of community screening in the Paris metropolitan region. Please refer to the answer to previous comment #1.

We also assessed the results of ED screening against those of an age-stratified transmission model.

As recommended, the Methods section was revised as follows:

*“Outcomes (…) (d) Proportions of new SARS-CoV-2 infections diagnosed through ED screening versus incidence rate in the Paris metropolitan region estimated from an age-stratified disease model for SARS-CoV-2 transmission.”*

(revised manuscript, page 10, lines 232-234)

The results section was also clarified:

*“The estimates from ED screening were compatible with the predictions of the mathematical model, yielding a regional incidence rate of asymptomatic/paucisymptomatic infections in adults of 4.1% (95% probability ranges [2.5%, 5.7%]) and a regional incidence rate in adults (symptomatic and asymptomatic) of 9.7% (95% probability ranges [6.1%, 13.8%]).”*

(revised manuscript, page x, lines x-x).

3. In the tables, the term "control strategy" for denoting data collected during control periods could potentially be misleading. In my understanding, the term "control strategy" refers to a strategy to control the pandemic, and not control in the sense of control group. I suggest to write "control periods" and "intervention periods" or something similar.

Author’s answer

According to your suggestion, the terms “control strategy” and “intervention strategy” were modified in the tables 1 and 2 to: “control period” and “intervention period” (revised manuscript, pages 13 and 15). This change has also been reported in the Table of S8 Appendix.

4. COMPETING INTEREST STATEMENT

All authors must declare their relevant competing interests per the PLOS policy, which can be seen here: <https://journals.plos.org/plosmedicine/s/competing-interests>

For authors with ties to industry, please indicate whether any of the interests has a financial stake in the results of the current study.

Author’s answer expected

Thank you for this reminder. All relevant competing interests have been declared in the declaration of interests.

5. FINANCIAL DISCLOSURE

The funding statement should include: specific grant numbers, initials of authors who received each award, URLs to sponsors’ websites. Also, please state whether any sponsors or funders (other than the named authors) played any role in study design, data collection and analysis, the decision to publish, or preparation of the manuscript. If they had no role in the research, include this sentence: “The funders had no role in study design, data collection and analysis, decision to publish, or preparation of the manuscript.”

Author’s answer expected

The funding statement has been corrected to meet PLOS policy expectations, as follows:

*“The study was funded by the Agence Nationale de Recherche sur le Sida et les Hépatites Virales | Maladies Infectieuses Emergentes (ANRS|MIE) (https://anrs.fr/fr/), Paris (JL) and by the Région Île-de-France (https://www.iledefrance.fr/), France (JL).*

*The sponsor was Assistance Publique-Hôpitaux de Paris (Département de la Recherche Clinique et du Développement).*

*The funders of the study had no role in study design, data collection and analysis, decision to publish or preparation of the manuscript.”* (revised title page)

6. DATA AVAILABILITY STATEMENT

PLOS Medicine requires that the de-identified data underlying the specific results in a published article be made available, without restrictions on access, in a public repository or as Supporting Information at the time of article publication, provided it is legal and ethical to do so. Please see the policy at <http://journals.plos.org/plosmedicine/s/data-availability> and FAQs at <http://journals.plos.org/plosmedicine/s/data-availability#loc-faqs-for-data-policy>

The Data Availability Statement (DAS) requires revision. For each data source used in your study:

a) If the data are freely or publicly available, note this and state the location of the data: within the paper, in Supporting Information files, or in a public repository (include the DOI or accession number).

b) If the data are owned by a third party but freely available upon request, please note this and state the owner of the data set and contact information for data requests (web or email address). Note that a study author cannot be the contact person for the data.

c) If the data are not freely available, please describe briefly the ethical, legal, or contractual restriction that prevents you from sharing it. Please also include an appropriate contact (web or email address) for inquiries (again, this cannot be a study author).

In accordance with ICMJE requirements, PLOS Medicine requires prospective, public registration of a data sharing plan (as part of mandatory clinical trials registration) for all clinical trials that began enrollment on or after January 1, 2019.

Author’s answer

The data are not freely available (option c), and this has been clarified in the Data Availability Statement. Public registration of the data sharing plan will be updated in Clinicaltrials accordingly. The Title page has been revised as follows:

*“**The study data are owned by the Assistance Publique - Hôpitaux de Paris (AP-HP) sponsor, “Département de la Recherche Clinique et du Développement”. The data are not freely available.*

*No data may be transmitted unless a contract has first been signed between the AP-HP and an external partner interested in the data.* *For all inquiries, please contact the following:*

*DJENNAOUI Fatiha <*[*fatiha.djennaoui@aphp.fr*](mailto:fatiha.djennaoui@aphp.fr)*> and [DRC] Secretariat Promotion Délégation à la Recherche Clinique et à l'Innovation <*[*drc-secretariat-promotion@aphp.fr*](mailto:drc-secretariat-promotion@aphp.fr)*>.*”

### ABSTRACT

7. Please report your abstract according to CONSORT for abstracts, following the PLOS Medicine abstract structure (Background, Methods and Findings, Conclusions); <https://www.equator-network.org/reporting-guidelines/consort-abstracts/>

Please ensure that all numbers presented in the abstract are present and identical to numbers presented in the main manuscript text.

Author’s answer

The structure of the abstract has been revised to correspond to the recommendations of PLOS Medicine and CONSORT for abstracts (revised manuscript, pages 2-3). All numbers presented in the abstract are identical to numbers presented in the main manuscript text.

8. PLOS Medicine requests that main results are quantified with 95% CIs as well as p values. Please include. When reporting p values please report as p<0.001 and where higher as the exact p value p=0.002, for example. For the purposes of transparent data reporting, if not including the aforementioned please clearly state the reasons why not.

Author’s answer

Following your suggestion, p-values were added for main results in the Abstract and Results sections, in addition to 95% CIs as follows:

*“(adjusted analysis:* *26.7/1,000 versus 26.2/1,000, adjusted relative risk: 1.02 (95% confidence interval (CI) [0.94, 1.11]; p=0.634)).* *(…)*

*(224/4,283 (5.2%) and 156,298/3,400,584 (4.6%), respectively; risk difference: 0.6% (95% CI [0.01%, 1.3%];* *p=0.048). (…)*

*(3,943/40,367 (9.8%) and 361,089/4,680,491 (7.7%), respectively, risk difference: 2.1% (95% CI [1.8%, 2.3%];* *p<0.001))”*

(revised manuscript, page 3, lines 50-54, page 15, lines 376-378 and page 16, lines 392-400).

The Methods section was also revised as follows:

*“The P values reported for covariates were based on t tests using the Kenward-Roger approximation for the denominator degrees of freedom. (…)*

*Pearson X^2^ test was used to test for differences in these secondary outcomes.”*

(revised manuscript, page 10, lines 254-255 and page 11, line 287).

9. Please include any important dependent variables that are adjusted for in the analyses.

Author’s answer

Following the recommendation, the Methods and findings section in the abstract was clarified:

*“**The* *primary outcome was analyzed using a generalized linear mixed model (Poisson distribution) with the center and center by period as random effects, the strategy (intervention versus control) and period (modeled as a weekly categorical variable)* *as fixed effects with additional adjustment for community incidence.”*

(revised manuscript, page 2, lines 37-40).

10. Throughout, suggest reporting statistical information as follows to improve clarity for the reader “22% (95% CI [13%,28%]; p</=)”. Please amend throughout the abstract and main manuscript.

Author’s answer

As recommended, throughout the manuscript, the main endpoints have been expressed with a 95% CI and a p-value. Please refer to the answer to previous comment #8.

11. Please note the use of commas to separate upper and lower bounds, as opposed to hyphens as these can be confused with reporting of negative values.

Author’s answer

Thank you for your vigilance. The abstract has been corrected. The remaining text in the manuscript and the eSupplements have also been proofread to check that it complies with this suggestion.

12. When a p value is given, please specify the statistical test used to determine it. Please report p values as p<0.001 and where higher as 'p=0.002'

Author’s answer

Please refer to the answer to previous comment #8.

13. Please state that analysis was intention to treat.

Author’s answer

The following text was added to the revised version of the abstract:

*“Primary analysis was intent-to-treat.”*

(revised manuscript, page 2, line 37).

14. Please provide the number of participants lost to follow up in each group.

Author’s answer

We understand your concern. For the primary outcome of this study, patients who tested positive and whose SARS-CoV-2-positive status was not already known were considered positive.

All other patients who may be unscreened, whose test result was negative, indeterminate, or positive with a known status <3 months were classified as nonpositive.

The study therefore did not include dropouts and data were not imputed. The study was analysed on an intention-to-treat basis.

These elements can be found in the Methods section.

15. Abstract Background: Provide the context of why the study is important. The final sentence should clearly state the study question.

Author’s answer

Following this suggestion, the text of the abstract was revised:

“*Background: Asymptomatic and paucisymptomatic infections account for a substantial portion of SARS-CoV-2 transmissions. The value of intensified screening strategies, especially in emergency departments (EDs),* *in reaching asymptomatic and paucisymptomatic patients and helping to improve detection and reduce transmission has not been documented. The objective of the study was to evaluate in 18 EDs whether an intensified SARS-CoV-2 screening strategy combining nurse-driven screening for asymptomatic/paucisymptomatic patients with routine practice (intervention)* *could contribute to higher detection of SARS-CoV-2 infections compared to routine practice alone including screening for symptomatic or hospitalized patients (control)*.”

(revised manuscript, page 2, lines 21-28).

16. Abstract Methods and Findings:

*Please provide brief demographic details of the study population (e.g. sex, age). Please define the time frame during which the study took place.

* Please include a summary of adverse events if these were assessed in the study.

* In the last sentence of the Abstract Methods and Findings section, please describe the main limitation(s) of the study's methodology.

Author’s answer

In response to your suggestions, the information requested has been added to the abstract:

- The demographic details of the population were added to the abstract, as follows:

"*During the intervention and control periods, 69,248 patients and 69,104 patients, respectively, were included for a total of 138,352 patients. They had a median age of 45.0 years [31.0; 63.0], and women represented 45.7% of the patients.*

(revised manuscript, page 2, lines 44-46).

This change was also reported in the Results section of the revised manuscript (page 13, lines 315-317).

- The time frame during which the study took place was added as follows:

*“During a cluster-randomized, two-period, crossover trial from February 2021 to June 2021 (…)”*

(revised manuscript, page 2, line 30).

- The fluctuating evolution of the epidemic over the course of the trial, which is a limitation of the study, was mentioned in the abstract:

*“**This finding was obtained in a rapidly evolving epidemiological context.”*

(revised manuscript, page 3, line 59).

- Reporting of adverse events was not requested in the study.

17. Abstract Conclusions:

Please begin your Abstract Conclusions with "In this study, we observed ..." or similar, to summarize the main findings from your study, without overstating your conclusions. Please emphasize what is new and address the implications of your study, being careful to avoid assertions of primacy.

Author’s answer

The Conclusion section of the abstract was revised; the following sentence was added:

*“Conclusion**s*

*In this study, intensified screening* *for SARS-CoV-2 in EDs was unlikely to identify a higher proportion of newly diagnosed patients**. This finding was obtained in a rapidly evolving epidemiological context.* *The ED population seems to be more affected than the population of the region, and the results highlight the potential of the intensified screening strategy for the estimation of regional incidence for asymptomatic/paucisymptomatic infections.”*

(revised manuscript, page 3, lines 58-62).

18. l.40: Please define ‘aRR’.

l.41: Please define ‘CI’.

Author’s answer

We have revised the text as recommended.

19. AUTHOR SUMMARY

At this stage, we ask that you include a short, non-technical Author Summary of your research to make findings accessible to a wide audience that includes both scientists and non-scientists. The Author Summary should immediately follow the Abstract in your revised manuscript. This text is subject to editorial change and should be distinct from the scientific abstract. Please see our author guidelines for more information: <https://journals.plos.org/plosmedicine/s/revising-your-manuscript#loc-author-summary>.

The summary should include 2-3 single sentence, individual bullet points under each of the questions. The last bullet point should describe the main limitation(s) of the study's methodology.

It may be helpful to review currently published articles for examples which can be found on our website here <https://journals.plos.org/plosmedicine/>

Author’s answer

Following your recommendation, an author summary was provided in the revised manuscript. Please find the text below:

***« AUTHOR SUMMARY***

- ***Why Was This Study Done?***
  - *Undetected asymptomatic SARS-CoV-2 infections or paucisymptomatic* *infections with mild symptoms are responsible for a substantial portion of transmissions, which has been a major challenge for managing the COVID-19 pandemic. Free and widely available screening has been offered in many countries to reach asymptomatic/paucisymptomatic infectious individuals and improve detection, reduce transmission and help contain the pandemic.*
  - *Systematically offering screening during medical consultations, particularly in emergency departments (EDs), is a possible strategy to identify asymptomatic/paucisymptomatic infectious* *individuals.*
  - *The value of intensified screening strategies in EDs has not been evaluated.*
- ***What Did the Researchers Do and Find?***
  - *The trial was designed to assess the value of an* *enhanced SARS-CoV-2 screening strategy* *which included screening for asymptomatic / paucisymptomatic patients* *in addition to routine screening* *of symptomatic and hospitalized patients in EDs, compared with routine screening practice alone.*
  - *The study was conducted in 2021 prior to vaccine roll-out in 18 EDs in the Paris metropolitan area and involved 138,352 patients. During the intervention period, 4,283 asymptomatic / paucisymptomatic patients were screened, leading to 224 new diagnoses. Overall, 1,859 patients were newly diagnosed during the intensified screening period versus 2 084 patients during the routine practice period, corresponding, after analysis, to 26.7 new diagnoses / 1,000 patients during the intensified screening period versus 26.2 new diagnoses / 1,000 patients during the routine practice period.*
  - *The population screened in EDs appears to be more affected than the general population screened during the same period in the region. Comparison with findings from a mathematical model indicates that the ED data allow estimation of regional incidences for asymptomatic/paucisymptomatic infections.*
- ***What Do These Findings Mean?***
  - *The intensified screening strategy was unlikely to bring a substantial benefit* *to the detection of SARS-CoV-2 infections. These findings were obtained in a changing epidemiological context.*
  - *Intensified screening in EDs could allow access to individuals who are less likely to be reached by the general screening system.*
  - *Our work encourages further exploration of screening strategies for asymptomatically transmitted respiratory viruses to better define the observatory role of EDs in emerging epidemics.”*

(revised manuscript, page 4, lines 64-98 and page 5, lines 99-101).

### INTRODUCTION

20. Please address past research and explain the need for and potential importance of your study. Indicate whether your study is novel and how you determined that. If there has been a systematic review of the evidence related to your study (or you have conducted one), please refer to and reference that review and indicate whether it supports the need for your study.

Author’s answer

Thank you for your comment. The introduction has been revised to better highlight studies conducted on SARS-CoV-2screening strategies in the general population to limit asymptomatic transmission. The lack of documentation of these screening procedures and the mainly observational design of the studies have been highlighted. The importance and innovative nature of our study conducted among a large population consulting in emergency departments and being one of the rare pragmatic randomized controlled trials on screening have thus been underlined.

We conducted a search in MEDLINE to retrieve studies that assessed the effectiveness of screening for asymptomatic/paucisymptomatic adults in EDs, apart from inpatient screening. On January 15, 2023, we searched MEDLINE for publications without date restrictions using the search terms “COVID-19” AND “Emergency service, hospital” AND (“Screening” or “Testing”) and their synonyms, including both MeSH terms and title/abstract searches.

The search yielded 762 results, which included 57 publications that were substantially relevant to the topic, the majority of which focused on testing symptomatic patients for diagnosis purposes or testing admitted patients to reduce nosocomial transmission (n=44), reported the accuracy of screening tests (n=6) or were modeling studies. We concluded that the value of intensified screening in EDs to reach asymptomatic/paucisymptomatic individuals to better control the epidemic has not been evaluated.

The search was updated to date and did not retrieve any additional relevant studies. This literature review was not mentioned as such in the manuscript since it has not been published. However, the results are described in the discussion section.

Please find below the revised version of the introduction section:

*“Countries have experienced several waves of the SARS-CoV-2 pandemic, with considerable health and economic impacts [1]. Containing the pandemic was challenged by undetected asymptomatic or paucisymptomatic infections, which are estimated to account for nearly half of all transmissions [2-4].*

*In many countries, along with* *nonpharmaceutical control measures, widely available and free screening for SARS-CoV-2 has been offered in communities to reach infected asymptomatic/paucisymptomatic persons who would otherwise not have been tested. Mass* *screening experiments have been conducted at the city or country level [5-7]. In Slovakia, a population-wide screening program in 2020 led to a reduction in the prevalence of infection [6]. In 2020-2021, community-based asymptomatic screening in Liverpool was associated with a reduction in COVID-19-related hospital admissions compared with a control population in England [7]. Difficulties in reaching the most deprived populations have been noted in these screening programs [5, 8], as well as technical constraints due to the high volume of tests [6] and/or the poor sensitivity of some antigenic tests.* *Overall, research evaluating the impact of SARS-CoV-2 screening programs on improving detection, reducing transmission, and contributing to containing the pandemic is scarce. A recent systematic review also points out that the studies were modeling studies or retrospective observational analyses not conducted in a controlled environment [9].*

*A possible wide screening strategy for identifying asymptomatic/paucisymptomatic persons is to systematically offer screening during medical consultations, particularly in emergency departments (EDs).* *French EDs receive each year a volume equivalent to one-third of the French population [10,11] and provide access to a large portion of the population, including low-income groups who may be less reached by the general screening system. Therefore, intensified SARS-CoV-2 screening in EDs could contribute to epidemic control and provide an opportunity to observe viral evolution.*

*In addition to SARS-CoV-2 screening in EDs for symptomatic patients, screening at the time of hospital admission has been mandatory since mid-2020 in European countries to limit nosocomial transmission after studies showed that a high number of inpatients, up to 50% of new positive cases, were asymptomatic carriers [12, 13]. Evaluations of screening practices in EDs for inpatients highlighted the value of the strategy during periods of high prevalence [13-17]. However,* *to our knowledge, no study has evaluated the benefit of extending screening to all patients visiting the EDs in reaching asymptomatic and paucisymptomatic infectious individuals and helping improve detection. The lessons learned from the implementation and evaluation of such measures could be useful in preparing for future epidemics.*

*The objectives of this trial were to offer an intensified screening strategy in 18 EDs in the Paris metropolitan region before the vaccine roll-out to evaluate* *whether* *this strategy* *could contribute to a higher detection proportion of SARS-CoV-2 infections during a period covering the third wave caused by the SARS-CoV-2 alpha variant.”*

(revised manuscript, pages 6-7, lines 104-139).

In the discussion section, the following paragraph related to the literature search was added:

*“Data from the implementation of the intensified SARS-CoV-2 screening strategy in EDs are difficult to compare. Most studies evaluating the effectiveness of SARS-CoV-2 screening in adult EDs either focus on screening symptomatic patients for diagnostic purposes or screening admitted patients to reduce nosocomial transmission, are modeling studies, or report on the accuracy of screening tests.”*

(revised manuscript, page 18, lines 450-453).

21. l.65: Please revise tense. We suggest ‘The objectives of this study were to…’ or similar.

Author’s answer

The text was revised accordingly:

“*The objectives of this trial were to offer an intensified screening strategy in 18 EDs in the Paris metropolitan region* *before the vaccine roll-out to evaluate whether this strategy could contribute to a higher detection proportion of SARS-CoV-2 infections (…)”*

(revised manuscript, page 7, lines 136-138).

### METHODS AND RESULTS

22. Thank you for providing the completed CONSORT extension to cluster randomised trials checklist. Please ensure that all components of CONSORT are present in the manuscript, including how randomization was performed, allocation concealment, blinding of intervention, definition of lost to follow-up, power statement. Please include the completed CONSORT checklist as Supporting Information. When completing the checklist, please use section and paragraph numbers, rather than page numbers.

Author’s answer

Following your suggestion, the CONSORT extension to cluster randomised trials checklist has been revised to verify that all components of CONSORT are present in the manuscript. The revised checklist includes section and paragraph numbers and is included in the Supporting Information (Appendix 12). Please refer to the answer to previous comment #14 for data on the lost to follow-up.

23. Please add the following statement, or similar, to the Methods: "This study is reported as per the Consolidated Standards of Reporting Trials (CONSORT) guideline for cluster randomised trials (S1 Checklist)."

Author’s answer

The following sentence has been added to the Methods section to account for your comment:

“*This study is reported as per the cluster randomized trials extension of Consolidated Standards of Reporting Trials (CONSORT) guideline (S12 Checklist) [31].*“

(revised manuscript, page 12, lines 310-311).

24. If the study included dropouts, specify whether their data are imputed and if so using what method, please refer to as modified ITT.

Author’s answer

Please refer to the answer to previous comment #14.

25. PLOS Medicine requests that main results are quantified with 95% CIs as well as p values. When reporting p values please report as p<0.001 and where higher as the exact p value p=0.002, for example. For the purposes of transparent data reporting, if not including the aforementioned please clearly state the reasons why not.

Author’s answer

Please refer to the answer to previous comments #8 and #10.

26. Please include any important dependent variables that are adjusted for in the analyses.

Author’s answer

All the variables that are adjusted for in the analyses are described in the Methods section and we then refer to the adjusted analysis in the results section.

27. Suggest reporting statistical information as detailed above – see under ABSTRACT

Author’s answer

Please refer to the answer to previous comments #8 and #10.

28. Please present numerators and denominators for percentages, at least in the Tables [not necessarily each time they're mentioned].

Author’s answer

Following your suggestion, numerators and denominators for percentages were added in the text and in Table 1:

*“Overall, 4,283 asymptomatic/paucisymptomatic patients were screened, including 3,682 (86,0%) patients who underwent a rapid molecular SARS-CoV-2 test, resulting in 224 (5.2%) new SARS-CoV-2 diagnoses.* *(…) Among the 601 patients who underwent a rapid multiplex respiratory virus test, 116 (19.3%) were newly diagnosed with SARS-CoV-2, including 5 diagnosed with other respiratory viruses, and 72 (12.0%) who tested negative for SARS-CoV-2 were positive for another virus.*“

(revised manuscript, page 14, lines 346-353).

*“The factors associated with performing screening for asymptomatic/paucisymptomatic patients were male sex (54.6% of patients screened versus 51.4% of patients who declined screening, OR: 1.21 (95% CI [1.05, 1.40**])), mild symptoms (19.1% versus 6.4%, OR: 2.8 (95% CI [2.1, 3.7])), not being screened previously (41.6% versus 31.7%, OR: 1.4 (95% CI [1.2, 1.6])) and self-assessment of high risk of infection (17.8% versus 14.2%, OR: 1.3 (95% CI [1.1, 1.6**])) (**S4 Appendix).*

*The factors associated with new diagnoses through screening for asymptomatic/paucisymptomatic patients were older age (OR: 1.02 (95% CI [1.01, 1.03]) per additional year), not being from France (54.4% of patients with new diagnoses versus 40% of patients tested negative, OR: 1.7 (95% CI [1.2, 2.5])), particularly being from Sub-Saharan Africa, having mild symptoms (61.6% versus 16.5%, OR: 7.3 (95% CI [5.1, 10.4])), being case-contact (29.7% versus 22.0%, OR: 1.8 (95% CI [1.2, 2.6])) and not being screened previously (57.7% versus 41.4%, OR: 2.1 (95% CI [1.5, 3.0])) (S5 Appendix).*

*At a regional level, the proportion of new diagnoses through ED screening for asymptomatic/paucisymptomatic patients was higher than the proportion of positive tests through community screening for asymptomatic adults in the departments of the Paris metropolitan region (224/4,283 (5.2%) and 156,298/3,400,584 (4.6%), respectively, risk difference: 0.6% (95% CI [0.01%, 1.3%]; p=0.048)) (S10 Appendix).*

*The proportion of new diagnoses through ED screening for all patients was higher than the proportion of positive tests through community screening for adults in the departments of the Paris metropolitan region (3,943/40,367 (9.8%) and 361,089/4,680,491 (7.7%), respectively, risk difference: 2.1% (95% CI [1.8%, 2.3%];* *p<0.001) (S10 Appendix).”*

(revised manuscript, page 15, lines 381-388 and page 16, lines 389-400)

29. Where questionnaires were used, please provide a copy of the questionnaire in the supplementary files.

Author’s answer

The DEPIST-COVID questionnaire can be found at the end of S1 Appendix.

30. Please define "lost to follow-up" as used in this study. Other reasons for exclusion should be defined.

Please define the length of follow up (eg, in mean, SD, and range).

Author’s answer

Thank you for the comment. There were no drop-outs for the primary outcome. Please refer to the answer to previous comment #14.

When the protocol was drawn up, it was planned that patients would receive a call within 15 ± 10 days of their visit to the ED to assess their discharge status (see appendix S1), i.e., whether they were admitted to the hospital or discharged from the ED after the screening test had been carried out. For feasibility reasons, this call was not possible.

Discharge status data were collected retrospectively from patient care records in 8 centres for the 15-day period. In these 8 centres, of the 2,226 patients offered nurse-driven SARS-CoV-2 screening for asymptomatic or paucisymptomatic patients, 1,793 had ED discharge data (results presented in S4 appendix). It was not possible to collect these data in the remaining 10 centres.

In this table and in the Results section, the term “follow-up” is not appropriate and has been replaced by “ED discharge data” for greater clarity, as follows:

“*Of the 1,793 patients offered screening for whom ED discharge data were available, 1,769 (98.7%) were not hospitalized (outpatients) (S4 and S11 Appendixes).“*

(revised manuscript, page 14, lines 333-335).

31. Please provide the actual numbers of events for the outcomes, not just summary statistics or ORs.

Author’s answer

Please refer to the answer to previous comment #28.

32. Please specify whether informed consent was written or oral.

Author’s answer

The information and consent procedure included the following steps:

- Information about the study was provided via posters in the ED during each study period, intervention and control period.
- An individual paper information note was given to patients meeting the inclusion criteria (> 18 years) on arrival, during each study period.
- Nurses verbally asked patients for "explicit" consent (called “express consent” in the French law), please refer to the study protocol on page 22 in S1 Appendix.

The text has been revised to describe these stages more precisely:

*“Information about the study was provided to patients through posters during each study period in the EDs. An individual information note was given to patients meeting the inclusion criteria (> 18 years) on arrival to obtain their express informed consent to participate in the study and data collection during each study period, in accordance with French law (S1 Appendix). During the intervention period, in addition to routine screening practice, all asymptomatic/paucisymptomatic patients were offered a questionnaire and a screening test.”*

(revised manuscript, page 7, lines 156-161).

33. The following secondary outcomes measures

“For symptomatic patients: description of the symptomatology”,

“Number of close contact persons who have been tested for SARS-Cov2, who have been tested positive and who have been isolated over both periods.”,

“Sociodemographic, behavioural factors and habits associated with SARS-CoV-2 infection in patients tested in the following three schemes: DEPIST-COVID (positive cases, negative controls), ComCor (cases, controls) and COVISAN”

and

“Estimate of incidence in the region, estimate of underdetection of cases in the region, and comparison with results from the model experiment”

appear to differ between the submitted manuscript and the trial registry/study protocol. Please clarify and explain the discrepancy.

(a) Can you please present those results as part of this manuscript, or indicate why that is not possible?

(b) Can you please indicate when you plan to publish those results?

Author’s answer

We understand your concerns and have provided details for each objective below.

- The following objective “For symptomatic patients: description of the symptomatology” has been described in the Methods section as included in the description of the characteristics of the patients who completed the questionnaire:

*“The secondary outcomes were as follows: (a)* *Implementation of ED screening for asymptomatic/paucisymptomatic patients, including the proportion of patients who completed the questionnaire; the proportion of screening tests that were offered, accepted, and performed; and patient characteristics reported in the DEPIST-COVID questionnaire”*

(revised manuscript, page 9, lines 220-223).

Data relating to the symptomatology of patients who completed the DEPIST-COVID questionnaire are described in S5 Appendix.

- The objective “Estimate of incidence in the region, estimate of underdetection of cases in the region, and comparison with results from the model experiment” has been described in the Methods section and in the Findings section (revised manuscript, page 12, lines 292-307 and page 16, lines 401-404).

We did not compute the underdetection of cases in the region from the results of the mathematical model, as we directly compared the model-predicted incidence in the region (not affected by underdetection biases) with the corresponding estimate resulting from the screening in EDs (revised manuscript, page 16, lines 401-404). The two estimates were compatible, indicating that ED data allowed for the estimation of case incidence. It was therefore no longer necessary to estimate underdetection.

- For the following objective: “Number of close contact persons who have been tested for SARS-CoV-2, who have been tested positive and who have been isolated over both periods”, the data have not been collected for feasibility reasons. This objective cannot be analysed.
- For the objective “Sociodemographic, behavioural factors and habits associated with SARS-CoV-2 infection in patients tested in the following three schemes: DEPIST-COVID (positive cases, negative controls), ComCor (cases, controls) and COVISAN”, data were also not collected to meet this objective, which required each ED patient diagnosed as positive for SARS-CoV-2 to complete a ComCor questionnaire, which was not possible for feasibility reasons. Patients taking part in another home screening programme, called the COVISAN programme, were also asked to complete the ComCor questionnaire, but this was not done either.

Overall, in a complex research context after 1 year of the COVID-19 pandemic, the objectives of the DEPIST-COVID study could not all be achieved, which explains the discrepancy between the announced objectives and those presented. There is no plan to publish other results from this study.

34. Please include the study protocol document and analysis plan, with any amendments, as Supporting Information to be published with the manuscript if accepted.

Author’s answer

The Supporting Information includes the study protocol document and the analysis plan with amendments; please refer to S1 Appendix.

The initial version of the "DEPIST-COVID" protocol, version 1.1 dated January 08, 2021, was approved by the French authorities and was followed by version 2.0 dated April 06, 2021, which was also approved. The study protocol document provided in S1 appendix is version 2.0. The amendment in this version 2.0 concerned the following 3 elements:

a) Extension of the study duration by 30 days:

Research schedule, page 8 of S1 Appendix:

“2 months (with the possibility of extending each period by a maximum of 15 days maximum), i.e., 2 months and 30 days maximum + wash-out.”

b) Modification of the ancillary study, which corresponds to a separate protocol.
c) Change in the principal investigator for one center.

To improve clarity, this information has been added at the beginning of S1 Appendix.

35. l.71: Please define ‘ANRS’.

Author’s answer

The following sentence was revised:

“*The protocol of the ANRS|MIE DEPIST-COVID trial is available in S1 Appendix (ANRS-MIE refers to “Agence nationale de recherche sur le sida et les hépatites virales* *| Maladies Infectieuses Emergentes”).”*

(revised manuscript, page 7, lines 146-148).

36. l.94: “duration of each period was a minimum of 1 month with the possibility of a 15-day extension”. In the study procotol it was describe that duration of inclusion was intended to be 2 months (with the possibility of extending each period by a maximum of 15 days; 2 months and 30 days maximum + wash-out?. Please clarify and explain the discrepancy.

Author’s answer

Each centre had an intervention period and a control period. The study protocol states that: “*The duration of each period will be one month (with the possibility of extending the period to a maximum of 1 month and 15 days*)”, please refer to page 19 of S1 Appendix.

The study protocol also states that “*The total duration of the study will be 2 months and 30 days maximum (+ wash-out) per center*”, please refer to page 20 of S1 Appendix.

The article states that “*The duration of each period was a minimum of 1 month with the possibility of a 15-day extension*“ (L.94 of the initial version of the manuscript), which is consistent with the study protocol.

37. l.121: Please define ‘PCR’.

Author’s answer

The text was revised accordingly.

38. ll.142-157: Please improve the presentation of the secondary outcomes with special attention to clarity and structure.

Author’s answer

Following your comment, the presentation of the secondary outcomes was revised by clarifying the structure and standardising the terms used. Please refer to the answer to previous comments #1 and #2.

39. l.189: “Implementation measures were described”. I am not sure what this sentence is intended to mean. Please revise this sentence.

Author’s answer

The sentence was revised accordingly:

*“The implementation of ED screening for asymptomatic/paucisymptomatic patients was described.”*

(revised manuscript, page 11, line 266).

40. ll.195-196: “- The proportion of new SARS-CoV-2 and other respiratory virus infections diagnosed through screening for asymptomatic/paucisymptomatic patients and patient characteristics were described.”. Please revise this sentence.

Author’s answer

Following your suggestion, the sentence was revised:

*“The proportions of new SARS-CoV-2 infections and other respiratory virus infections diagnosed through ED screening for asymptomatic/paucisymptomatic patients were described, as well as patient characteristics.”*

(revised manuscript, page 11, lines 272-274).

41. l.213: “- Incidence rate estimation in the Paris metropolitan region” – is this supposed to be a subheader? Please revise.

Author’s answer

The text has been revised as follows, and the subheader has been removed:

“The analysis plan for estimating the incidence rate in the Paris metropolitan region is detailed below.“

(revised manuscript, page 12, line 292).

42. ll.189-213: Please remove the bullet points from your text and revise the paragraphs.

Author’s answer

The bullet points were removed from the text, and the paragraphs were revised.

43. ll. 231-233: Please remove the “Role of the funding source” paragraph. This statement should only be included in the according section in the online submission form.

Author’s answer

The “Role of the funding source” paragraph was removed, following your comment.

### DISCUSSION

44. Please present and organize the Discussion as follows: a short, clear summary of the article’s findings; what the study adds to existing research and where and why the results may differ from previous research; strengths and limitations of the study; implications and next steps for research, clinical practice, and/or public policy; one-paragraph conclusion. Please remove any subheadings.

Author’s answer

The discussion section has been restructured and revised to clearly include all the recommended parts. A comparison with existing research in EDs and with community screening evaluations was added, highlighting the strengths of the study. A section on limitations has been clearly delineated.

Implications for research and clinical practice have also been added in this section.

Please find below the revised parts of the discussion section:

*“In the study, almost half of the asymptomatic/paucisymptomatic patients screened had not been previously tested for SARS-CoV-2, suggesting limited access to health care. The proportion of new diagnoses among this asymptomatic/paucisymptomatic population in EDs seems to be higher than that in the asymptomatic population screened during the same time in the region.* *Positivity appeared higher in EDs in individuals born in countries other than France, corroborating studies reporting higher infection rates in minority groups [32]. These results indicate that EDs may provide access to a large population, including more deprived patients who may be less reached by the general screening system. This is one of the main strengths of the study. Community-based SARS-CoV-2 screening programs struggled to reach a wide range of populations and observed lower participation in most disadvantaged neighborhoods [8]. When epidemic growth was significant, high levels of repeat screening should be maintained [33, 34]. The strategy in EDs can allow repeat screening and specifically include high-risk groups. The findings in community screening and EDs reiterate the importance of examining strategies that overcome the effects of social and health inequalities and may inform the development of future interventions in other epidemics.*

*Data from the implementation of the intensified SARS-CoV-2 screening strategy in EDs are difficult to compare. Most studies evaluating the effectiveness of SARS-CoV-2 screening in adult EDs either focus on screening symptomatic patients for diagnostic purposes or screening admitted patients to reduce nosocomial transmission, are modelling studies, or report on the accuracy of screening tests.*

*Evaluation of community-based SARS-CoV-2 screening programs for asymptomatic individuals involved few controlled comparisons of key health outcomes, such as detection of positive cases, timely isolation of infectious individuals and their close contacts, transmissions, hospital admissions or deaths. Furthermore, the evaluations conducted have often failed to distinguish between the impact of mass screening and a combination of other control measures introduced at the same time. Large-scale community screening evaluated in three countries at rapidly growing phases of the epidemic appeared to have the potential to reduce prevalence, transmission or subsequent hospitalizations, at least in the short term [6, 7, 34]. However, given the heterogeneity of the study designs, objectives, outcomes or the absence of a comparison group, the findings cannot be directly compared with those obtained in EDs. In a study conducted in Switzerland in 2021 with repeat screening of approximately 27,000 individuals over 8 weeks, the authors favorably concluded that the screening program led to a reduction in the incidence rate of up to 50%, with approximately 200 positive tests observed, which is a raw number similar to that obtained with ED screening for asymptomatic / paucisymptomatic individuals [34].*

*Comparative evaluation of screening activities is crucial for identifying effective and equitable responses [7]. The results of the present study raise the issue of the study design choice in a changing epidemiological landscape. The alternative of a cluster randomization and crossover could have been a before-and-after design, but this would have resulted in difficult-to-control biases and a lower level of evidence, therefore, the chosen design is not challenged.*

*There is concern that adding screening will have unintended consequences for ED workload. Contrasting results are reported on the impact of inpatient screening on ED length of stay [15, 22]. Screening yield can also be limited by staff shortages, costs, or testing capacities, encouraging adaptation of screening practices for asymptomatic inpatients and similarly for outpatients to individual-level risk assessment and regional incidence [13, 16].*

*In terms of limitations, in addition to the factors described, including* *the high fluctuation in the epidemic which may have reduced the impact of the intervention, during the study, 14% of the eligible ED patients completed the questionnaire, which was the first stage of the nurse-driven screening strategy for asymptomatic/paucisymptomatic individuals, 67% of whom were screened. This is inherent to screening strategies in EDs, in which the proportion of patients involved in the screening process rarely exceeds 20% [24, 35]. However, considering the high volume of patients visiting EDs, the number of patients reached far exceeds that of other facilities in the same period.*

*A rapid isothermal amplification assay was used for biosafety reasons and rapid turnaround time [22]. Although the performances of such assays might be questionable compared to PCR techniques, several studies evaluating the same test we used reported high specificity (>97.5%) and high sensitivity (>98.5%) provided that tests were performed with dry swabs after nasopharyngeal sampling [36-39]. The test provides qualitative results, and therefore, residual and presymptomatic infections could not be distinguished. The aim was also to choose a test that would be easy and safe for nurses with no prior experience or qualifications in laboratory testing. The test had to provide quick results, similarly to lateral flow assays, possess a higher sensitivity for identifying asymptomatic individuals and offers result traceability. Finally, rapid isothermal assays offer testing options that may be more affordable than single automated real-time PCR assays, particularly in terms of cost of the system.*

*For reasons of feasibility, our study could not include an evaluation of the costs in the two groups compared and an examination of the choice of tests. This is a major area since decisions about the adoption of control measures must reflect both effectiveness and costs [9].*

*Given the significant asymptomatic transmission of SARS-CoV-2 infection, screening is a key nonpharmaceutical measure for identifying infectious individuals, limiting transmission, and containing the epidemic. During a rapidly evolving epidemic phase, we observed in EDs that the intensified screening strategy was unlikely to increase detection. However, comparing ED screening data with a mathematical model was encouraging, as it allowed estimation of regional incidences for both asymptomatic/paucisymptomatic SARS-CoV-2 infections and total cases. This approach can potentially be used to estimate the size of the epidemic in the population and the underdetection of cases in community surveillance [28, 40, 41].*

*The findings underline the need to further explore, using robust evaluation methods, ED strategies for the detection* *of SARS-CoV-2 or asymptomatically transmitted respiratory viruses to better define the observatory role of EDs in emerging epidemics.”*

(revised manuscript, page 18, lines 436 to page 20, lines 506).

45. l.331: We have noted that this is the first time you have cited Supplementary Figure S11. Please note that all figures and tables (including those in Supporting information files) should be mentioned in the main text before the discussion.

Author’s answer

We have corrected this. S11 Appendix is mentioned in the Results section.

### FIGURES

46. For all Figures, please ensure that you have complied with our figures requirements <http://journals.plos.org/plosmedicine/s/figures>.

Please provide titles and legends for all figures (including those in Supporting Information files).

Please consider avoiding the use of red and green in order to make your figure more accessible to those with colour blindness.

Author’s answer

Titles and legends for all figures have been provided in the manuscript, including in the Supporting Information file.

47. In the flow diagram, please indicate the number of individuals in each group analyzed in the ITT analysis.

Author’s answer

A legend was added to Fig2. Flow diagram, as follows:

“*For the primary outcome, all patients were analyzed according to the intent-to-treat principle.”*

(revised manuscript, page 14, line 338).

48. Please in the figure legend/description, define abbreviations used in each figure (including those in Supporting Information files).

Author’s answer

All the abbreviations used in the figure legends and descriptions, including in Supporting Information files (revised manuscript, pages 27-29), have been defined.

49. Figure 1: Please check if ‘1^st^ period’ and ‘2^nd^ period’ need to be swapped in the right arm of the flowchart. As it stands, all Emergency Departments would have followed the intervention strategy during the 1^st^ period of the trial.

Author’s answer

Thank you for this comment. The mistake was corrected in the revised version of the figure.

50. Figure 1: Please change the ‘(18 years old or older)’ to ‘(≥ 18 years old)’.

Author’s answer

The figure was revised accordingly.

51. Figure 2: Please define the ‘rapid molecular tests’ and ‘rapid multiplex respiratory virus tests’ in the figure legend. Are the ‘multiplex tests” (248 patients box) the same as the “rapid multiplex respiratory virus tests”? Please use the same descriptions to avoid confusion.

Author’s answer

The same terms have been used in the figure to describe the tests, namely, ‘rapid molecular tests’ and ‘rapid multiplex tests’.

Both tests are described in the figure legends, as follows:

*“**The ‘rapid multiplex test’ refers to the multiplex polymerase chain reaction assay QIAstat-Dx respiratory SARS-CoV-2 panel. The ‘rapid molecular test’ refers to the Abbott IDNOW COVID-19 assay, an isothermal nucleic acid amplification based on a nicking and extension amplification reaction (NEAR) technique.”*

(revised manuscript, page 14, lines 341-343).

52. Figure 3: The figure title ‘Calendar’ is a very vague description of the figure. Please revise.

Author’s answer

Thank you for your comment. The title of Figure 3 has been changed as follows:

*“Fig3. Study periods by emergency department and weekly incidence rate”*

(revised manuscript, page 15, line 369).

TABLES

53. Please note the use of commas to separate upper and lower bounds, as opposed to hyphens as these can be confused with reporting of negative values.

Author’s answer

The text of the manuscript and of the eSupplement has been checked to ensure that it corresponds to the recommendation.

54. Suggest reporting statistical information as detailed above – see under ABSTRACT

Author’s answer

Please refer to the answer to previous comments #8 and #10.

55. Please provide titles and legends for all tables (including those in Supporting Information files).

Author’s answer

Titles and legends for all tables have been provided in the manuscript, including in the Supporting Information file.

56. Please define all abbreviations used in the table below each table (including those in Supporting Information files).

Author’s answer

All the abbreviations used in the table legends and descriptions, including in Supporting Information files, have been defined.

57. Table 1: Please define ‘med‘ and ‘Q‘.

Table 2: Please define ‘CI’.

Author’s answer

The abbreviations ‘med‘, ‘Q‘ and ‘CI’ were defined.

SUPPLEMENT

58. eSupplement S3: The footnote ‘b’ is not in the actual table (mistakenly marked as ‘a’ following ‘(days)’). Please revise. In addition, please define ‘IQR’.

Author’s answer

Thank you for the correction. The footnote ‘b’ was revised. The abbreviation ‘IQR’ was also revised.

59. eSupplement S4: Under ‘Medical Coverage’, please change ‘or Autres’ to ‘or other’. Please define ‘CI’, ‘med’, ‘Q’.

eSupplement S5: Under ‘Medical Coverage’, please change ‘or Autres’ to ‘or other’. Please define ‘CI’, ‘med’, ‘Q’, ‘ED’.

eSupplement S5: For ‘Fever (>38°) or feeling Feverish’, please add a unit (Celsius).

eSupplement S6: Please define ‘ED’ or change to ‘Emergency department’.

eSupplement S7: Please define ‘y.o.’.

eSupplement S8: Please define ‘med’, ‘Q’, ‘CI’.

eSupplement S9/eTable 1: Please define ‘CI’.

eSupplement S9/eTable 2: Please define ‘SE’.

eSupplement S9/eTable 3/5/6: Please define ‘SE’, ‘CI’.

eSupplement S9/eTable 4: Please define ‘ED’, ‘SE’, ‘CI’.

eSupplement S10: Please define ‘ED’, ‘CI’.

Author’s answer

The text has been revised accordingly.

60. eSupplement S11: Please define ‘ED’. Please increase the size of the figure legend and the axis label. Please revise throughout the entire manuscript.

Author’s answer

The abbreviation ‘ED’ has been described. The figure legend and axis label were also corrected accordingly in the entire manuscript.

REFERENCES

61. PLOS uses the numbered citation (citation-sequence) method and first six authors, et al.

Please ensure that journal name abbreviations match those found in the National Center for Biotechnology Information (NCBI) databases (<http://www.ncbi.nlm.nih.gov/nlmcatalog/journals>), and are appropriately formatted and capitalised.

Please also see <https://journals.plos.org/plosmedicine/s/submission-guidelines#loc-references> for further details on reference formatting.

Where website addresses are cited, please specify the date of access.

Author’s answer

The references were checked to ensure that they complied with PLOS Medicine guidelines.

# REVIEWER #1 COMMENTS

The Authors present an very interesting manuscript about the value of screening for asymptomatic or paucisymptomatic patients in addition to routine screening for the detection of SARSCoV-2 infection in the emergency departments (EDs). The intensified screening strategy took place in the year 2021 in EDs of the Paris metropolitan area, in addition to routine screening practice. The intensified screening strategy consisted of a questionnaire about risk exposure and symptoms offered by nurses to asymptomatic/paucisymptomatic patients from February 17, 2021, to May 31, 2021. The primary outcome was the proportion of newly diagnosed SARS-CoV-2-positive patients among all adults visiting EDs.

During the intervention period, 17,512 patients were screened in routine practice, resulting in 1,635 (9.3%) new SARS-CoV-2 diagnoses while 4,283 patients were screened with the intensified screening strategy , resulting in 224 new diagnoses (5.2%). In total, 1,859 patients were newly diagnosed among 69,248 ED admitted patients. The Authors conclusion is that an intensified ED screening strategy is unlikely to identify a substantial proportion of new diagnoses if the intensified screening is aimed at asymptomatic patietns who are unlikely to refer to ED. This data is functional to the concern that adding SARS CoV-2 screening in ED will have unintended consequences for ED workload, work capacity and length of stay.

1. I would ask the Authours a brief comment in the discussion on the cost of using the ID NOW molecular platform in ED setting in their designed strategy of intensifying SARS CoV-2 testing.

Author’s answer

We thank you for this encouraging comment.

As recommended, we have included this interesting suggestion in the discussion section, as follows:

*“A rapid isothermal amplification assay was used for biosafety reasons and rapid turnaround time [22]. Although the performances of such assays might be questionable compared to PCR techniques, several studies evaluating the same test we used reported high specificity (>97.5%) and high sensitivity (>98.5%) provided that tests were performed with dry swabs after nasopharyngeal sampling [36-39]. The test provides qualitative results, and therefore, residual and presymptomatic infections could not be distinguished. The aim was also to choose a test that would be easy and safe for nurses with no prior experience or qualifications in laboratory testing. The test had to provide quick results, similarly to lateral flow assays, possess a higher sensitivity for identifying asymptomatic individuals and offers result traceability. Finally, rapid isothermal assays offer testing options that may be more affordable than single automated real-time PCR assays, particularly in terms of cost of the system.*

*For reasons of* *feasibility, our study could not include an evaluation of the costs in the two groups compared and an examination of the choice of tests. This is a major area since decisions about the adoption of control measures must reflect both effectiveness and costs [9].”*

(revised manuscript, page 19, lines 485-498).

# REVIEWER #2 COMMENTS

This manuscript by Leblanc et al. evaluates an expanded and targeted SARS-CoV-2 screening strategy intervention in 18 EDs in the Paris region using a cluster-randomized, two-period, crossover trial serendipitously performed over a major peak of regional SARS-CoV-2 transmission between February and June of 2021. The authors demonstrate that their expanded screening strategy did not identify a significantly higher proportion of new cases compared to the control screening methodology during this dynamic interval of rapidly changing disease prevalence. The trial was expertly performed, the analyses are thorough and robust, and the manuscript is well-written.

Author’s answer

We thank you for the encouraging and interesting remarks that enabled us to clarify the text.

I offer the following suggestions to improve the breadth of interpretations in the discussion. In response to a previous round of reviews, the authors have appropriately tempered initial claims of systematic screening and used the terminology "intensified screening."

1. The authors' study seems most consistent with a "targeted" screening strategy as it relies upon nurse-directed implementation of a questionnaire prior to selecting an appropriate screening test for the disease of interest. As others have previously shown in a low prevalence disease setting (doi: 10.1001/jamanetworkopen.2021.17763), targeted screening strategies do not typically increase the number of new diagnoses when the prevalence of disease is low but do reduce the number of tests administered to the population of interest.

The present study was performed during a period of very high disease prevalence and an untargeted study design (free testing offered to anyone without the implementation of an initial questionnaire) may have achieved much higher than 13.6% participation in the screening process which may have corresponded to more cases diagnosed. While previously published data evaluating low-prevalence disease settings do not suggest that this would have changed the authors results or conclusions, it is important to note in the discussion that the 13.6% participation rate was potentially lower than it otherwise might have been due to the targeted nature of the screening process.

Author’s answer

The study consisted of offering a questionnaire and a screening test to **all** patients presenting to the ED, apart from symptomatic patients and hospitalized patients already benefiting from routine screening.

The questionnaire included questions on socio-demographic characteristics, SARS-CoV-2 symptoms, and risk exposure. The purpose of the questionnaire was to describe the characteristics of the population screened and not to select patients according to their risk factors or symptoms, as is the case with a targeted screening strategy.

Symptoms were recorded in the questionnaire to guide the choice of test, which differed according to the patient's asymptomatic or paucisymptomatic profile.

The offer of a questionnaire to 13.6% of eligible patients was achieved, resulting in a total of 4,283 tests performed. In the discussion, we pointed out that these results are inherent to screening strategies in EDs, where the proportion of patients involved rarely exceeds 20%, as we observed in 2 previous studies carried out in the same EDs [24, 35]. Furthermore, our findings were obtained in a context of high tension on resources after a year of the COVID-19 pandemic which limited the availability of nursing teams for SARS-CoV-2 screening. In addition, during the course of the study, a large proportion of patients (25% of the ED patients) were routinely tested because they had COVID-19-related symptoms or were hospitalized, which resulted in a substantial screening workload.

Following your suggestion, this proportion of patients reached was more clearly presented as a limitation of the study, since the “ideal” but unrealistic objective would have been to reach 100% of eligible patients.

Please find below the revised limitation section:

*“In terms of limitations, in addition to the factors described, including the high fluctuation in the epidemic which may have reduced the impact of the intervention, during the study, 14% of the eligible ED patients completed the questionnaire, which was the first stage of the nurse-driven screening strategy for asymptomatic/paucisymptomatic individuals, 67% of whom were screened.* *This is inherent to screening strategies in EDs, in which the proportion of patients involved in the screening process rarely exceeds 20% [24, 35].”*

(revised manuscript, page 19, lines 478-483).

2. It is also unclear in the setting of a very high prevalence disease setting if increased participation rates might have changed the results in favor of universal screening - emphasizing the importance of being very clear that the present study evaluated a targeted screening strategy rather than an untargeted strategy. Clearly, the authors had no way to know during study planning that the study interval would be perfectly timed to coincide with the peak of a major outbreak of disease, therefore their design of a targeted screening strategy was completely justified, and this is not a major study limitation - just one that should be made as transparent as possible in the discussion.

Author’s answer

As we indicated in the previous response to your comment #1, the screening approach cannot be considered targeted. However, as you point out, we could not have known that the study interval would coincide with a peak in the epidemic and a lockdown period in the country. In this context, the choice of study design with cluster randomisation and crossover was appropriate, as was the inclusion of the period and incidence rate in the analysis.

This was mentioned in the discussion, as follows:

*“Despite a trend in the primary outcome analysis toward an increased detection of infections with the intervention,* *the 95% CI provided no support for values of importance, ranging from an 11% increase, which is of* *clinical interest, to a 6% decrease. Several reasons may explain these findings.*

*(…) Second, the findings could be related to a* *highly fluctuating evolution of the COVID-19 epidemic during the study [7]. During the control period, for a similar flow, more tests appeared to be ordered for symptomatic patients or inpatients than during the intervention period (3445 versus 2903), with a positivity proportion that appeared to be higher (18.5% versus 16.6%).* *The changing epidemiological context alongside the lockdown period (April 03, 2021 to May 03, 2021) was probably imperfectly captured in the analysis, which confirms a weekly period effect and an interaction with the incidence rate. The lockdown must have led to changes with probably reduced outpatients’ number (S11 Appendix). The reduction in ED visits during the wave may have limited the role of EDs as an observatory of the epidemic.*

*(…) The results of the present study raise the issue of the study design choice in a changing epidemiological landscape. The alternative of a cluster randomization and crossover could have been a before-and-after design, but this would have resulted in difficult-to-control biases and a lower level of evidence; therefore, the chosen design is not challenged.”*

(revised manuscript, page 17, lines 414-430 and page 19, lines 469-472).

3. The authors should address in the discussion their results that further support that their screening methodology was ultimately targeted - especially the fact that the factor most strongly associated with performing screening for asymptomatic/paucisymptomatic patients was the presence of mild symptoms (OR 2.1-3.7, line 290).

Author’s answer

We refer to the answer to your previous comment #1 mentioning the non-targeted screening strategy. As indicated in answer to your comment #1, the symptoms were recorded in order to select the screening test. The presence of mild symptoms did not guide the screening offer since most of the patients screened (81%) had no symptoms. In response to your comment, this finding has been added to the Results section for greater clarity, as follows:

*“Among the asymptomatic/paucisymptomatic patients screened, 80.9% did not report symptoms and 41.6% reported not having been tested previously.”*

(revised manuscript, page 15, lines 362-363).

As you pointed out, patients who accepted the test were more likely to have symptoms than those who refused; they were also more likely to perceive themselves as being at risk and not to have been tested previously, as reported in the multivariate analysis in the Results section. This reflects the fact that the acceptance rate was better among patients who perceive themselves to be more at risk, as we had previously observed in studies on screening in EDs (Leblanc et al, Ann Emerg Med, 2018; d’Almeida et al., Arch Inter Med, 2012).

4. The higher frequency of disease found in the 4,283 screening tests offered during the intervention period when compared with the 3.4 million screening tests performed in the Paris Metropolitan region in the same time interval also strongly suggests that there was some selection bias in who received the testing. Whether this was a location-specific bias because all these patients were seen in an acute care medical setting or if this was related to the selection bias of the specific patients who chose to take / were offered the questionnaire cannot be deduced from the present data. Nevertheless, being clear in the discussion that these results together with the study design suggest that the screened population was targeted will allow the practicing ED clinician to realize that the present results are only generalizable to a targeted screening strategy implemented during a high-prevalence interval during a pandemic/epidemic wave rather than to an untargeted screening strategy implemented at those same times or to a targeted screening strategy implemented at a time of low disease prevalence.

Author’s answer

We agree that there is a selection bias regarding the beneficiaries of the screening since it was offered to ED patients, which may include a population little reached by other screening systems and who are at greater risk of SARS-CoV2.

This was more clearly discussed in the dedicated section, please refer to the previous answer to comment #44 of the Academic Editor.

A second factor that could lead to a higher prevalence among patients tested in EDs than in the general population is that ED patients who perceived themselves to be more at risk were more willing to be tested, an observation also made in other contexts of screening in EDs.

For the discussion on the characterization of our study as targeted or generalized, see answer to comment #1.

5. While not a limitation of the authors present study, the authors should consider adding a paragraph to the discussion of the consequences of implementing a test such as the rapid isothermal amplification assay with a specificity of > 97.5% during a period of low disease prevalence. They appropriately address this last comment from the previous reviewer #1 by stating that it is not an issue in their study where there was such high prevalence of disease. However, those who are contemplating expanding asymptomatic screening during periods of low disease prevalence, as that reviewer appeared to be advocating, will need to consider tests with only the highest specificity or the proportion of false positive results can rapidly diminish the utility of any screening strategy. The authors should comment how any future considerations of expanded screening for viral respiratory diseases should consider both the prevalence of disease and the specificity and sensitivity of individual tests in addition to cost-effectiveness. This has become acutely apparent in the past several months as some hospital systems continue to insist upon universal SARS-CoV-2 screening strategies for admitted patients when the prevalence of disease has diminished substantially.

Author’s answer

We have revised the discussion along these lines and clarified that the prevalence of the disease must be considered as well as the specificity and sensitivity of the tests, and the cost-effectiveness ratio.

Please refer to the answer to comment #1 of the reviewer #1.

The discussion section was also revised, as follows:

*“Screening yield can also be limited by staff shortages, costs, or testing capacities, encouraging adaptation of screening practices for asymptomatic inpatients and similarly for outpatients to individual-level risk assessment and regional incidence [13, 16].”*

(revised manuscript, page 19, lines 475-477).

# REVIEWER #3 COMMENTS

Statistical review

This paper reports a cluster-crossover trial evaluating a screening strategy in emergency departments for covid. It is shown that this doesn't significantly change the number of infections identified. The trial is analysed with appropriate methods and is reported very well.

Author’s answer

We thank you for the comments and appreciate your remarks. All the comments have been addressed as detailed below.

I have some minor comments:

1. Abstract: In the findings paragraph it would be useful to add more about the number screened in the Paris metropolitan region.

Author’s answer

Following your comment and that of the Academic Editor, additional information regarding screening data in the Paris metropolitan region, including the number of individuals screened, has been added for more clarity. Please refer to the answer to comment #1 of the Academic Editor.

2. Methods: I also found information lacking about the screening in Paris metropolitan region - was this routinely collected data or conducted as part of the research study? It's possible this estimate was treated as a known, fixed value in which case I would recommend this is made clearer in the paper.

Author’s answer

The text has been clarified following your suggestion. Please refer to the answer to comment #1 of the Academic Editor.

3. Statistical analysis: where 'patient characteristics' are referred to, I would recommend these are provided somewhere.

Author’s answer

The demographic characteristics of the study population have been added to the Abstract and Results sections. Please refer to the answer to the Academic Editor’s comment #16.

4.	Page 7: "estimated using the KR or containment method." - what affected which method was used?

Author’s answer

Thank you for your comment. The text has been corrected. For both models, the parameters were estimated using the Laplace method and the fixed effects were estimated using the containment method.

Please find below the revised part of the Methods section:

*“In both models described (…) Parameters were estimated with the Laplace method, and the fixed effects were estimated using the containment method.”*

(revised manuscript, page 11, lines 278-279).

5.	Page 8: The "Miettinen-Nurminen (MN)" method was new to me, can more be said about what this does and justifying its use? Is this because the data available was a 2x2 table?

Author’s answer

A score confidence interval has been proposed by Miettinen and Nurminen for comparing proportions (2x2 contingency table). The Miettinen-Nurminen confidence limits for the risk difference are computed by inverting score tests for the difference (see Miettinen O and Nurminen M. Comparative analysis of two rates. Stat Med 1985). This method introduces an unbiased estimate of the variance of the difference. This method performs well in several situations and is robust with large samples or unequal sample sizes. It is recommended in the publications described below and is implemented in SAS software. For these reasons, in our study, the differences and 95% CIs for the qualitative data in the secondary outcomes were estimated using the Miettinen-Nurminen method.

The references have been added to the manuscript as described below.

*“Statistical analysis (...) The proportions of new SARS-CoV-2 diagnoses (…) were compared to the proportions (…) using the Miettinen-Nurminen (MN) method [25-27] for risk difference and 95% confidence interval (CI) estimations.*

(revised manuscript, page 11, lines 282-287).

The following references have been added:

- Miettinen O, Nurminen M. Comparative analysis of two rates. Stat Med. 1985;4(2):213-26.
- Laud PJ, Dane A. Confidence intervals for the difference between independent binomial proportions: comparison using a graphical approach and moving averages. Pharm Stat. 2014;13(5):294-308.
- Fagerland MW, Lydersen S, Laake P. Recommended confidence intervals for two independent binomial proportions. Stat Methods Med Res. 2015;24(2):224-54.

We can also find a recommendation of the method in W. Garner, Constructing Confidence Intervals for the Differences of Binomial Proportions in SAS®, 2007.

6.	Page 8: "calculated by profile (diagnoses through screening for asymptomatic…". I would recommend clarifying this sentence: "The proportion was calculated separately for different profiles of patients. These profiles included 'diagnoses through screening for asymptomatic/paucisymptomatic patients' and 'all cases'." If something else is meant by calculated by profile, I would make this clearer.

Author’s answer

Your comment has been accounted for, as follows:

*“The proportion of new diagnoses was considered among ED tested patients. The proportion was calculated for patients screened through ED screening for asymptomatic/paucisymptomatic patients and for all patients screened.”*

(revised manuscript, page 12, lines 293-295).

7.	Page 11: I think 'IC on line 300' should be CI.

Author’s answer

Thank you for this comment. The mistake was corrected in the revised version of the manuscript.
