# Supplementary material for: Intensified screening for SARS-CoV-2 in 18 emergency departments in the Paris metropolitan area, France (DEPIST-COVID): A cluster-randomized, two-period, crossover trial
Source: PLoS Med. 2023 Dec 7;20(12):e1004317. doi: 10.1371/journal.pmed.1004317 (PMC10735176; doi:10.1371/journal.pmed.1004317)
Supplement: Attachment — Submitted filename: Response_Editor_R2_20231025.docx [file pmed.1004317.s003.docx]

**Manuscript "Intensified screening for SARS-CoV-2 in 18 emergency departments in the Paris metropolitan area, France (DEPIST-COVID): A cluster-randomized, two-period, crossover trial"**

**(PMEDICINE-D-23-01467R2)**

**Answer to Editor comments**

Author’s answer

We thank you for your interesting and useful suggestions. All your comments have been taken into account, as detailed below.

TITLE

1. We suggest changing the title to: “Intensified screening for SARS-CoV-2 in emergency departments in Paris, France (DEPIST-COVID): A cluster-randomized, two-period, crossover trial”

Author’s answer

The title has been revised as follows:

“*Intensified screening for SARS-CoV-2 in 18 emergency departments* *in the Paris metropolitan area, France (DEPIST-COVID): A cluster-randomized, two-period, crossover trial*” (revised manuscript, page 1, lines 1-3).

The study was carried out in several departments of the region, including the city of Paris.

FINANCIAL DISCLOSURE

2. If possible, please include specific grant numbers in your funding statement.

Author’s answer

The study did not receive a grant number by the fundings agencies.

DATA AVAILABILITY STATEMENT

3. Thank you for updating your Data availability statement. We suggest changing the statement as follows: “The study data are owned by the Assistance Publique - Hôpitaux de Paris (AP-HP) sponsor, “Département de la Recherche Clinique et du Développement” and are not freely available. Requests to access the data should be directed to: DJENNAOUI Fatiha <[fatiha.djennaoui@aphp.fr](mailto:fatiha.djennaoui@aphp.fr)> and [DRC] Secretariat Promotion Délégation à la Recherche Clinique et à l'Innovation <[drc-secretariat-promotion@aphp.fr](mailto:drc-secretariat-promotion@aphp.fr)>.”

Author’s answer

The statement has been changed according to your comment.

ABSTRACT

4. We noted that you provided the CONSORT Abstract checklist as a supplementary file. You do not need to provide the actual file, but we would like to encourage you to revise your abstract once more following the CONSORT Abstract checklist. We have pointed out some specific items in the following points.

Author’s answer

The CONSORT Abstract checklist has been removed from the supplementary file.

5. Please only report the primary outcome of the trial in your abstract. We are in the process of overhauling our workflows and policies (with a new Executive Editor), and we now require that trial abstracts only report secondary outcomes if all secondary outcomes of the trial are included. For trials that have many secondary outcomes, such as yours, the abstract should be limited to reporting the primary outcome.

Author’s answer

As per your suggestion, only the primary outcome is reported in the revised version of the abstract (revised manuscript, pages 2-3, lines 38-53).

6. At the beginning of the Methods/Findings section of the abstract, please change ‘During this cluster-randomized…’ to ‘We conducted a cluster-randomized…’.

Author’s answer

The abstract has been revised accordingly (revised manuscript, page 2, line 31).

7. Please include the clinical trial registry number in the abstract.

Author’s answer

The clinical trial registry number has been added (revised manuscript, page 3, line 54).

8. In the last sentence of the Abstract Methods and Findings section, please describe the main limitation(s) of the study's methodology (e.g., “The main limitation of the study is that it was conducted in a rapidly evolving epidemiologic context.”)

Author’s answer

In response to your suggestion, the main study limitation was clearly described in the abstract (revised manuscript, page 3, lines 49-50).

9. “The ED population seems to be more affected than the population of the region” – this statement and the conclusion paragraph seem rather unclear. Please revise the conclusion paragraph for clarity and aim to focus on the study implications and the interpretation of the study based on the primary results presented in the abstract.

Author’s answer

The above statement about a secondary outcome has been removed from the abstract and the concluding paragraph has been clarified (revised manuscript, page 3, lines 52-53).

AUTHOR SUMMARY

10. The Author Summary requires revision. Under the second question ‘What Did the Researchers Do and Find?, the bullet points, particularly the second one, are rather long and we encourage you to focus on the most important outcome (i.e. the primary outcome).

Author’s answer

The Author Summary has been revised. The length, particularly of the second point, has been reduced (revised manuscript, page 4, lines 57-90).

11. To improve clarity, we also suggest changing the bullet points under ‘Why Was This Study Done?’ as follows:

• Undetected asymptomatic SARS-CoV-2 infections or paucisymptomatic infections with mild symptoms are responsible for a substantial portion of transmissions, which has been a major challenge for managing the COVID-19 pandemic.

• Free and widely available screening has been offered in many countries to reach asymptomatic or paucisymptomatic infectious individuals and improve detection, reduce transmission, and help contain the pandemic.

• The value of systematically offering screening during medical consultations, particularly in emergency departments (EDs), as a possible strategy to identify asymptomatic/paucisymptomatic infectious individuals has, to our knowledge, not been evaluated.

Author’s answer

The section has been revised accordingly (revised manuscript, page 4, lines 58-67).

12. The last bullet under ‘What Do These Findings Mean?’ point should describe the main limitation of the study's methodology.

Author’s answer

The main study limitation was added to the last point of the section (revised manuscript, page 4, lines 82-90).

INTRODUCTION

13. ll.119-120, suggest: A recent systematic review also pointed out these studies were modeling studies or retrospective observational analyses not conducted in a controlled environment [9].

Author’s answer

The sentence has been revised accordingly (revised manuscript, page 5, lines 107-108).

14. l.137: Please add the time point for the “vaccine roll-out”.

Author’s answer

The time point has been added (revised manuscript, page 6, line 125).

METHODS AND RESULTS

15. ll.317-320: There seems to be a formatting issue – please revise.

Author’s answer

The text has been revised (revised manuscript, page 12, lines 302-311).

FIGURES

16. Figure 3: Please include in the figure description an explanation of the meaning of the black line.

Author’s answer

In Figure 3, a legend has been added for the black line corresponding to the weekly incidence rate per 100,000 individuals >= 20 y.o. in the Paris Metropolitan area (revised Figure 3).

CHECKLIST

17. Thank you for providing your Consort 2010 statement: extension to cluster randomised trials checklist. Please replace the page numbers with paragraph numbers per section (e.g. "Methods, paragraph 1"), since the page numbers of the final published paper may be different from the page numbers in the current manuscript.

Author’s answer

Paragraph numbers per section have been provided (revised S12 Appendix).

SUPPLEMENT

18. eSupplement S9/eTable 6: Please remove the ‘E’ from ‘Standard Error E’.

Author’s answer

The text has been revised (revised eSupplement S9).

19. eSupplement S11 Figure 1: I assume that the dots/lines for "lockdown 2021" are not meant to show values, but to represent the time period of the lockdown. I would suggest using a shaded area in the background instead of the dots/line to mark this time period so that it is not confused with the actual values displayed with the other dots and lines for 2019 and 2021.

Author’s answer

I fully agree with your suggestion, which clarifies the figure (revised S11 Figure 1).

20. In Figures eSupplement S11 Figure 1 and Figure 2, please show the axis beginning at zero. If this is not possible, please show a break in the axis.

Author’s answer

In eSupplement S11, Figure 1 has been revised to take account of your request (revised S11 Figure 1).

For Figure 2, due to the incompatibility of R packages, the addition of a break on the x-axis made it impossible to define the y-axis, resulting in a figure that was more difficult to read and unsatisfactory (y-axis starting before 0 and impossibility of manually positioning a 0 on the x-axis).

The original version has therefore been retained. We hope you will agree with this proposal.

Here is the initial version of the Figure:

Here is the reworked version of the Figure, which is not satisfactory:

SOCIAL MEDIA

21. To help us extend the reach of your research, please provide any X (formerly known as Twitter) handle(s) that would be appropriate to tag, including your own, your coauthors’, your institution, funder, or lab. Please respond to this email with any handles you wish to be included when we tweet this paper.

Author’s answer

Here are X identifiers that can be used:

@Leblanc_Judith

@LEGOFF_JEROME

@Chauvin_Antho

@karlahemming

@vcolizza

@chiaresabbatini

@FhuIMPEC

@APHP

@agenceANRS

@FaculteSanteSU

@iplesp

@viroSLS
